# A High-resolution Typing Assay for Uropathogenic *Escherichia coli* Based on Fimbrial Diversity

**DOI:** 10.3389/fmicb.2016.00623

**Published:** 2016-04-29

**Authors:** Yi Ren, Agata Palusiak, Wei Wang, Yi Wang, Xiao Li, Huiting Wei, Qingke Kong, Antoni Rozalski, Zhi Yao, Quan Wang

**Affiliations:** ^1^Department of Immunology, Tianjin Key Laboratory of Cellular and Molecular Immunology, Key Laboratory of Educational Ministry of China, School of Basic Medical Sciences, Tianjin Medical UniversityTianjin, China; ^2^Shanghai Majorbio Bio-pharm Biotechnology Co., Ltd.Shanghai, China; ^3^Department of General Microbiology, Department of Immunobiology of Bacteria, Institute of Microbiology, Biotechnology and Immunology, University of LodzLodz, Poland; ^4^Key Laboratory of Molecular Microbiology and Technology, Ministry of Education – Tianjin Key Laboratory of Microbial Functional Genomics, TEDA Institute of Biological Sciences and Biotechnology, Nankai UniversityTianjin, China; ^5^Institute of Preventive Veterinary Medicine, Sichuan Agricultural UniversityChengdu, China; ^6^State Key Laboratory of Medicinal Chemical Biology, NanKai UniversityTianjin, China

**Keywords:** Uropathogenic *Escherichia coli*, fimbriae, typing, lineages, epidemiological surveillance

## Abstract

Urinary tract infections (UTIs) are one of the most common bacterial infections in humans, causing cystitis, pyelonephritis, and renal failure. Uropathogenic *Escherichia coli* (UPEC) is the leading cause of UTIs. Accurate and rapid discrimination of UPEC lineages is useful for epidemiological surveillance. Fimbriae are necessary for the adherence of UPEC strains to host uroepithelia, and seem to be abundant and diverse in UPEC strains. By analyzing all the possible fimbrial operons in UPEC strains, we found that closely related strains had similar types of chaperone-usher fimbriae, and the diversity of fimbrial genes was higher than that of multilocus sequence typing (MLST) genes. A typing assay based on the polymorphism of four gene sequences (three fimbrial genes and one housekeeping gene) and the diversity of fimbriae present was developed. By comparison with the MLST, whole-genome sequence (WGS) and *fumC/fimH* typing methods, this was shown to be accurate and have high resolution, and it was also relatively inexpensive and easy to perform. The assay can supply more discriminatory information for UPEC lineages, and have the potential to be applied in epidemiological surveillance of UPEC isolates.

## Introduction

Urinary tract infections (UTIs) are one of the commonest bacterial infections causing morbidity in humans. Lower UTIs usually induce cystitis and can progress to upper UTIs, resulting in pyelonephritis and ultimately renal failure (Foxman, 2003; Nielubowicz and Mobley, 2010; Ulett et al., 2013). It is estimated that 40% of women and 12% of men will experience a symptomatic UTI during their lifetime (Nielubowicz and Mobley, 2010); infants and children are also susceptible (Foxman, 2003; Zorc et al., 2005; Guay, 2008). There are >100 million cases of UTIs annually worldwide, causing a serious economic and medical burden (Shaikh et al., 2008; Chakupurakal et al., 2010). In the US alone, UTIs cause about 10 million physician visits and more than 1 million emergency room visits, with a cost more than 3 billion dollars annually (Foxman and Brown, 2003; Scholes et al., 2005; Litwin and Saigal, 2007; DeFrances et al., 2008). Uropathogenic *Escherichia coli* (UPEC) is the leading cause of UTIs, accounting for most community (∼95%) and hospital acquired (∼50%) infections (Foxman and Brown, 2003; Kucheria et al., 2005; Jacobsen et al., 2008).

Accurate and rapid classification of UPEC is important to define bacterial subpopulations, which is useful for epidemiological surveillance of rapidly spreading drug-resistant clones and prevalence of high risk clones. Multilocus sequence typing (MLST) is a commonly used method for characterizing the relationship of strains within bacterial species, and certain sequence types (STs, profiles identified by MLST) of pathogenic bacterial isolates are epidemiologically associated with specific syndromes (Manges et al., 2001; Johnson et al., 2006, 2008). A definite number of *E. coli* lineages such as the global pandemic clone, sequence type 131 (ST131), and other frequently described ST95, ST69, ST73, and ST127 have been reported to induce most UTIs (Rogers et al., 2015). Some useful techniques such as Fourier transform infrared (FT-IR) spectroscopy (AlRabiah et al., 2013; Dawson et al., 2014), ultraviolet resonance raman (UVRR) spectroscopy (Jarvis and Goodacre, 2004), and matrix-assisted laser desorption/ionization time-of-flight (MALDI-TOF) mass spectrometry (Kohling et al., 2012), have also been developed for the identification of UPEC isolates. Recently, whole-genome sequence (WGS) data were used for identification and classification of bacterial strains within species such as *Staphylococcus aureus* and *Streptococcus suis*, providing information for diagnosis, epidemiological investigation and clinical care with high-resolution (Pallen et al., 2010; Dunne et al., 2012; Chen et al., 2013).

Fimbriae appear as hair-like appendages protruding from bacterial surfaces and mediate diverse functions such as adherence and biofilm formation (Eden and Hansson, 1978; Proft and Baker, 2009). In Gram-negative bacteria, fimbriae are assembled via different protein translocation systems, of which the chaperone-usher pathway is the most frequent (Kline et al., 2009). The chaperone-usher pathway is a highly conserved bacterial secretion system, which requires a periplasmic chaperone and an outer membrane assembly platform called the usher (Sauer et al., 2004). Fimbrial adhesins, located at the tip, recognize specific receptor targets and enable bacterial adherence to specific surfaces of the host (Proft and Baker, 2009). Based on published genome sequence data, about 10 chaperone-usher fimbrial operons have been identified in different UPEC isolates (Wurpel et al., 2013).

*In vitro* and *in vivo* analyses have suggested that fimbriae play an important role in UPEC adherence and invasion of human bladder and kidney cells (Leusch et al., 1991; Eto et al., 2007), which is an important stage in the pathogenesis of UTIs. It has also been reported that some fimbrial genes such as *fimH* are polymorphic, and point mutations in such genes are very significant for function (Weissman et al., 2006, 2007, 2012; Dreux et al., 2013), A method based on the polymorphisms of *fimH* and two genes – *fimH* and *fumC* has been developed for the typing of *E. coli* strains, and shown to have a superior discrimination power than MLST (Dias et al., 2010; Weissman et al., 2012). Therefore, it is suggested that fimbrial genes have the possibility to be used as the targets for UPEC typing.

In this study, we obtained the genome sequences of eight UPEC clinical isolates. By analyzing the previously published and newly sequenced UPEC genome sequences, fimbrial operons were identified, and the polymorphisms of genes located in the most common fimbrial operons were analyzed. Finally, an assay based on the polymorphism of three fimbrial genes and *fumC* gene and the types of fimbriae was developed for UPEC typing and was shown to be accurate and have high resolution by comparison with the MLST, WGS, and *fumC*/*fimH* typing methods using 63 UPEC strains with available genome sequences and 67 clinical UPEC isolates.

## Materials and Methods

### Bacterial Strains and Growth Conditions

All the UPEC clinical strains used in this study were listed in Supplementary Table S1. The species identification of the strains was confirmed by an API 20E test (BioMérieux, France). *E. coli* strains were grown overnight in Luria-Bertani medium at 37°C with shaking.

### Genomic DNA Extraction

Bacterial genomic DNA was extracted using a DNA extraction kit (Tiangen, Beijing, China).

### Gene Amplification and Sequencing

The whole *yagV* (657 bp), *fimF* (531 bp), and *fimH* (903 bp) genes were amplified and sequenced by primers listed in Supplementary Table S4. The genes encoding the usher proteins were amplified by primers listed in Supplementary Table S4. PCR amplification was performed in 50 μl volumes containing 1x Taq buffer (plus Mg^2+^), 0.2 mM deoxynucleoside triphosphates, 0.4 μM of each primer, 1.5 U Taq DNA polymerase, and 2 μl template DNA (approximately 500 ng). The PCR cycles used were: 95°C denaturation for 50 s, 50°C annealing for 45 s, 72°C extension for 1 min, and a final extension at 72°C for 5 min. The PCR products were purified using a DNA Purification Kit (CWBIO, Beijing, China), and sequenced by the Sanger method.

### MLST and Phylogenetic Analysis

Amplification and sequencing of the MLST loci were done according to published methods^1^ (Tartof et al., 2005). The sequences of the seven MLST genes from each strain were combined and those from different stains were aligned using ClustalX2.1 under default settings (Larkin et al., 2007). A phylogenetic tree based on the alignments was constructed using maximum likelihood in MEGA 5 with 1000 bootstrap experiments (Tamura et al., 2011). The MLST profile for each strain was assigned based on the nucleotide sequences of the seven housekeeping genes using MLST databases^2^.

### Genome Sequencing and Annotation

The paired-end reads generated using an Illimina HiSeq 2000 platform were assembled by SOAP *de novo* (Li et al., 2010). Coding sequences were predicted by Glimmer 3.0 (Delcher et al., 1999). Functional annotation was based on BLASTp, with the KEGG, Swiss-Prot, COG, and non-redundant GenBank CDS databases (Altschul et al., 1997; Kanehisa and Goto, 2000; Muller et al., 2010).

### Assignment of Orthologs and Phylogenetic Analysis

Gene orthologs were identified using OrthoMCL, with a BLAST *E*-value cutoff of 1e^−5^ and an inflation parameter of 1.5. Genes included in all genomes were assigned as orthologs. The nucleotide sequences of orthologs from each strain were combined and alignments were carried out between different strains. A phylogenetic tree based on the alignments was constructed using maximum likelihood in MEGA. 1000 bootstrap experiments were performed on the concatenated sequences to assess the robustness of the topology.

### Identification of Chaperone-Usher Fimbrial Operons

All amino acid sequences encoded by the UPEC genomes and plasmids (published previously or sequenced in this study) were used to build a local BLAST database. The 85 identified usher amino acid sequences annotated previously in UPEC (Wurpel et al., 2013) were used as an initial BLASTp query dataset to search the local BLAST database with an *E*-value cutoff of 0.1. Those with *E*-values of 0 were added to the usher database, and those with an *E*-value > 0 were used to search the Pfam databases (Finn et al., 2014) for the presence of an usher protein family domain (PF00577) and/or flanking chaperone (PF00345, PF02753, or COG3121). The UC operons identified were re-annotated by BLASTp.

### Nucleotide Polymorphism, Discriminatory Power and Phylogenetic Analysis

Nucleotide polymorphism was measured by average pairwise diversity index, π, using MEGA 5 (Tamura et al., 2011). The polymorphism plot was drawn using ProSeq 3.5 based on the series of π values across overlapping windows of 100 nucleotides with a step size of 50 nucleotides (Filatov, 2009). Discriminatory power was analyzed using Simpson’s index of diversity (*D*; Dreux et al., 2013). A phylogenetic tree based on the alignments of combined nucleotide sequences from fimbrial genes was constructed using maximum likelihood in MEGA 5 with 1000 bootstrap experiments.

### Nucleotide Sequence Accession Numbers

The sequences of eight clinical UPEC isolates were deposited in the GenBank database under accession numbers JSVK00000000, JSVL00000000, JSVM00000000, JSVN00000000, JSVO00000000, JSVP00000000, JSVQ00000000, and JSVR00000000.

## Results

### Chaperone-Usher Fimbrial Operon Identification

In order to identify the Chaperone-usher fimbrial operons present in UPEC strains, we used two sets of genome sequences, one included 11 UPEC strains whose complete genomes had been sequenced and published, and the other contained the sequences obtained in this study of eight clinical UPEC strains not closely related with the 11 UPEC strains with available genomes based on MLST analysis and *fimH* sequences (Supplementary Figure S1; **Table 1**; Supplementary Table S1). Approximately 1 gigabases of 250-bp paired-end sequence were obtained for each of the eight clinical UPEC strains, giving about 200× genome coverage per strain. After *de novo* assembly and gene prediction, 4,274 to 4,957 genes were assigned for the sequenced strains (Supplementary Table S2). The average GC content for genes in these strains was 51.7%, and the average length of the assembled sequences was 5.1 megabases.

**Table 1:** Chaperone-usher fimbrial operons identified in 19 uropathogenic *Escherichia coli* (UPEC) stains.

Strain	Number of operon types	Number of total operons	Number of intact operons	Reference
CFT073	10	11	11	Welch et al., 2002
Di2	9	9	9	Reeves et al., 2011
Di14	9	9	9	Reeves et al., 2011
536	13	13	12	Hochhut et al., 2006
F11	12	12	10	Rasko et al., 2008
EC958	10	10	10	Totsika et al., 2011
UTI89	11	11	10	Chen et al., 2006
UMN026	12	12	12	Touchon et al., 2009
IAI39	12	13	10	Touchon et al., 2009
ABU83972	11	11	9	Zdziarski et al., 2010
NA114	10	10	6	Avasthi et al., 2011
1	11	11	10	This study
3	10	10	7	This study
4	11	11	11	This study
5	8	8	8	This study
7	13	13	13	This study
8	9	9	9	This study
11	12	12	11	This study
14	11	11	10	This study

**FIGURE 1:**
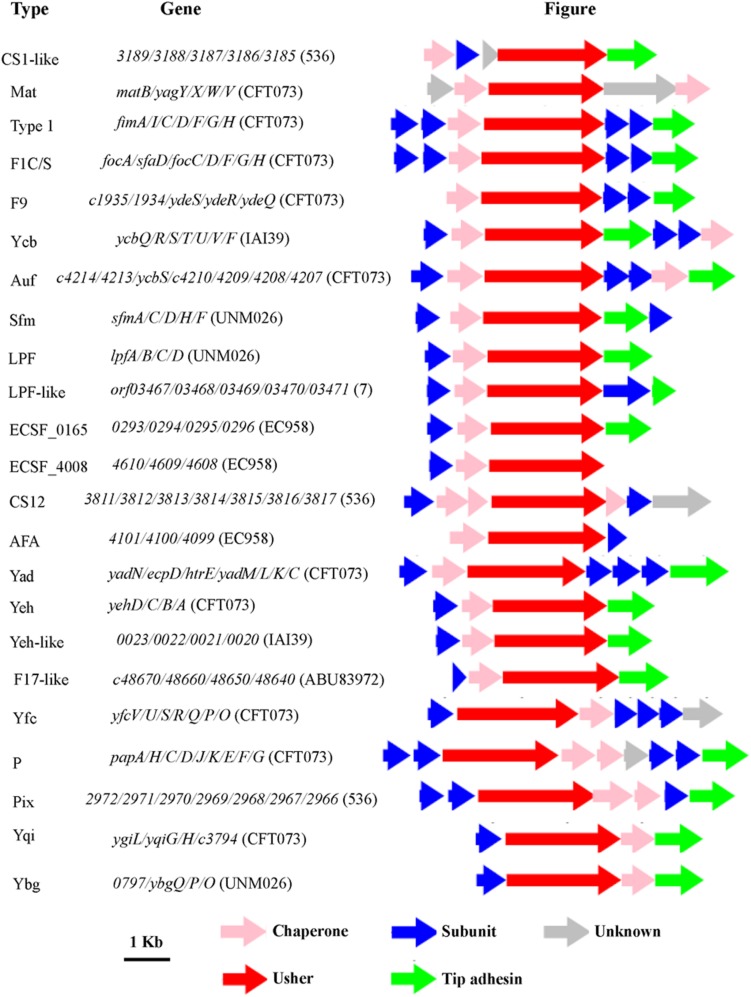
**The genetic organization of chaperone-usher fimbrial operons identified in 19 uropathogenic ***Escherichia coli*** (UPEC) strains in this study.** The scale represents DNA length 1 kilo base pair. The type of fimbriae is indicated on the left, and the genes in each fimbrial operon are indicated in the middle. The direction of the arrow indicates direction of transcription of each gene, and the color of the arrow indicates the gene function (key shown below the figure).

**FIGURE 2:**
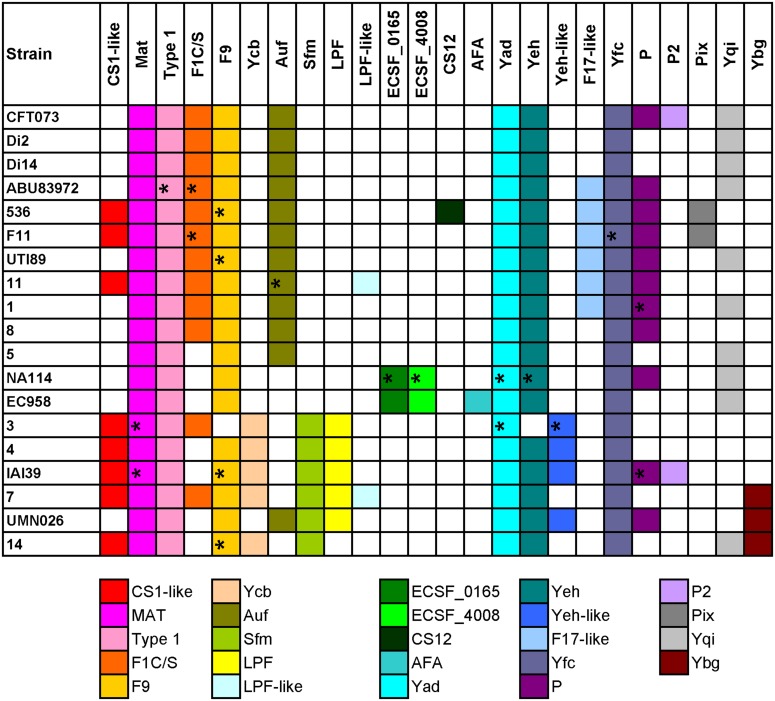
**Distribution of chaperone-usher fimbrial operons in 19 UPEC strains.** The name of the fimbriae is shown at the top. Each color box is representative of a certain type of chaperone-usher fimbrial operon, as indicated below the figure. The fimbriae operons not complete are indicated by ∗.

Chaperone-usher fimbrial operons often contain genes encoding an usher, a chaperone, and one or more fimbrial subunits. Sometimes, there are transposon insertion elements or truncated structural genes in the operons, which are considered disrupted with no function. 201 chaperone-usher fimbrial gene clusters were identified by using an usher BLASTp search against a selection of the above 19 UPEC genomes. The 19 UPEC strains contained 8 to 13 operons with an average of 11 operons per strain (**Table 1**). After annotation, it was shown that twenty-three types of chaperone-usher fimbriae were identified in the 19 UPEC strains (**Figure 1**). All of the UPEC strains contained the type 1, Mat, Yad, and Yfc fimbriae operons (certain fimbriae were not complete in some strains) and the two other fimbrial operons common in most UPEC strains were Yeh and F9 (**Figure 2**). Gene arrangement was conserved in operons of the same fimbrial type.

Four groups of strains have very similar chaperone-usher fimbriae, including, group 1: CFT073/Di2/Di14/ABU83972; group 2: 536/F11; group 3: EC958/NA114; and group 4: UMN026/IAI39/3/4/7/14. We also found some types of fimbriae were rare and present in only one of the above groups, for example, Pix in group 2, ECSF_0165 and ECSF_4008 in group 3, Sfm and/or LPF in group 4 (**Figure 2**). It was shown that strains sharing similar types of chaperone-usher fimbriae were closely related in the phylogenetic tree based on orthologs (Supplementary Figure S2). Therefore, we propose that UPEC strains with similar chaperone-usher fimbriae are evolutionarily related, and the type of chaperone-usher fimbriae present can be used as a feature to differentiate UPEC strains.

### Polymorphism Analysis of Chaperone-Usher Fimbrial Genes

We performed sequence polymorphism analysis on all genes located in the six chaperone-usher fimbriae common in most UPEC strains (Type 1, Mat, Yad, Yeh, Yfc, and F9). The π values of MLST genes (except for *fumC*) were lower than most of genes in the six chaperone-usher fimbrial operons (Supplementary Table S3; Supplementary Figure S3).

It was noted that genes in Yad and Yfc fimbrial operons had higher π values than those in the other four fimbriae. Therefore, phylogenetic trees were proposed to be constructed based on each gene in Yad and Yfc operons, and compared to those based on the MLST and homologous genes. However, it was found that the Yad genes in different strains were too distinct with different length to be aligned for constructing the phylogenetic tree (data not shown), and no gene in Yad fimbrial operon present in all 19 UPEC strains (*yadN*, *ecpD*, and *htrE* were not present in strain 3, *yadL*, *yadM*, and *yadK* were not present in strain NA114, and *yadC* was not present in strains 3 and NA114; data not shown). For Yfc genes, phylogenetic trees did not correspond well with those based on MLST and homologous genes (data not shown). In addition, genes including *yfcR*, *yfcQ*, *yfcP*, and *yfcO* were not present in all 19 UPEC strains (data not shown). Therefore, Yad and Yfc fimbrial genes could not be considered as targets for constructing phylogenetic trees for UPEC typing.

Other genes in the remaining four fimbriae (Type 1, Mat, Yeh, and F9) having higher π values than the average π value of the seven MLST genes (0.012) were considered for typing, which included *yagV* (π value = 0.016) in Mat fimbrial, *fimF* (π value = 0.02) and *fimH* (π value = 0.018) in the type 1 fimriae, *yehD* (π value = 0.020), *yehC* (π value = 0.020) and *yehB* (π value = 0.015) in Yeh fimbirae, and *c1934* (π value = 0.015), *ydeS* (π value = 0.015) and *ydeQ* (π value = 0.020) in F9 fimbriae (Supplementary Table S3). As Yeh and F9 fimbriae were not present in strain 3 (**Figure 2**), we selected three fimbrial genes including *yagV*, *fimF*, and *fimH* with higher π values than the average value of MLST genes and present in all 19 UPEC strains as candidate genes for UPEC typing. Additionally, as *fumC* has a much higher π value (π value = 0.026) than the other MLST genes and many fimbriae genes (Supplementary Table S3), it was also considered as a candidate gene for UPEC typing.

### The Typing Method Based on Polymorphism of Chaperone-Usher Fimbriae Genes and the *fumC* Gene and the Types of Chaperone-Usher Fimbriae

**FIGURE 3:**
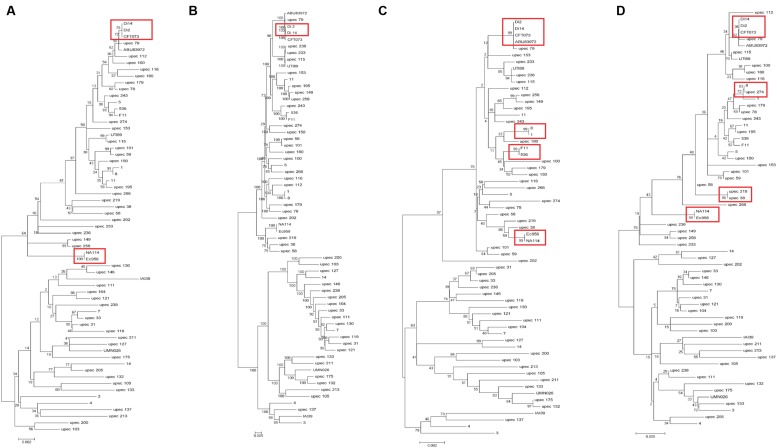
**Phylogenetic trees of 63 UPEC strains with available genome sequences based on (A) four genes (***yagV***, ***fimF***, ***fimH***, and ***fumC***), (B) homologous genes, (C) MLST genes, and (D) the internal fragments of ***fimH*** and ***fumC***.** The scales at the bottoms of the trees indicate phylogenetic distance. Bootstrap values are displayed as percentages on nodes. Closely related strains are shown in red boxes.

In addition to the above 19 UPEC strains, we also chose 44 UPEC strains from different ST types with published high-quality draft genome sequences (Supplementary Table S1). The entire sequences of the three fimbrial genes (*yagV*-657 bp, *fimF*-531 bp, and *fimH*-903 bp) and the 469 bp segment of *fumC* were combined for each strain and aligned. A phylogenetic tree based on the alignments was constructed (**Figure 3A**). The major topology was very similar to that in trees based on orthologs (**Figure 3B**) and MLST (**Figure 3C**). However, the discrimination power was better than that by MLST (differentiating strains 1 and 8, 536 and F11, and ABU83972 and CFT073, which were not separated by MLST), but worse than that by orthologs (not differentiating strains CFT073 and Di2/14, or NA114 and EC958, which were separated by orthologs).

Although strains CFT073 and Di2/14, and NA114 and EC958 could not be differentiated by genes *yagV*, *fimF/H*, and *fumC*, we found the types of chaperone-usher fimbrial operons present in these strains were different. P fimbriae present in CFT073 could not be found in Di2/14 strains. P fimbriae was present in NA114 but not EC958, AFA fimbriae was present in Ec958 but not NA114 (**Figure 2**). Therefore, typing of UPEC strains can be carried out based on genes *yagV*, *fimF/H*, and *fumC*, in combination with the types of chaperone-usher fimbrial operons present.

The two-locus clonal typing-(*fumC*/*fimH*) typing has been shown to have superior clonal discrimination power than MLST (Weissman et al., 2012). This typing method was also performed in our study. The 489 nucleotide internal fragment of *fimH* (encoding mature peptide 1 to 163, *fimH*_TR_) and the 469 nucleotide internal fragment of *fumC* of the above 63 UPEC strains were obtained, and the sequences of the two fragments were combined and aligned. A phylogenetic tree based on the alignments was constructed (**Figure 3D**). The discrimination power was better than that based on MLST but worse than that based on genes *yagV*, *fimF/H*, and *fumC* (differentiating strains 8 and upec 274, upec 219 and upec 38, which were not separated by *fumC*/*fimH*).

By the calculation of discriminatory power of different typing methods using Simpson’s index of diversity (*D*), it was shown that the methods based on WGS data and the combination of four genes (*yagV*, *fimF/H*, and *fumC*) and fimbrial types had the greatest discriminatory power [*D* = 0.999; 95% confidence interval (95% CI), 0.998–1.000], which is greater than methods based on the internal fragments of *fimH* and *fumC* (*D* = 0.997; 95% CI, 0.995–0.999), and four genes (*yagV*, *fimF/H*, and *fumC*; *D* = 0.998; 95% CI, 0.996–0.999). The typing method based on MLST had the lowest discriminatory power (*D* = 0.995; 95% CI, 0.992–0.998; **Table 2**).

**Table 2:** Numbers of types and *D*-values based on different typing methods of 63 UPEC strains with available genome sequences.

Typing method	Number of types	*D* (95% CI)
*yagV* + *fimF* + *fimH* + *fumC*	60	0.998 (0.996–0.999)
*yagV* + *fimF* + *fimH* + *fumC* + fimbrial types	62	0.999 (0.998–1.000)
WGS	62	0.999 (0.998–1.000)
MLST	57	0.995 (0.992–0.998)
*fimH*_TR_ + *fumC*	58	0.997 (0.995–0.999)

### Test of the Typing Method by Clinical UPEC Isolates

**FIGURE 4:**
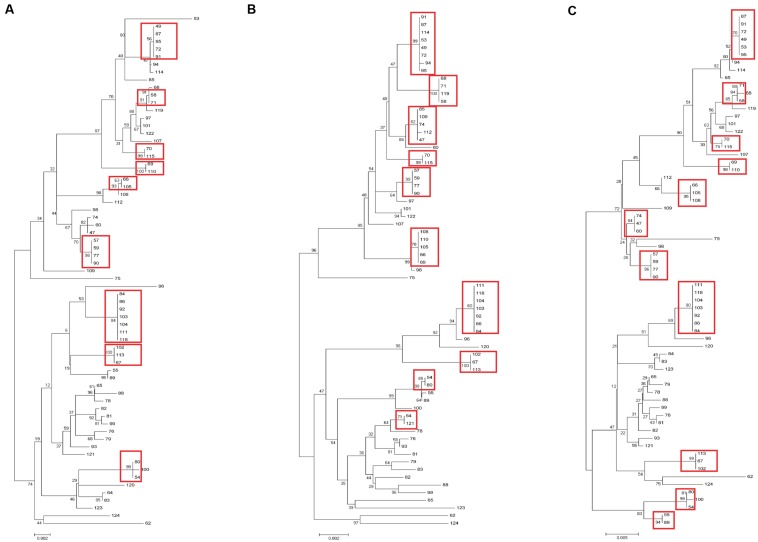
**Phylogenetic trees of 67 clinical UPEC strains based on (A) four genes (***yagV***, ***fimF***, ***fimH***, and ***fumC***), (B) MLST genes, and (C) the internal fragments of ***fimH*** and ***fumC***.** The scales at the bottoms of the trees indicate phylogenetic distance. Bootstrap values are displayed as percentages on nodes. Closely related strains are shown in red boxes.

Sixty-nine clinical UPEC isolates were used to test the typing methods developed in this study based on the four genes (*yagV*, *fimF/H*, and *fumC*) and fimbrial types (Supplementary Table S1). It was shown that 69 UPEC strains were divided into 47, 42, and 37 groups based on the polymorphism of the four genes (*yagV*, *fimF/H*, and *fumC*), the internal fragments of *fimH* and *fumC*, and MLST, respectively (**Figure 4**; **Table 3**). Strains not separated by the four genes (*yagV*, *fimF/H*, and *fumC*) were used to test their fimbrial types. Primers based on genes encoding usher proteins of the twenty-three chaperone-usher fimbriae types in UPEC strains were used (Supplementary Table S4), and strains clustered in nine groups were divided into different subgroups based on their different fimbriae types (**Figure 4**; Supplementary Figure S4). By the calculation of discriminatory power of different typing methods using Simpson’s index of diversity (*D*), it was shown that the methods based on the four genes (*yagV*, *fimF/H*, and *fumC*) and fimbrial types had the greatest discriminatory power (*D* = 0.999; 95% CI, 0.998–1.000), and the *D*-values for the four genes (*yagV*, *fimF/H*, and *fumC*), the internal fragments of *fimH* and *fumC*, and MLST were 0.980, 0.975, and 0.966, respectively (**Table 3**).

**Table 3:** Numbers of types and *D*-values based on different typing methods of 67 clinical UPEC strains.

Typing method	Number of types	*D* (95% CI)
*yagV* + *fimF* + *fimH* + *fumC*	47	0.980 (0.975–0.985)
*yagV* + *fimF* + *fimH* + *fumC* + fimbrial types	61	0.999 (0.998–1.000)
MLST	37	0.966 (0.956–0.976)
*fimH*_TR_ + *fumC*	42	0.975 (0.967–0.983)

### UPEC Types Identified in This Study

The polymorphism of the combined 2560 bp sequence including *yagV* (657 bp), *fimF* (531 bp), *fimH* (903 bp), and *fumC* (469 bp) from 67 strains with available genome sequences and 63 clinical UPEC strains were analyzed. 229 single-nucleotide polymorphisms (SNPs) were identified, and these 130 strains were separated into 99 groups (Supplementary Figure S5), in addition to the fimbriae types, 16 groups were further divided into different subgroups (**Figure 2**; Supplementary Figure S4). These groups identified by the four genes were assigned as VFHC (*yag****V***, *fim****F****/****H,*** and *fum****C***) groups 1 to 99, and several groups including subgroups (Supplementary Figure S5; Supplementary Table S5). These SNPs in addition with different fimbriae types can be used to define a UPEC strain as a same strain belonging to the 99 groups or a strain belonging to a new group.

## Discussion

As bacterial typing is valuable for epidemiological surveillance, many kinds of useful techniques have been developed for this purpose. MLST is a most widely used method for bacterial typing, which is easy to perform and inexpensive. Although it was shown to be less discriminatory by previous and this study (Weissman et al., 2012; Chen et al., 2013), MLST is still useful for epidemiological surveillance, which can supply information for global pandemic clones such as ST131 of UPEC.

Some tools including FT-IR spectroscopy, UVRR spectroscopy and MALDI-TOF mass spectrometry, which are based on “whole-organism fingerprints,” using chemometrics techniques and mathematical models, have also been developed for bacterial identification (Jarvis and Goodacre, 2004; Kohling et al., 2012; AlRabiah et al., 2013; Dawson et al., 2014). These methods are shown to have advantages such as rapid, automation, relatively low running costs and simple sample preparation, and FT-IR spectroscopy was reported to be useful for the identification of UPEC isolates such as ST131, ST95, and ST127 strains (Dawson et al., 2014). Thus, these kinds of methods can be used for the rapid identification of certain UPEC pandemic clones.

Whole-genome sequence data is of high-resolution for bacterial epidemiological typing, which can resolve a single base change between two genomes, and concentrate on the identification and exploitation of SNPs to distinguish one isolate or lineage from another (Pallen et al., 2010). For examples, genome sequences of 63 methicillin-resistant *S. aures* (MRSA) revealed geographical diversification and highlighted person-to-person transmission within the hospital setting (Harris et al., 2010; Pallen et al., 2010), and a typing method based on minimum core genome sequences was developed for the clinical medicine and epidemiological surveillance of *S. suis* (Chen et al., 2013). Sometimes, it is difficult to define clinical and epidemiological risk factors for colonization or infection with only information of bacterial ST types. For examples, the H41 subclone of ST131 is fluoroquinolone-susceptible, H30 subclone is expanded-spectrum cephalosporin-resistant, and the prevalence of these subclones is different (Rogers et al., 2015). These problems can be solved by WGS data with its great discriminatory power, however, whole genome sequencing is still too expensive for routine use.

The method developed in this study seems to have a similar discrimination power with that based on WGS data. The primers for the amplification of the four genes (*yagV*, *fimF/H*, and *fumC*) and genes encoding the usher proteins can be used for the application of the method (Supplementary Table S4), and it was shown to be inexpensive and easy to perform compared to that based on WGS data, and showed superior discrimination power. Therefore, it seems to have the potential to be applied in epidemiological surveillance of UPEC isolates, which seems to supply more discriminatory information for UPEC lineages. However, as the methods such as MLST and *fumC*/*fimH* are still widely used and have been tested by thousands of isolates, the application of the assay developed still needs further verification with much more clinical samples. Much work should be done in further for make the assay possible to be applied in clinical diagnosis. For example, the assay should be more rapid and the relationships between types, drug-resistance and prevalence should be clearly connected.

## Author Contributions

QW designed the study. AP and AR supplied the clinical UPEC strains. WW, YW, XL, and HW prepared the genomic DNA and gene products. YR, WW, QW, and QK performed the bioinformatics analysis. QW and ZY checked the data and wrote the paper. All authors analyzed and discussed the data, and reviewed the manuscript.

## Conflict of Interest Statement

The authors declare that the research was conducted in the absence of any commercial or financial relationships that could be construed as a potential conflict of interest.

## Footnotes

1 http://mlst.warwick.ac.uk/mlst/dbs/Ecoli/documents/primersColi_html

2 http://mlst.warwick.ac.uk/mlst/

## References

[B1] AlRabiah H. Correa E. Upton M. Goodacre R. (2013). High-throughput phenotyping of uropathogenic *E. coli* isolates with Fourier transform infrared spectroscopy. *Analyst* 138 1363–1369. 10.1039/c3an36517d 23325321

[B2] Altschul S. F. Madden T. L. Schaffer A. A. Zhang J. Zhang Z. Miller W. (1997). Gapped BLAST and PSI-BLAST: a new generation of protein database search programs. *Nucleic Acids Res.* 25 3389–3402. 10.1093/nar/25.17.3389 9254694 PMC146917

[B3] Avasthi T. S. Kumar N. Baddam R. Hussain A. Nandanwar N. Jadhav S. (2011). Genome of multidrug-resistant uropathogenic *Escherichia coli* strain NA114 from India. *J. Bacteriol.* 193 4272–4273. 10.1128/JB.05413-11 21685291 PMC3147708

[B4] Chakupurakal R. Ahmed M. Sobithadevi D. N. Chinnappan S. Reynolds T. (2010). Urinary tract pathogens and resistance pattern. *J. Clin. Pathol.* 63 652–654. 10.1136/jcp.2009.074617 20501451

[B5] Chen C. Zhang W. Zheng H. Lan R. Wang H. Du P. (2013). Minimum core genome sequence typing of bacterial pathogens: a unified approach for clinical and public health microbiology. *J. Clin. Microbiol.* 51 2582–2591. 10.1128/JCM.00535-13 23720795 PMC3719615

[B6] Chen S. L. Hung C. S. Xu J. Reigstad C. S. Magrini V. Sabo A. (2006). Identification of genes subject to positive selection in uropathogenic strains of *Escherichia coli*: a comparative genomics approach. *Proc. Natl. Acad. Sci. U.S.A.* 103 5977–5982. 10.1073/pnas.0600938103 16585510 PMC1424661

[B7] Dawson S. E. Gibreel T. Nicolaou N. AlRabiah H. Xu Y. Goodacre R. (2014). Implementation of Fourier transform infrared spectroscopy for the rapid typing of uropathogenic *Escherichia coli*. *Eur. J. Clin. Microbiol. Infect. Dis.* 33 983–988. 10.1007/s10096-013-2036-0 24399364

[B8] DeFrances C. J. Lucas C. A. Buie V. C. Golosinskiy A. (2008). *National Hospital Discharge Survey.*Hyattsville, MD: National health statistics reports. 18841653

[B9] Delcher A. L. Harmon D. Kasif S. White O. Salzberg S. L. (1999). Improved microbial gene identification with GLIMMER. *Nucleic Acids Res.* 27 4636–4641. 10.1093/nar/27.23.4636 10556321 PMC148753

[B10] Dias R. C. Moreira B. M. Riley L. W. (2010). Use of fimH single-nucleotide polymorphisms for strain typing of clinical isolates of *Escherichia coli* for epidemiologic investigation. *J. Clin. Microbiol.* 48 483–488. 10.1128/JCM.01858-09 20018817 PMC2815601

[B11] Dreux N. Denizot J. Martinez-Medina M. Mellmann A. Billig M. Kisiela D. (2013). Point mutations in FimH adhesin of Crohn’s disease-associated adherent-invasive *Escherichia coli* enhance intestinal inflammatory response. *PLoS Pathog.* 9:e1003141 10.1371/journal.ppat.1003141 PMC3554634 23358328

[B12] Dunne W. M. Jr. Westblade L. F. Ford B. (2012). Next-generation and whole-genome sequencing in the diagnostic clinical microbiology laboratory. *Eur. J. Clin. Microbiol. Infect. Dis.* 31 1719–1726. 10.1007/s10096-012-1641-7 22678348

[B13] Eden C. S. Hansson H. A. (1978). *Escherichia coli* pili as possible mediators of attachment to human urinary tract epithelial cells. *Infect. Immun.* 21 229–237. 361565 10.1128/iai.21.1.229-237.1978 PMC421981

[B14] Eto D. S. Jones T. A. Sundsbak J. L. Mulvey M. A. (2007). Integrin-mediated host cell invasion by type 1-piliated uropathogenic *Escherichia coli*. *PLoS Pathog.* 3:e100 10.1371/journal.ppat.0030100 PMC1914067 17630833

[B15] Filatov D. A. (2009). Processing and population genetic analysis of multigenic datasets with ProSeq3 software. *Bioinformatics* 25 3189–3190. 10.1093/bioinformatics/btp572 19797407 PMC2778335

[B16] Finn R. D. Bateman A. Clements J. Coggill P. Eberhardt R. Y. Eddy S. R. (2014). Pfam: the protein families database. *Nucleic Acids Res.* 42 D222–D230. 10.1093/nar/gkt1223 24288371 PMC3965110

[B17] Foxman B. (2003). Epidemiology of urinary tract infections: incidence, morbidity, and economic costs. *Dis. Mon.* 49 53–70. 10.1067/mda.2003.7 12601337

[B18] Foxman B. Brown P. (2003). Epidemiology of urinary tract infections: transmission and risk factors, incidence, and costs. *Infect. Dis. Clin. North Am.* 17 227–241. 10.1016/S0891-5520(03)00005-9 12848468

[B19] Guay D. R. (2008). Contemporary management of uncomplicated urinary tract infections. *Drugs* 68 1169–1205. 10.2165/00003495-200868090-00002 18547131

[B20] Harris S. R. Feil E. J. Holden M. T. Quail M. A. Nickerson E. K. Chantratita N. (2010). Evolution of MRSA during hospital transmission and intercontinental spread. *Science* 327 469–474. 10.1126/science.1182395 20093474 PMC2821690

[B21] Hochhut B. Wilde C. Balling G. Middendorf B. Dobrindt U. Brzuszkiewicz E. (2006). Role of pathogenicity island-associated integrases in the genome plasticity of uropathogenic *Escherichia coli* strain 536. *Mol. Microbiol.* 61 584–595. 10.1111/j.1365-2958.2006.05255.x 16879640

[B22] Jacobsen S. M. Stickler D. J. Mobley H. L. Shirtliff M. E. (2008). Complicated catheter-associated urinary tract infections due to *Escherichia coli* and *Proteus mirabilis*. *Clin. Microbiol. Rev.* 21 26–59. 10.1128/CMR.00019-07 18202436 PMC2223845

[B23] Jarvis R. M. Goodacre R. (2004). Ultra-violet resonance Raman spectroscopy for the rapid discrimination of urinary tract infection bacteria. *FEMS Microbiol. Lett.* 232 127–132. 10.1016/S0378-1097(04)00040-0 15033230

[B24] Johnson J. R. Johnston B. Clabots C. R. Kuskowski M. A. Roberts E. DebRoy C. (2008). Virulence genotypes and phylogenetic background of *Escherichia coli* serogroup O6 isolates from humans, dogs, and cats. *J. Clin. Microbiol.* 46 417–422. 10.1128/JCM.00674-07 18003805 PMC2238128

[B25] Johnson J. R. Owens K. L. Clabots C. R. Weissman S. J. Cannon S. B. (2006). Phylogenetic relationships among clonal groups of extraintestinal pathogenic *Escherichia coli* as assessed by multi-locus sequence analysis. *Microbes Infect.* 8 1702–1713. 10.1016/j.micinf.2006.02.007 16820314

[B26] Kanehisa M. Goto S. (2000). KEGG: kyoto encyclopedia of genes and genomes. *Nucleic Acids Res.* 28 27–30. 10.1093/nar/28.1.27 10592173 PMC102409

[B27] Kline K. A. Falker S. Dahlberg S. Normark S. Henriques-Normark B. (2009). Bacterial adhesins in host-microbe interactions. *Cell Host Microbe* 5 580–592. 10.1016/j.chom.2009.05.011 19527885

[B28] Kohling H. L. Bittner A. Muller K. D. Buer J. Becker M. Rubben H. (2012). Direct identification of bacteria in urine samples by matrix-assisted laser desorption/ionization time-of-flight mass spectrometry and relevance of defensins as interfering factors. *J. Med. Microbiol.* 61 339–344. 10.1099/jmm.0.032284-0 22275503

[B29] Kucheria R. Dasgupta P. Sacks S. H. Khan M. S. Sheerin N. S. (2005). Urinary tract infections: new insights into a common problem. *Postgrad. Med. J.* 81 83–86. 10.1136/pgmj.2004.023036 15701738 PMC1743204

[B30] Larkin M. A. Blackshields G. Brown N. P. Chenna R. McGettigan P. A. McWilliam H. (2007). Clustal W and Clustal X version 2.0. *Bioinformatics.* 23 2947–2948. 10.1093/bioinformatics/btm404 17846036

[B31] Leusch H. G. Drzeniek Z. Markos-Pusztai Z. Wagener C. (1991). Binding of *Escherichia coli* and *Salmonella* strains to members of the carcinoembryonic antigen family: differential binding inhibition by aromatic alpha-glycosides of mannose. *Infect. Immun.* 59 2051–2057. 1674739 10.1128/iai.59.6.2051-2057.1991 PMC257964

[B32] Li R. Zhu H. Ruan J. Qian W. Fang X. Shi Z. (2010). De novo assembly of human genomes with massively parallel short read sequencing. *Genome Res.* 20 265–272. 10.1101/gr.097261.109 20019144 PMC2813482

[B33] Litwin M. S. Saigal C. S. (2007). *Urologic Diseases in America.* Washington, DC: Government Printing Office.

[B34] Manges A. R. Johnson J. R. Foxman B. O’Bryan T. T. Fullerton K. E. Riley L. W. (2001). Widespread distribution of urinary tract infections caused by a multidrug-resistant *Escherichia coli* clonal group. *N. Engl. J. Med.* 345 1007–1013. 10.1056/NEJMoa011265 11586952

[B35] Muller J. Szklarczyk D. Julien P. Letunic I. Roth A. Kuhn M. (2010). eggNOG v2.0: extending the evolutionary genealogy of genes with enhanced non-supervised orthologous groups, species and functional annotations. *Nucleic Acids Res.* 38 D190–D195. 10.1093/nar/gkp951 19900971 PMC2808932

[B36] Nielubowicz G. R. Mobley H. L. (2010). Host-pathogen interactions in urinary tract infection. *Nat. Rev. Urol.* 7 430–441. 10.1038/nrurol.2010.101 20647992

[B37] Pallen M. J. Loman N. J. Penn C. W. (2010). High-throughput sequencing and clinical microbiology: progress, opportunities and challenges. *Curr. Opin. Microbiol.* 13 625–631. 10.1016/j.mib.2010.08.003 20843733

[B38] Proft T. Baker E. N. (2009). Pili in Gram-negative and Gram-positive bacteria - structure, assembly and their role in disease. *Cell Mol. Life Sci.* 66 613–635. 10.1007/s00018-008-8477-4 18953686 PMC11131518

[B39] Rasko D. A. Rosovitz M. J. Myers G. S. Mongodin E. F. Fricke W. F. Gajer P. (2008). The pangenome structure of *Escherichia coli*: comparative genomic analysis of *E. coli* commensal and pathogenic isolates. *J. Bacteriol.* 190 6881–6893. 10.1128/JB.00619-08 18676672 PMC2566221

[B40] Reeves P. R. Liu B. Zhou Z. Li D. Guo D. Ren Y. (2011). Rates of mutation and host transmission for an *Escherichia coli* clone over 3 years. *PLoS ONE* 6:e26907 10.1371/journal.pone.0026907 PMC3203180 22046404

[B41] Rogers B. A. Ingram P. R. Runnegar N. Pitman M. C. Freeman J. T. Athan E. (2015). Sequence type 131 fimH30 and fimH41 subclones amongst *Escherichia coli* isolates in Australia and New Zealand. *Int. J. Antimicrob. Agents* 45 351–358. 10.1016/j.ijantimicag.2014.11.015 25707371

[B42] Sauer F. G. Remaut H. Hultgren S. J. Waksman G. (2004). Fiber assembly by the chaperone-usher pathway. *Biochim. Biophys. Acta* 1694 259–267. 10.1016/j.bbamcr.2004.02.010 15546670

[B43] Scholes D. Hooton T. M. Roberts P. L. Gupta K. Stapleton A. E. Stamm W. E. (2005). Risk factors associated with acute pyelonephritis in healthy women. *Ann. Intern. Med.* 142 20–27. 10.7326/0003-4819-142-1-200501040-00008 15630106 PMC3722605

[B44] Shaikh N. Morone N. E. Bost J. E. Farrell M. H. (2008). Prevalence of urinary tract infection in childhood: a meta-analysis. *Pediatr. Infect. Dis. J.* 27 302–308. 10.1097/INF.0b013e31815e4122 18316994

[B45] Tamura K. Peterson D. Peterson N. Stecher G. Nei M. Kumar S. (2011). MEGA5: molecular evolutionary genetics analysis using maximum likelihood, evolutionary distance, and maximum parsimony methods. *Mol. Biol. Evol.* 28 2731–2739. 10.1093/molbev/msr121 21546353 PMC3203626

[B46] Tartof S. Y. Solberg O. D. Manges A. R. Riley L. W. (2005). Analysis of a uropathogenic *Escherichia coli* clonal group by multilocus sequence typing. *J. Clin. Microbiol.* 43 5860–5864. 10.1128/JCM.43.12.5860-5864.2005 16333067 PMC1317175

[B47] Totsika M. Beatson S. A. Sarkar S. Phan M. D. Petty N. K. Bachmann N. (2011). Insights into a multidrug resistant *Escherichia coli* pathogen of the globally disseminated ST131 lineage: genome analysis and virulence mechanisms. *PLoS ONE* 6:e26578 10.1371/journal.pone.0026578 PMC3203889 22053197

[B48] Touchon M. Hoede C. Tenaillon O. Barbe V. Baeriswyl S. Bidet P. (2009). Organised genome dynamics in the *Escherichia coli* species results in highly diverse adaptive paths. *PLoS Genet.* 5:e1000344 10.1371/journal.pgen.1000344 PMC2617782 19165319

[B49] Ulett G. C. Totsika M. Schaale K. Carey A. J. Sweet M. J. Schembri M. A. (2013). Uropathogenic *Escherichia coli* virulence and innate immune responses during urinary tract infection. *Curr. Opin. Microbiol.* 16 100–107. 10.1016/j.mib.2013.01.005 23403118

[B50] Weissman S. J. Beskhlebnaya V. Chesnokova V. Chattopadhyay S. Stamm W. E. Hooton T. M. (2007). Differential stability and trade-off effects of pathoadaptive mutations in the *Escherichia coli* FimH adhesin. *Infect. Immun.* 75 3548–3555. 10.1128/IAI.01963-06 17502398 PMC1932922

[B51] Weissman S. J. Chattopadhyay S. Aprikian P. Obata-Yasuoka M. Yarova-Yarovaya Y. Stapleton A. (2006). Clonal analysis reveals high rate of structural mutations in fimbrial adhesins of extraintestinal pathogenic *Escherichia coli*. *Mol. Microbiol.* 59 975–988. 10.1111/j.1365-2958.2005.04985.x 16420365 PMC1380272

[B52] Weissman S. J. Johnson J. R. Tchesnokova V. Billig M. Dykhuizen D. Riddell K. (2012). High-resolution two-locus clonal typing of extraintestinal pathogenic *Escherichia coli*. *Appl. Environ. Microbiol.* 78 1353–1360. 10.1128/AEM.06663-11 22226951 PMC3294456

[B53] Welch R. A. Burland V. Plunkett G. III Redford P. Roesch P. Rasko D. (2002). Extensive mosaic structure revealed by the complete genome sequence of uropathogenic *Escherichia coli*. *Proc. Natl. Acad. Sci. U.S.A.* 99 17020–17024. 10.1073/pnas.252529799 12471157 PMC139262

[B54] Wurpel D. J. Beatson S. A. Totsika M. Petty N. K. Schembri M. A. (2013). Chaperone-usher fimbriae of *Escherichia coli. PLoS ONE* 8:e52835. 10.1371/journal.pone.0052835 PMC3559732 23382825

[B55] Zdziarski J. Brzuszkiewicz E. Wullt B. Liesegang H. Biran D. Voigt B. (2010). Host imprints on bacterial genomes–rapid, divergent evolution in individual patients. *PLoS Pathog.* 6:e1001078 10.1371/journal.ppat.1001078 PMC2928814 20865122

[B56] Zorc J. J. Kiddoo D. A. Shaw K. N. (2005). Diagnosis and management of pediatric urinary tract infections. *Clin. Microbiol. Rev.* 18 417–422. 10.1128/CMR.18.2.417-422.2005 15831830 PMC1082801

