# Supplementary material for: A High-resolution Typing Assay for Uropathogenic Escherichia coli Based on Fimbrial Diversity
Source: Front Microbiol. 2016 Apr 29;7:623. doi: 10.3389/fmicb.2016.00623 (PMC4850163; doi:10.3389/fmicb.2016.00623)
Supplement: Supplementary file 1 [file Table_1.PDF]

Table S1. UPEC strains with published genomes and clinical UPEC strains used in this study

Strain	Accession number	Sequence type	Type of infection	Reference or source
UPEC strains with published genomes				
CFT073	NC_004431	ST73	Pyelonephritis	(Welch et al., 2002)
Di2	NC_017651	ST73	UTI	(Reeves et al., 2011)
Di14	NC_017652	ST73	UTI	(Reeves et al., 2011)
536	NC_008253	ST127	Pyelonephritis	(Hochhut et al., 2006)
F11	AAJU02000000	ST127	Cystitis	(Rasko et al., 2008)
EC958	NZ_HG941718	ST131	UTI	(Totsika et al., 2011)
UTI89	NC_007946	ST95	Acute bladder infection	(Chen et al., 2006)
UMN026	NC_011751	ST597	Cystitis	(Touchon et al., 2009)
IAI39	NC_011750	ST62	Pyelonephritis	(Touchon et al., 2009)
ABU83972	NC_017631	ST73	Asymptomatic bacteriuria	(Zdziarski et al., 2010)
NA114	NC_017644	ST131	UTI	(Avasthi et al., 2011)
upec-100	GCA_000779215	ST372	UTI	(Salipante et al., 2015)
upec-101	GCA_000779055	ST420	UTI	(Salipante et al., 2015)
upec-103	GCA_000781455	ST3018	UTI	(Salipante et al., 2015)
upec-104	GCA_000781435	ST129	UTI	(Salipante et al., 2015)
upec-105	GCA_000781415	ST70	UTI	(Salipante et al., 2015)
upec-111	GCA_000781275	ST2970	UTI	(Salipante et al., 2015)
upec-112	GCA_000781295	ST646	UTI	(Salipante et al., 2015)
upec-115	GCA_000781215	ST1072	UTI	(Salipante et al., 2015)
upec-116	GCA_000781195	ST681	UTI	(Salipante et al., 2015)
upec-119	GCA_000781145	ST210	UTI	(Salipante et al., 2015)
upec-121	GCA_000781075	ST297	UTI	(Salipante et al., 2015)
upec-127	GCA_000780965	ST10	UTI	(Salipante et al., 2015)
upec-130	GCA_000780895	ST101	UTI	(Salipante et al., 2015)
upec-132	GCA_000780845	ST393	UTI	(Salipante et al., 2015)
upec-133	GCA_000780835	ST38	UTI	(Salipante et al., 2015)
upec-137	GCA_000780655	ST354	UTI	(Salipante et al., 2015)
upec-146	GCA_000780555	ST88	UTI	(Salipante et al., 2015)
upec-149	GCA_000780475	ST1193	UTI	(Salipante et al., 2015)
upec-150	GCA_000780415	ST491	UTI	(Salipante et al., 2015)
upec-153	GCA_000780335	ST80	UTI	(Salipante et al., 2015)
upec-175	GCA_000779915	ST130	UTI	(Salipante et al., 2015)
upec-179	GCA_000779835	ST144	UTI	(Salipante et al., 2015)
upec-180	GCA_000779815	ST929	UTI	(Salipante et al., 2015)
upec-195	GCA_000779515	ST537	UTI	(Salipante et al., 2015)
upec-200	GCA_000779335	ST543	UTI	(Salipante et al., 2015)
upec-202	GCA_000779295	ST28	UTI	(Salipante et al., 2015)
upec-205	GCA_000778545	ST224	UTI	(Salipante et al., 2015)
upec-211	GCA_000778585	ST405	UTI	(Salipante et al., 2015)

upec-213	GCA_000778785	ST3752	UTI	(Salipante et al., 2015)
upec-219	GCA_000778725	ST91	UTI	(Salipante et al., 2015)
upec-233	GCA_000778395	ST421	UTI	(Salipante et al., 2015)
upec-236	GCA_000778355	ST2619	UTI	(Salipante et al., 2015)
upec-238	GCA_000778315	ST1249	UTI	(Salipante et al., 2015)
upec-243	GCA_000778235	ST1946	UTI	(Salipante et al., 2015)
upec-258	GCA_000777975	ST404	UTI	(Salipante et al., 2015)
upec-266	GCA_000777815	ST1159	UTI	(Salipante et al., 2015)
upec-274	GCA_000777655	ST3672	UTI	(Salipante et al., 2015)
upec-31	GCA_000777325	ST1844	UTI	(Salipante et al., 2015)
upec-33	GCA_000777285	ST906	UTI	(Salipante et al., 2015)
upec-38	GCA_000777195	ST555	UTI	(Salipante et al., 2015)
upec-58	GCA_000776115	ST83	UTI	(Salipante et al., 2015)
upec-59	GCA_000776675	ST569	UTI	(Salipante et al., 2015)
upec-78	GCA_000776065	ST3693	UTI	(Salipante et al., 2015)
upec-79	GCA_000776415	ST968	UTI	(Salipante et al., 2015)
Clinical UPEC strains				
1	JSVR00000000	ST12	UTI	Korczak Hospital
3	JSVP00000000	ST59	UTI	Korczak Hospital
4	JSVO00000000	ST648	UTI	Kopernik Hospital
5	JSVM00000000	ST135	UTI	Kopernik Hospital
7	JSVL00000000	ST442	UTI	Kopernik Hospital
8	JSVN00000000	ST12	UTI	Korczak Hospital
11	JSVK00000000	ST1858	UTI	Korczak Hospital
14	JSVQ00000000	ST361	UTI	Korczak Hospital
47	-	ST95	UTI	Diag-Med Non-public Healthcare Centre, Łódź
49	-	ST73	UTI	Medicover Medical Centre, Łódź
53	-	ST73	UTI	Independent Public Healthcare Centre, Łódź Bałuty
54	-	ST215	UTI	Kopernik Hospital
55	-	ST10	UTI	Independent Public Healthcare Centre, Łódź Bałuty
57	-	ST14	UTI	Jonscher Hospital
58	-	ST12	UTI	DAR Non-public Healthcare Centre, Wieruszów
59	-	ST14	UTI	Primary Healthcare Centre
60	-	ST79	UTI	Independent Public Healthcare Centre, Łódź Bałuty
62	-	ST354	UTI	Konstantynów
64	-	ST58	UTI	-
65	-	ST453	UTI	Independent Public Healthcare Centre, Łódź Bałuty
66	-	ST131	UTI	Kopernik Hospital

67	-	ST405	UTI	Cero-Med Public Medical Centre, Łódź
68	-	ST3352	UTI	Medicover Medical Centre, Łódź
69	-	ST705	UTI	Beja-Med Non-public Healthcare Centre, Łódź
70	-	ST127	UTI	Korczak Hospital, Neonatal Ward
71	-	ST12	UTI	Eskulap Public Medical Centre, Łódź
72	-	ST3439	UTI	Saint Jan Boży Bonifratres Hospital
74	-	ST95	UTI	Independent Public Healthcare Centre, Aleksandrów
75	-	ST117	UTI	Jonscher Hospital
76	-	ST88	UTI	Kopernik Hospital, Neurology Ward
77	-	ST14	UTI	Med-kol Non-public Healthcare Centre, Koluszki
78	-	ST5519	UTI	Independent Public Healthcare Centre, Aleksandrów
79	-	ST1431	UTI	Independent Public Healthcare Centre, Łódź Bałuty
80	-	ST215	UTI	Kopernik Hospital
81	-	ST295	UTI	Independent Public Healthcare Centre, Łódź Bałuty
82	-	ST224	UTI	Independent Public Healthcare Centre, Łódź Bałuty
83	-	ST5528	UTI	Kopernik Hospital
84	-	ST69	UTI	Diag-Med Non-public Healthcare Centre, Łódź
85	-	ST95	UTI	Independent Public Healthcare Centre, Tuszyn
86	-	ST69	UTI	Korczak Hospital
87	-	ST73	UTI	Łódź Śródmieście
88	-	ST359	UTI	Saint Jan Boży Bonifratres Hospital
89	-	ST10	UTI	Municipal Clinic, Łódź Widzew
90	-	ST14	UTI	Independent Public Healthcare Centre, Łódź Bałuty
91	-	ST73	UTI	Kopernik Hospital, Neurosurgery Ward
92	-	ST69	UTI	Municipal Clinic, Łódź Widzew
93	-	ST410	UTI	Independent Public Healthcare Centre, Łódź Bałuty
94	-	ST1618	UTI	Diag-Med Non-public Healthcare Centre, Łódź
95	-	ST73	UTI	Municipal Clinic, Łódź Widzew
96	-	unknown	UTI	Independent Public Healthcare Centre, Łódź Bałuty

97	-	ST537	UTI	-
98	-	ST2279	UTI	DAR Non-public Healthcare Centre, Wieruszów
99	-	ST191	UTI	Kopernik Hospital, Neurology Ward
100	-	unknown	UTI	Independent Public Healthcare Centre, Łódź Bałuty
101	-	unknown	UTI	Renoma Public Medical Centre, Łódź
102	-	ST405	UTI	Saint Jan Boży Bonifratres Hospital
103	-	ST69	UTI	Independent Public Healthcare Centre, Łódź Bałuty
104	-	ST69	UTI	Głowno
105	-	ST131	UTI	Jonscher Hospital
107	-	ST141	UTI	Municipal Clinic, Łódź Widzew
108	-	ST131	UTI	Laboratory of the Medical Analyses, Pabianice
109	-	ST95	UTI	Kopernik Hospital
110	-	ST131	UTI	Aditus Non-public Medical Laboratory, Łódź
111	-	ST69	UTI	Konstantynów
112	-	ST95	UTI	Diag-Med Non-public Healthcare Centre, Łódź
113	-	ST405	UTI	Medicover Medical Centre, Łódź
114	-	ST73	UTI	Independent Public Healthcare Centre, Łódź Bałuty
115	-	ST127	UTI	Aditus Non-public Medical Laboratory, Łódź
118	-	ST69	UTI	Kopernik Hospital
119	-	ST12	UTI	Independent Public Healthcare Centre, Łódź Bałuty
120	-	ST1394	UTI	Kopernik Hospital, Rheumatology Ward
121	-	ST58	UTI	Primary Healthcare Centre
122	-	ST1858	UTI	Saint Jan Boży Bonifratres Hospital
123	-	ST398	UTI	Aditus Non-public Medical Laboratory, Łódź
124	-	ST62	UTI	Independent Public Healthcare Centre, Łódź Bałuty

Reference

Avasthi, T. S., Kumar, N., Baddam, R., Hussain, A., Nandanwar, N., Jadhav, S., and Ahmed, N. (2011). Genome of multidrug-resistant uropathogenic *Escherichia coli* strain NA114 from India. *J Bacteriol.* 193, 4272-4273. doi: 10.1128/JB.05413-11

Chen, S. L., Hung, C. S., Xu, J., Reigstad, C. S., Magrini, V., Sabo, A. et al. (2006). Identification of genes subject to positive selection in uropathogenic strains of *Escherichia coli*: a comparative genomics approach. *Proc Natl Acad Sci U S A.* 103, 5977-5982. doi: 10.1073/pnas.0600938103

Hochhut, B., Wilde, C., Balling, G., Middendorf, B., Dobrindt, U., Brzuszkiewicz, E. et al. (2006). Role of pathogenicity island-associated integrases in the genome plasticity of uropathogenic *Escherichia coli* strain 536. *Mol Microbiol.* 61, 584-595. doi: 10.1111/j.1365-2958.2006.05255.x

Rasko, D. A., Rosovitz, M. J., Myers, G. S., Mongodin, E. F., Fricke, W. F., Gajer, P. et al. (2008). The pangenome structure of *Escherichia coli*: comparative genomic analysis of *E. coli* commensal and pathogenic isolates. *J Bacteriol.* 190, 6881-6893. doi: 10.1128/JB.00619-08

Reeves, P. R., Liu, B., Zhou, Z., Li, D., Guo, D., Ren, Y. et al. (2011). Rates of mutation and host transmission for an *Escherichia coli* clone over 3 years. *PLoS One.* 6, e26907. doi: 10.1371/journal.pone.0026907

Salipante, S. J., Roach, D. J., Kitzman, J. O., Snyder, M. W., Stackhouse, B., Butler-Wu, S. M. et al. (2015). Large-scale genomic sequencing of extraintestinal pathogenic *Escherichia coli* strains. *Genome Res.* 25, 119-128. doi: 10.1101/gr.180190.114

Totsika, M., Beatson, S. A., Sarkar, S., Phan, M. D., Petty, N. K., Bachmann, N. et al. (2011). Insights into a multidrug resistant *Escherichia coli* pathogen of the globally disseminated ST131 lineage: genome analysis and virulence mechanisms. *PLoS One.* 6, e26578. doi: 10.1371/journal.pone.0026578

Touchon, M., Hoede, C., Tenaillon, O., Barbe, V., Baeriswyl, S., Bidet, P. et al. (2009). Organised genome dynamics in the *Escherichia coli* species results in highly diverse adaptive paths. *PLoS Genet.* 5, e1000344. doi: 10.1371/journal.pgen.1000344

Welch, R. A., Burland, V., Plunkett, G., 3rd, Redford, P., Roesch, P., Rasko, D. et al. (2002). Extensive mosaic structure revealed by the complete genome sequence of uropathogenic *Escherichia coli*. *Proc Natl Acad Sci U S A.* 99, 17020-17024. doi: 10.1073/pnas.252529799

Zdziarski, J., Brzuszkiewicz, E., Wullt, B., Liesegang, H., Biran, D., Voigt, B. et al. (2010). Host imprints on bacterial genomes--rapid, divergent evolution in individual patients. *PLoS Pathog.* 6, e1001078. doi: 10.1371/journal.ppat.1001078
