# Supplementary material for: A High-resolution Typing Assay for Uropathogenic Escherichia coli Based on Fimbrial Diversity
Source: Front Microbiol. 2016 Apr 29;7:623. doi: 10.3389/fmicb.2016.00623 (PMC4850163; doi:10.3389/fmicb.2016.00623)
Supplement: Supplementary file 2 [file Table_2.PDF]

Table S2. Summary of genome sequences for 8 UPEC strains.

Strain No.	Total read	Total bases (bp)	Gene No.	Sequence total length (bp)	GC content (%)	Sequence type
1	4269420	1048661960	4893	6035566	51.5	ST12
3	3803049	931979422	4957	6027660	51.7	ST59
4	3712149	916333272	4840	5929342	51.6	ST648
5	4112998	1023941345	4437	5495086	51.8	ST135
7	3863867	964457049	4556	5783815	51.7	ST442
8	3789049	928023179	4807	5880825	51.4	ST12
11	5027255	1251141393	4636	5816744	51.6	ST1858
14	4594042	1139623880	4274	5324903	52.0	ST361
