# Supplementary material for: A High-resolution Typing Assay for Uropathogenic Escherichia coli Based on Fimbrial Diversity
Source: Front Microbiol. 2016 Apr 29;7:623. doi: 10.3389/fmicb.2016.00623 (PMC4850163; doi:10.3389/fmicb.2016.00623)
Supplement: Supplementary file 3 [file Table_3.PDF]

Table S3. Nucleotide polymorphism of MLST genes and chaperone-usher fimbriae genes in UPEC strains.

Gene /fimbriae type	Gene	Average π value	Average π value of genes in the type
MLST	*adk*	0.012±0.007	0.012±0.007
	fumC	0.026±0.013	
	gyrB	0.011±0.006	
	icd	0.011±0.006	
	mdh	0.007±0.004	
	purA	0.008±0.005	
	recA	0.009±0.009	
Mat	*matB*	0.008±0.004	0.010±0.004
	yagY	0.009±0.004	
	yagX	0.012±0.007	
	yagW	0.008±0.005	
	yagV	0.016±0.008	
Type 1	*fimA*	0.090±0.044	0.025±0.029
	fimI	0.012±0.003	
	fimC	0.011±0.005	
	fimD	0.012±0.007	
	fimF	0.020±0.006	
	fimG	0.009±0.004	
	fimH	0.018±0.009	
F9	*c1935*	0.010±0.005	0.013±0.005
	c1934	0.015±0.008	
	ydeS	0.015±0.005	
	ydeR	0.008±0.001	
	ydeQ	0.020±0.007	
Yad	*yadN*	0.11±0.023	0.132±0.046
	ecpD	0.096±0.023	
	htrE	0.115±0.014	
	yadM	0.184±0.031	
	yadL	0.119±0.021	
	yadK	0.128±0.011	
	yadC	0.224±0.049	
Yeh	*yehD*	0.020±0.009	0.017±0.004
	yehC	0.020±0.011	
	yehB	0.015±0.007	
	yehA	0.012±0.005	
Yfc	*yfcV*	0.105±0.027	0.178±0.104
	yfcU	0.090±0.016	
	yfcS	0.096±0.018	
	yfcR	0.204±0.022	
	yfcQ	0.198±0.017	

	yfcP	0.167±0.027	
	yfcO	0.386±0.076	
