# Supplementary material for: A High-resolution Typing Assay for Uropathogenic Escherichia coli Based on Fimbrial Diversity
Source: Front Microbiol. 2016 Apr 29;7:623. doi: 10.3389/fmicb.2016.00623 (PMC4850163; doi:10.3389/fmicb.2016.00623)
Supplement: Supplementary file 4 [file Table_4.PDF]

Table S4. Primers for typing used in this study

Targeted gene/fimbriae type*	Sequence (5’ - 3’)
yagV	AAGTCAGCGCTTCAGGAG/TGACGATCGCTTCCACATC
fimF	ATCAACTGCCACCAGAGAG/CGGACAATTAGTCAACTCAAG
fimH	CGTTACAGGTCAGAGCATTG/CGCGTCTTATCTGGCCTAC
CS1-like	GCTTGTACAACCGACAACA/CTCTGTTCATCCTGTTCAGA
Mat	ATGGACAGTTACGCATCC/TCCACATCGTAAATACCGTA
Type 1	ATGCCGCAGGTAATAGTG/GAATTGCTCATCGACATTAC
F1C/S	CACCTGCACATCACTGAAT/GTGAATTGTCGGTGCATAC
F9	CGACACTTGCAGATGACAC/TGACATACTGTAACTGGCGT
Ycb	GTTGAGATAACGCCAGAGA/CACTCGACGACGTAGAGTAG
Auf	TGACTTATCTTCCTGGTAGC/GCTCCAGTTTACCTGCTG
Sfm	ATTAGAGAATGGCACATCC/ATCGCCATTTGAAGATGT
LPF	AATAGTTACGCCACCTATTC/TGAAGAGTACGCGATAGC
ECSF-0165	CTCCGTGAGTTCGGTCTT/AACAGGTGTCTCAGCATGAT
ECSF-4008	CTGATGGTGATAATGCCA/ACTGAGGCTCAGACACACTA
CS12	ATGTCTCGCGTCAATGTC/CAGCATCGTAATAGTGTTCA
AFA	GTACCTGAAGTACAACGTCAC/CAGGACGTACTGTATGACG
Yad	GAAGATGGTCAAACCGAC/GGTGCTGTTTCCGTAGTTA
Yeh	CAGGTCGTAGCCATATTGA/TGATTCTCGTCATAAGCATG
Yeh-like	CTGCCTAAGGTGCTACTAAC/TGCTGACATCGAGATCAGA
F17-lik	GTCATGGTAACCCTGTGC/GCAAGGTCATGCATTATACT
Yfc	TCGCAACATGAGCATCTC/GTAGCTACCGTCACGCAA
P	CTCAGTCGTCAGGAGATAAG/ACCATCGAGTCGTACAGC
Pix	GCTGTACACCGTCACACTC/TATCAGACATCCGCAACA
Yqi	CCGCAACATCTCCTACAG/CGCGCTTTCACTAATGTT
Ybg	ACCAAATCAGTAACGGACA/CCTGACTGTTCATGGTTATC

* The primers for fimbriae type identification are based on sequences of usher protein encoding gene.
