# Supplementary material for: A High-resolution Typing Assay for Uropathogenic Escherichia coli Based on Fimbrial Diversity
Source: Front Microbiol. 2016 Apr 29;7:623. doi: 10.3389/fmicb.2016.00623 (PMC4850163; doi:10.3389/fmicb.2016.00623)
Supplement: Supplementary file 5 [file Table_5.PDF]

**Table S5.** SNPs in the combined four genes (yagV, fimF/H and fumC) of 130 strains

| | | 6 | 7 | 39 | 42 | 48 | 54 | 90 | 110 | 111 | 127 | 141 | 151 | 166 | 171 | 189 | 192 | 203 | 210 | 219 | 223 | 234 | 261 | 266 | 291 | 300 | 306 | 309 | 312 | 318 | 323 | 324 | 329 | 333 | 339 | 341 | 348 | 350 | 354 | 360 | 363 | 378 | 383 | 403 | 418 | 419 | 425 | 432 | 433 | 439 | 440 | 441 | 445 | 447 | 452 | 459 | 468 | 477 | 489 | 502 | 517 | 522 | 531 | 546 | 549 | 550 | 554 |
|---|---|---|---|---|---|---|---|---|---|---|---|---|---|---|---|---|---|---|---|---|---|---|---|---|---|---|---|---|---|---|---|---|---|---|---|---|---|---|---|---|---|---|---|---|---|---|---|---|---|---|---|---|---|---|---|---|---|---|---|---|---|---|---|---|---|---|---|
| 1 | 53 | G | G | C | T | T | G | A | C | A | G | T | G | C | C | G | G | C | G | G | G | C | T | A | G | T | A | C | C | T | A | G | G | T | A | G | C | A | C | G | G | G | C | A | A | A | C | C | A | A | C | A | A | G | G | T | A | G | G | T | G | T | T | C | G | G | A |
| 2 | Di2 | G | G | C | T | T | A | C | C | A | G | T | G | C | C | A | A | C | G | G | G | C | T | G | G | T | G | C | C | T | T | G | A | C | A | G | T | A | C | G | G | G | C | A | A | A | C | T | C | A | C | C | A | G | G | C | G | G | A | T | G | T | T | C | G | G | A |
| | Di14 | G | G | C | T | T | A | C | C | A | G | T | G | C | C | A | A | C | G | G | G | C | T | G | G | T | G | C | C | T | T | G | A | C | A | G | T | A | C | G | G | G | C | A | A | A | C | T | C | A | C | C | A | G | G | C | G | G | A | T | G | T | T | C | G | G | A |
| | CFT073 | G | G | C | T | T | A | C | C | A | G | T | G | C | C | A | A | C | G | G | G | C | T | G | G | T | G | C | C | T | T | G | A | C | A | G | T | A | C | G | G | G | C | A | A | A | C | T | C | A | C | C | A | G | G | C | G | G | A | T | G | T | T | C | G | G | A |
| 3 | 49 | G | G | C | T | T | A | C | C | A | G | T | G | C | C | A | A | C | G | G | G | C | T | G | G | T | G | C | C | T | T | G | A | C | A | G | T | A | C | G | G | G | C | A | A | A | C | T | C | A | C | C | A | G | G | C | G | G | A | T | G | T | T | C | G | G | A |
| | 87 | G | G | C | T | T | A | C | C | A | G | T | G | C | C | A | A | C | G | G | G | C | T | G | G | T | G | C | C | T | T | G | A | C | A | G | T | A | C | G | G | G | C | A | A | A | C | T | C | A | C | C | A | G | G | C | G | G | A | T | G | T | T | C | G | G | A |
| | 91 | G | G | C | T | T | A | C | C | A | G | T | G | C | C | A | A | C | G | G | G | C | T | G | G | T | G | C | C | T | T | G | A | C | A | G | T | A | C | G | G | G | C | A | A | A | C | T | C | A | C | C | A | G | G | C | G | G | A | T | G | T | T | C | G | G | A |
| | 95 | G | G | C | T | T | A | C | C | A | G | T | G | C | C | A | A | C | G | G | G | C | T | G | G | T | G | C | C | T | T | G | A | C | A | G | T | A | C | G | G | G | C | A | A | A | C | T | C | A | C | C | A | G | G | C | G | G | A | T | G | T | T | C | G | G | A |
| | 72 | G | G | C | T | T | A | C | C | A | G | T | G | C | C | A | A | C | G | G | G | C | T | G | G | T | G | C | C | T | T | G | A | C | A | G | T | A | C | G | G | G | C | A | A | A | C | T | C | A | C | C | A | G | G | C | G | G | A | T | G | T | T | C | G | G | A |
| 4 | UPEC-79 | G | G | C | T | T | A | C | C | A | G | T | G | C | C | A | A | C | G | G | G | C | T | G | G | T | G | C | C | T | T | G | A | C | A | G | T | A | C | G | G | G | C | A | A | A | C | T | C | A | C | C | A | G | G | C | G | G | A | T | G | T | T | C | G | G | A |
| 5 | 114 | G | G | C | T | T | A | C | C | A | G | T | G | C | C | A | A | C | G | G | G | C | T | G | G | T | G | C | C | T | T | G | A | C | A | G | T | A | C | G | G | G | C | A | A | A | C | T | C | A | C | C | A | G | G | C | G | G | A | T | G | T | T | C | G | G | A |
| 6 | ABU83972 | G | G | C | T | T | A | C | C | A | G | T | G | C | C | A | A | C | G | G | G | C | T | G | G | T | G | C | C | T | T | G | A | C | A | G | T | A | C | G | G | G | C | A | A | A | C | T | C | A | C | C | A | G | G | C | G | G | A | T | G | T | T | C | G | G | A |
| 7 | 94 | G | G | C | T | T | A | C | C | A | G | T | G | C | C | A | A | C | G | G | G | C | T | G | G | T | G | C | C | T | T | G | A | C | A | G | T | A | C | G | G | G | C | A | A | A | C | T | C | A | C | C | A | G | G | C | G | G | A | T | G | T | T | C | G | G | A |
| 8 | UPEC-116 | G | G | C | T | T | A | C | C | G | G | T | G | A | C | A | A | C | G | G | A | C | T | G | G | T | G | C | C | T | T | G | A | C | A | C | T | A | C | G | G | G | C | A | A | A | T | T | C | A | C | C | A | G | G | C | G | G | A | A | G | T | T | C | G | G | A |
| 9 | UPEC-180 | G | G | C | T | T | A | C | C | A | G | T | G | C | C | A | A | C | G | G | G | C | T | G | G | T | G | C | C | T | T | A | A | C | A | C | T | A | C | G | G | G | C | A | A | A | T | T | C | A | C | C | A | G | G | C | G | G | A | A | G | T | T | C | G | G | A |
| 10 | UPEC-100 | G | G | C | T | T | A | C | C | A | G | T | G | C | C | A | A | C | G | G | G | C | T | G | G | T | G | C | C | T | T | G | A | C | A | G | T | A | C | G | G | G | C | A | A | A | C | T | C | A | C | C | A | G | G | C | G | G | A | T | G | T | T | C | G | G | A |
| 11 | UPEC-112 | G | G | C | T | T | A | C | C | A | G | T | G | C | C | A | A | C | G | G | G | C | T | G | G | T | G | C | C | T | T | G | A | C | A | G | T | A | C | G | G | G | G | A | A | A | C | T | C | A | C | C | A | G | G | C | G | G | A | T | G | T | T | C | G | G | A |
| 12 | 85 | G | G | C | T | T | A | C | C | G | G | T | G | C | C | A | A | C | G | G | G | C | C | G | G | T | G | C | C | T | T | G | A | C | A | G | T | A | C | G | G | G | C | A | A | A | C | T | C | A | C | C | A | G | G | C | G | G | A | T | G | T | T | C | G | G | A |
| | UPEC-115 | G | G | C | T | T | A | C | C | G | G | T | G | C | C | A | A | C | G | G | G | C | C | G | G | T | G | C | C | T | T | G | A | C | A | G | T | A | C | G | G | G | C | A | A | A | C | T | C | A | C | C | A | G | G | C | G | G | A | T | G | T | T | C | G | G | A |
| 13 | UTI89 | G | G | C | T | T | A | C | C | G | G | T | G | C | C | A | A | C | G | G | G | C | C | G | G | T | G | C | C | T | T | G | A | C | A | G | T | A | C | G | G | G | C | A | A | A | C | T | C | A | C | C | A | G | G | C | G | G | A | T | G | T | T | C | G | G | A |
| 14 | UPEC-153 | G | G | C | T | T | A | C | C | G | G | T | G | C | C | A | A | C | G | G | G | C | T | G | G | T | G | C | C | T | T | G | A | C | A | C | T | A | C | G | G | G | C | A | A | A | C | T | C | A | C | C | A | G | G | C | G | G | A | T | G | T | T | C | G | G | A |
| 15 | 101 | G | G | C | T | T | A | C | C | G | G | T | G | C | C | A | A | C | G | G | G | C | T | G | G | T | G | C | C | T | T | G | A | C | A | C | T | A | C | G | G | G | C | A | A | A | C | T | C | A | C | C | A | G | G | C | G | G | A | T | G | T | T | C | G | G | A |
| 16 | 122 | G | G | C | T | T | A | C | C | G | G | T | G | C | C | A | A | C | G | G | G | C | T | G | G | T | G | C | C | T | T | G | A | C | A | C | T | A | C | G | G | G | C | A | A | A | C | T | C | A | C | C | A | G | G | C | G | G | A | T | G | T | T | C | G | G | A |
| 17 | 11 | G | G | C | T | T | A | C | C | G | G | T | G | C | C | A | A | C | G | G | G | C | T | G | G | T | G | C | C | T | T | G | A | C | A | C | T | A | C | G | G | G | C | A | A | A | C | T | C | A | C | C | A | G | G | C | G | G | A | T | G | T | T | C | G | G | A |
| 18 | 97 | G | G | C | T | T | A | C | C | G | G | T | G | C | C | A | A | C | G | G | G | C | T | G | G | T | G | C | C | T | T | G | A | C | A | C | T | A | C | G | G | G | C | A | A | A | C | T | C | A | C | C | A | G | G | C | G | G | A | T | G | T | T | C | G | G | A |
| 19 | UPEC-195 | G | G | C | T | T | A | C | C | G | G | T | G | C | C | A | A | C | G | G | G | C | T | G | G | T | G | C | C | T | T | G | A | C | A | C | T | A | C | G | G | G | C | A | A | A | T | T | C | A | C | C | A | G | G | C | G | G | A | A | G | T | T | C | G | G | A |
| 20 | UPEC-150 | G | G | C | T | T | A | C | C | G | G | T | G | C | C | A | A | C | G | G | G | C | T | G | G | T | G | C | C | T | T | G | A | C | A | C | T | A | C | G | G | G | C | A | A | A | C | T | C | A | C | C | A | G | G | C | G | G | A | T | G | T | T | C | G | G | A |
| 21 | UPEC-101 | G | G | C | T | T | A | C | C | A | G | T | G | C | C | A | A | C | G | G | G | C | T | G | G | T | G | C | C | T | T | G | A | C | A | C | T | A | C | G | G | G | C | A | A | A | T | T | C | A | C | C | A | G | G | C | G | G | A | A | G | T | T | C | G | G | A |
| 22 | UPEC-59 | G | G | C | T | T | A | C | C | A | G | T | G | C | C | A | A | C | G | G | G | C | T | G | G | T | G | C | C | T | T | G | A | C | A | C | T | A | C | G | G | G | C | A | A | A | T | T | C | A | C | C | A | G | G | C | G | G | A | A | G | T | T | C | G | G | A |
| 23 | 119 | G | G | C | T | T | A | C | C | G | G | T | G | C | C | A | A | C | G | A | G | C | T | G | G | T | G | C | C | T | T | G | A | C | A | C | T | A | C | G | G | G | C | A | A | A | C | T | C | A | C | C | A | G | G | C | G | G | A | T | G | T | T | C | G | G | A |
| 24 | 8 | G | G | C | T | T | A | C | C | G | G | T | G | C | C | A | A | C | G | A | G | C | T | G | G | T | G | C | C | T | T | G | A | C | A | C | T | A | C | G | G | G | C | A | A | A | C | T | C | A | C | C | A | G | G | C | G | G | A | T | G | T | T | C | G | G | A |
| 25 | 1 | G | G | C | T | T | A | C | C | G | G | T | G | C | C | A | A | C | G | A | G | C | T | G | G | T | G | C | C | T | T | G | A | C | A | C | T | A | C | G | G | G | C | A | A | A | C | T | C | A | C | C | A | G | G | C | G | G | A | T | G | T | T | C | G | G | A |
| 26 | 58 | G | G | C | T | T | A | C | C | G | G | T | G | C | C | A | A | C | G | A | G | C | T | G | G | T | G | C | C | T | T | G | A | C | A | C | T | A | C | G | G | G | C | A | A | A | C | T | C | A | C | C | A | G | G | C | G | G | A | T | G | T | T | C | G | G | A |
| | 71 | G | G | C | T | T | A | C | C | G | G | T | G | C | C | A | A | C | G | A | G | C | T | G | G | T | G | C | C | T | T | G | A | C | A | C | T | A | C | G | G | G | C | A | A | A | C | T | C | A | C | C | A | G | G | C | G | G | A | T | G | T | T | C | G | G | A |
| 27 | 68 | G | G | C | T | T | A | C | C | G | G | T | G | C | C | A | A | C | G | A | G | C | T | G | G | T | G | C | C | T | T | G | A | C | A | C | T | A | C | G | G | G | C | A | A | A | C | T | C | A | C | C | A | G | G | C | G | G | A | T | G | T | T | C | G | G | A |
| 28 | 107 | G | G | C | T | T | A | C | C | G | G | T | G | C | C | A | A | C | G | G | G | C | T | G | G | T | G | C | C | T | T | G | A | C | A | C | T | A | C | G | G | G | C | A | A | A | C | T | C | A | C | C | A | G | G | C | G | G | A | T | G | T | T | C | G | G | A |
| 29 | UPEC-179 | G | G | C | T | T | A | C | C | G | G | T | G | C | C | A | A | C | G | G | G | C | C | G | G | T | G | C | C | T | T | G | A | C | A | G | T | A | C | G | G | G | C | A | A | A | C | T | C | A | C | C | A | G | G | C | G | G | A | T | G | T | T | C | G | G | A |
| 30 | UPEC-78 | G | G | C | T | T | A | C | C | G | G | T | G | C | C | A | A | C | G | G | G | C | T | G | G | T | G | C | C | T | T | G | A | C | A | G | T | A | C | G | G | G | C | A | A | A | C | T | C | A | C | C | A | G | G | C | G | G | A | T | G | T | T | C | G | G | A |
| 31 | UPEC-274 | G | G | C | T | T | A | C | C | A | G | T | G | C | C | A | A | C | G | G | G | C | T | G | G | T | G | T | C | T | T | G | A | C | A | C | T | A | C | G | G | G | C | A | A | A | C | T | C | A | C | C | A | G | G | C | G | G | A | T | G | T | T | C | G | G | A |
| 32 | UPEC-243 | G | G | C | T | T | A | C | C | A | G | T | G | C | C | A | A | C | G | G | G | C | T | G | G | T | G | C | C | T | T | G | A | C | A | G | T | A | C | G | G | G | C | A | A | A | C | T | C | A | C | C | A | G | G | C | G | G | A | T | G | T | T | C | G | G | A |

| | | | | | | | | | | | | | | | | | | | | | | | | | | | | | | | | | | | | | | | | | | | | | | | | | | | | | | | | | | | | | | | | | | | |
|---|---|---|---|---|---|---|---|---|---|---|---|---|---|---|---|---|---|---|---|---|---|---|---|---|---|---|---|---|---|---|---|---|---|---|---|---|---|---|---|---|---|---|---|---|---|---|---|---|---|---|---|---|---|---|---|---|---|---|---|---|---|---|---|---|---|---|---|
| 33 | 5 | G | G | C | T | T | A | C | C | A | G | T | G | C | C | A | A | C | G | G | G | C | T | G | G | T | G | C | C | T | T | G | A | C | A | G | T | A | C | G | G | G | C | A | A | A | C | T | C | A | C | C | A | G | G | C | G | G | A | T | G | T | T | C | G | G | A |
| 34 | 70 | G | G | C | T | T | A | C | C | A | G | T | G | C | C | A | A | C | G | G | G | C | T | G | G | T | G | C | C | T | T | G | A | C | A | G | T | A | C | G | G | G | C | A | A | A | C | T | C | A | C | C | A | G | G | C | G | G | A | T | G | T | T | C | G | G | A |
| | 115 | G | G | C | T | T | A | C | C | A | G | T | G | C | C | A | A | C | G | G | G | C | T | G | G | T | G | C | C | T | T | G | A | C | A | G | T | A | C | G | G | G | C | A | A | A | C | T | C | A | C | C | A | G | G | C | G | G | A | T | G | T | T | C | G | G | A |
| 35 | 536 | G | G | C | T | T | A | C | C | A | G | T | G | C | C | A | A | C | G | G | G | C | T | G | G | T | G | C | C | T | T | G | A | C | A | G | T | A | C | G | G | G | C | A | A | A | C | T | C | A | C | C | A | G | G | C | G | G | A | T | G | T | T | C | G | G | A |
| 36 | F11 | G | G | C | T | T | A | C | C | A | G | T | G | C | C | A | A | C | G | G | G | C | T | G | G | T | G | C | C | T | T | G | A | C | A | G | T | A | C | G | G | G | C | A | A | A | C | T | C | A | C | C | A | G | G | C | G | G | A | T | G | T | T | C | G | G | A |
| 37 | UPEC-38 | G | G | C | T | T | A | A | C | A | G | T | G | C | C | A | A | C | G | G | G | C | T | A | G | T | G | T | C | T | T | G | A | C | T | C | T | A | C | G | G | G | C | A | A | A | C | C | C | A | C | A | A | G | T | C | G | A | A | T | G | T | T | C | G | C | A |
| 38 | UPEC-58 | G | G | C | T | T | A | C | C | A | G | T | G | C | C | A | A | C | G | G | G | C | T | G | G | T | G | C | C | T | T | G | A | C | A | G | T | A | C | G | G | G | C | A | A | A | C | T | C | A | C | C | A | G | G | C | G | G | A | T | G | T | T | C | G | G | A |
| 39 | UPEC-219 | G | G | C | T | T | A | C | C | G | G | T | G | C | C | A | A | C | G | G | G | C | C | G | G | T | G | T | C | T | T | G | A | C | A | C | T | A | C | G | G | G | C | A | A | A | C | T | C | A | C | C | G | G | G | C | G | G | A | T | G | T | T | C | G | G | A |
| 40 | 69 | G | G | C | T | T | A | C | C | A | G | T | G | C | C | A | A | C | G | G | G | C | C | G | G | T | G | C | C | T | T | G | A | C | A | G | T | A | C | G | G | G | C | A | A | A | C | T | C | A | C | A | A | G | G | C | G | G | A | T | G | T | T | C | G | G | A |
| | 110 | G | G | C | T | T | A | C | C | A | G | T | G | C | C | A | A | C | G | G | G | C | C | G | G | T | G | C | C | T | T | G | A | C | A | G | T | A | C | G | G | G | C | A | A | A | C | T | C | A | C | A | A | G | G | C | G | G | A | T | G | T | T | C | G | G | A |
| 41 | UPEC-266 | G | G | C | T | T | A | C | C | G | G | T | G | C | C | A | A | C | G | G | G | C | T | G | G | T | G | C | C | T | T | G | A | C | A | C | T | A | C | G | G | G | C | A | A | A | C | T | C | A | C | C | A | G | G | C | G | G | A | T | G | T | T | C | G | G | A |
| 42 | UPEC-202 | G | G | C | T | T | A | C | C | G | G | T | G | C | C | A | A | C | G | G | G | C | T | G | G | T | G | C | C | T | T | G | A | C | A | C | T | A | C | G | G | G | C | A | A | A | C | T | C | A | C | C | A | G | G | C | G | G | A | T | G | T | T | C | G | G | A |
| 43 | UPEC-233 | G | G | C | T | T | A | C | C | G | G | T | G | C | C | A | A | C | G | G | G | C | C | G | G | T | G | C | C | T | T | G | A | C | A | G | T | A | C | G | G | G | C | A | A | A | C | T | C | A | C | C | A | G | G | C | G | G | A | T | G | T | T | C | G | G | A |
| 44 | 109 | G | G | C | T | T | A | C | C | G | G | T | G | C | C | A | A | C | G | G | G | C | C | G | G | T | G | C | C | T | T | G | A | C | A | G | T | A | C | G | G | G | C | A | A | A | C | T | C | A | C | C | A | G | G | C | G | G | A | T | G | T | T | C | G | G | A |
| 45 | UPEC-236 | G | G | C | T | T | A | C | C | G | G | T | G | C | C | A | A | C | G | G | G | C | C | G | G | T | G | C | C | T | T | G | A | C | A | G | T | A | C | G | G | G | C | A | A | A | C | T | C | A | C | C | A | G | G | C | G | G | A | T | G | T | T | C | G | G | A |
| 46 | NA114 | G | G | C | T | T | A | C | C | A | G | T | G | C | C | A | A | C | G | G | G | C | C | G | G | T | G | C | C | T | T | G | A | C | A | G | T | A | C | G | G | G | C | A | A | A | C | T | C | A | C | A | A | G | G | C | G | G | A | T | G | T | T | C | G | G | A |
| | 108 | G | G | C | T | T | A | C | C | A | G | T | G | C | C | A | A | C | G | G | G | C | C | G | G | T | G | C | C | T | T | G | A | C | A | G | T | A | C | G | G | G | C | A | A | A | C | T | C | A | C | A | A | G | G | C | G | G | A | T | G | T | T | C | G | G | A |
| | Ec958 | G | G | C | T | T | A | C | C | A | G | T | G | C | C | A | A | C | G | G | G | C | C | G | G | T | G | C | C | T | T | G | A | C | A | G | T | A | C | G | G | G | C | A | A | A | C | T | C | A | C | A | A | G | G | C | G | G | A | T | G | T | T | C | G | G | A |
| 47 | 66 | G | G | C | T | T | A | C | C | A | G | T | G | C | C | A | A | C | G | G | G | C | C | G | G | T | G | C | C | T | T | G | A | C | A | G | T | A | C | G | G | G | C | A | A | A | C | T | C | A | C | A | A | G | G | C | G | G | A | T | G | T | T | C | G | G | A |
| | 105 | G | G | C | T | T | A | C | C | A | G | T | G | C | C | A | A | C | G | G | G | C | C | G | G | T | G | C | C | T | T | G | A | C | A | G | T | A | C | G | G | G | C | A | A | A | C | T | C | A | C | A | A | G | G | C | G | G | A | T | G | T | T | C | G | G | A |
| 48 | 112 | G | G | C | T | T | A | C | C | G | G | T | G | C | C | A | A | T | G | G | G | C | C | G | G | T | G | C | C | T | T | G | A | C | A | G | T | A | C | G | G | G | C | A | A | A | C | T | C | A | C | C | A | G | G | C | G | G | A | T | G | T | T | C | G | G | A |
| 49 | 98 | G | G | C | T | T | A | C | C | A | G | T | G | C | C | A | A | C | G | G | G | C | C | G | G | T | G | C | C | T | T | G | A | C | A | G | T | A | C | G | G | G | C | A | A | A | C | T | C | A | C | A | A | G | G | C | G | G | A | T | G | T | T | C | G | G | A |
| 50 | 74 | G | G | C | T | T | A | C | C | G | G | T | T | C | C | A | A | C | G | G | G | C | C | G | G | T | G | C | C | T | T | G | A | C | A | G | T | A | C | G | G | G | C | A | A | A | C | T | C | A | C | C | A | G | G | C | G | G | A | T | G | T | T | C | G | G | A |
| 51 | 60 | G | G | C | C | T | A | C | C | G | G | T | G | C | C | A | A | C | A | G | G | C | C | G | G | T | G | C | C | T | T | G | A | C | A | G | T | A | C | G | G | G | C | A | A | A | C | T | C | A | C | C | A | G | G | C | G | G | A | T | G | T | T | C | G | G | A |
| 52 | 47 | G | G | C | T | T | A | C | C | G | G | T | G | C | C | A | A | C | G | G | G | C | C | G | G | T | G | C | C | T | T | G | A | C | A | G | T | A | C | G | G | G | C | A | A | A | C | T | C | A | C | C | A | G | G | C | G | G | A | T | G | T | T | C | G | G | A |
| 53 | UPEC-149 | G | G | C | T | T | A | C | C | G | G | T | G | C | C | A | A | C | G | G | G | C | T | G | G | T | G | C | C | T | T | G | A | C | A | C | T | A | C | G | G | G | C | A | A | A | C | T | C | A | C | C | A | G | G | C | G | G | A | T | G | T | T | C | G | G | A |
| 54 | 57 | G | G | C | T | T | A | C | C | G | G | T | G | C | C | A | A | C | G | G | G | C | T | G | G | T | G | C | C | T | T | G | A | C | A | C | T | A | C | G | G | G | C | A | A | A | C | T | C | A | C | C | A | G | G | C | G | G | A | T | G | T | T | C | G | G | A |
| | 59 | G | G | C | T | T | A | C | C | G | G | T | G | C | C | A | A | C | G | G | G | C | T | G | G | T | G | C | C | T | T | G | A | C | A | C | T | A | C | G | G | G | C | A | A | A | C | T | C | A | C | C | A | G | G | C | G | G | A | T | G | T | T | C | G | G | A |
| | 77 | G | G | C | T | T | A | C | C | G | G | T | G | C | C | A | A | C | G | G | G | C | T | G | G | T | G | C | C | T | T | G | A | C | A | C | T | A | C | G | G | G | C | A | A | A | C | T | C | A | C | C | A | G | G | C | G | G | A | T | G | T | T | C | G | G | A |
| | 90 | G | G | C | T | T | A | C | C | G | G | T | G | C | C | A | A | C | G | G | G | C | T | G | G | T | G | C | C | T | T | G | A | C | A | C | T | A | C | G | G | G | C | A | A | A | C | T | C | A | C | C | A | G | G | C | G | G | A | T | G | T | T | C | G | G | A |
| | UPEC-258 | G | G | C | T | T | A | C | C | G | G | T | G | C | C | A | A | C | G | G | G | C | T | G | G | T | G | C | C | T | T | G | A | C | A | C | T | A | C | G | G | G | C | A | A | A | C | T | C | A | C | C | A | G | G | C | G | G | A | T | G | T | T | C | G | G | A |
| 55 | 75 | G | G | C | T | T | A | C | C | A | G | T | G | C | C | A | A | C | G | G | G | T | T | G | G | T | G | C | C | T | T | G | A | C | A | C | T | A | C | G | G | G | C | T | C | A | C | T | C | A | C | C | A | G | G | T | A | G | G | T | G | T | T | C | G | G | A |
| 56 | 62 | G | G | C | T | A | G | A | C | G | G | C | G | C | T | A | G | C | G | G | G | C | T | A | G | T | G | C | C | C | A | G | G | T | A | C | C | A | C | A | G | G | C | A | A | A | C | C | C | A | C | A | A | G | G | C | G | G | A | T | G | T | T | C | G | G | A |
| | UPEC-137 | G | G | C | T | A | G | A | C | G | G | C | G | C | T | A | G | C | G | G | G | C | T | A | G | T | G | C | C | C | A | G | G | T | A | C | C | A | C | A | G | G | C | A | A | A | C | C | C | A | C | A | A | G | G | C | G | G | A | T | G | T | T | C | G | G | A |
| 57 | UPEC-213 | G | G | C | T | A | G | A | C | A | G | T | G | C | C | A | A | C | G | G | G | C | T | A | G | T | G | C | C | C | A | G | G | C | A | C | C | A | C | G | G | G | C | A | A | A | C | C | C | A | C | A | A | G | G | C | G | G | A | T | G | C | T | C | T | G | A |
| 58 | 4 | G | G | C | T | A | G | A | C | G | G | C | G | C | C | A | G | C | G | G | G | C | T | A | G | T | G | C | C | C | A | G | G | T | A | C | C | A | C | A | G | G | C | A | A | A | C | T | C | A | C | C | A | G | G | C | G | G | A | T | G | T | T | C | G | G | A |
| 59 | 3 | G | G | T | T | A | G | A | C | G | G | C | G | C | T | A | G | C | G | G | G | C | T | A | G | - | G | C | C | C | A | G | G | T | A | C | C | A | C | A | G | G | C | A | A | A | C | T | C | A | C | C | G | G | G | C | G | G | A | T | G | T | T | C | G | G | A |
| 60 | UPEC-103 | G | G | C | T | A | G | A | C | A | G | T | G | C | C | A | G | C | G | G | G | C | T | A | G | T | G | C | C | T | A | G | G | C | A | C | C | A | C | G | G | G | C | A | A | A | C | T | C | A | C | C | A | G | G | C | G | G | A | T | G | T | T | C | G | G | A |
| 61 | UPEC-200 | G | G | C | T | A | G | A | C | A | G | T | G | C | C | A | G | C | G | G | G | C | T | A | G | T | G | C | T | T | T | G | A | C | T | C | T | A | C | G | G | G | C | A | A | A | C | T | C | A | C | C | A | G | G | C | G | G | A | T | G | T | T | C | G | G | A |
| 62 | 54 | G | G | C | T | A | G | A | C | A | G | T | G | C | C | A | G | C | G | G | G | C | T | G | G | T | G | C | C | T | A | T | G | C | A | C | C | T | C | G | G | G | C | A | A | A | C | C | A | A | C | A | A | G | G | T | A | G | G | T | G | T | T | C | G | G | A |
| | 80 | G | G | C | T | A | G | A | C | A | G | T | G | C | C | A | G | C | G | G | G | C | T | G | G | T | G | C | C | T | A | T | G | C | A | C | C | T | C | G | G | G | C | A | A | A | C | C | A | A | C | A | A | G | G | T | A | G | G | T | G | T | T | C | G | G | A |
| 63 | 100 | G | G | C | T | A | G | A | C | A | G | T | G | C | C | A | G | C | G | G | G | C | T | G | G | T | G | C | C | T | A | T | G | C | A | C | C | T | C | G | G | G | C | A | A | A | C | C | A | A | C | A | A | G | G | T | A | G | G | T | G | T | T | C | G | G | A |
| 64 | 14 | G | G | C | T | A | G | A | T | A | G | T | G | C | C | A | G | C | G | G | G | C | T | A | G | T | G | C | C | T | A | G | G | C | A | C | C | A | C | G | T | G | C | A | A | A | C | C | A | A | C | A | A | G | G | T | A | G | G | T | G | T | T | C | G | G | A |
| 65 | 123 | G | G | C | T | A | G | A | C | A | G | T | G | C | C | A | G | C | G | G | G | C | T | A | G | T | G | C | C | T | A | G | G | C | A | C | C | A | C | G | T | G | C | A | A | A | C | C | A | A | C | A | A | G | G | T | A | G | G | T | G | T | T | C | G | G | A |
| 66 | 83 | G | G | C | T | A | G | A | C | A | G | T | G | C | C | A | G | C | A | G | G | C | T | G | G | T | A | C | C | T | A | G | G | T | A | C | C | A | C | G | G | G | C | A | A | A | C | C | A | A | C | A | A | G | G | T | A | G | G | T | G | T | T | C | G | G | A |

| | | | | | | | | | | | | | | | | | | | | | | | | | | | | | | | | | | | | | | | | | | | | | | | | | | | | | | | | | | | | | | | | | | | | |
|---|---|---|---|---|---|---|---|---|---|---|---|---|---|---|---|---|---|---|---|---|---|---|---|---|---|---|---|---|---|---|---|---|---|---|---|---|---|---|---|---|---|---|---|---|---|---|---|---|---|---|---|---|---|---|---|---|---|---|---|---|---|---|---|---|---|---|---|
| 67 | 64 | G | G | C | T | A | G | A | C | A | G | T | G | C | C | A | G | C | A | G | G | C | T | G | G | T | A | C | C | T | A | G | G | T | A | C | C | A | C | G | G | G | C | A | A | A | C | C | A | A | C | A | A | G | G | T | A | G | G | T | G | T | T | C | G | G | A |
| | UPEC-205 | G | G | C | T | A | G | A | C | A | G | T | G | C | C | A | G | C | A | G | G | C | T | G | G | T | A | C | C | T | A | G | G | T | A | C | C | A | C | G | G | G | C | A | A | A | C | C | A | A | C | A | A | G | G | T | A | G | G | T | G | T | T | C | G | G | A |
| 68 | UPEC-132 | G | G | C | T | T | A | A | C | A | G | T | G | C | C | A | G | C | G | G | G | C | T | G | G | T | G | C | C | T | T | G | A | C | T | C | T | A | C | G | G | G | C | A | A | A | C | C | C | A | C | A | A | G | G | T | A | G | G | T | G | T | T | G | G | G | A |
| 69 | 120 | G | G | C | T | T | A | A | C | A | G | T | G | C | C | A | G | C | G | G | G | C | T | G | G | T | G | C | C | T | T | G | A | C | T | C | T | A | C | G | G | G | C | A | A | A | C | C | C | A | C | A | A | G | G | T | A | G | G | T | G | T | T | G | G | G | A |
| 70 | UPEC-105 | G | G | C | T | A | G | A | C | A | G | C | G | C | C | A | G | C | G | G | G | C | T | G | G | T | G | C | C | C | A | G | G | C | A | C | C | A | C | G | G | G | C | T | A | A | C | C | A | A | C | A | A | G | G | T | A | G | G | T | G | T | T | C | G | G | A |
| 71 | UPEC-175 | A | G | C | T | A | G | A | C | A | G | C | G | C | C | A | G | C | G | G | G | C | T | G | G | T | G | C | C | C | A | G | G | T | G | C | C | A | C | G | G | G | C | T | A | G | C | C | C | A | A | A | A | G | G | C | G | G | A | T | G | C | T | C | G | G | A |
| 72 | UPEC-133 | G | G | C | T | A | G | A | C | A | A | C | G | C | C | A | G | C | G | G | G | C | T | G | G | T | G | C | C | C | A | G | G | C | A | C | C | A | T | G | G | G | C | T | A | A | C | C | A | A | C | A | A | G | G | T | A | G | G | T | G | T | C | C | G | G | A |
| 73 | 96 | G | G | C | T | A | G | A | C | A | G | C | G | C | C | A | G | C | G | G | G | C | T | G | G | T | G | C | C | C | A | G | G | C | A | C | C | A | T | G | G | G | C | T | A | A | C | C | A | A | C | A | A | G | G | T | A | G | G | T | G | T | T | C | G | G | A |
| 74 | 89 | G | G | C | T | A | G | A | C | A | G | T | G | C | C | A | G | C | G | G | G | C | T | G | G | T | G | C | C | T | A | T | G | C | A | C | C | T | C | G | G | G | C | A | A | A | C | C | A | A | C | A | A | G | G | T | A | G | G | T | G | T | T | C | G | G | A |
| | UPEC-127 | G | G | C | T | A | G | A | C | A | G | T | G | C | C | A | G | C | G | G | G | C | T | G | G | T | G | C | C | T | A | T | G | C | A | C | C | T | C | G | G | G | C | A | A | A | C | C | A | A | C | A | A | G | G | T | A | G | G | T | G | T | T | C | G | G | A |
| 75 | 55 | G | G | C | T | A | G | A | C | A | G | T | G | C | C | A | G | C | G | G | G | C | T | G | G | T | G | C | T | T | A | T | G | C | A | C | C | T | C | G | G | G | C | A | A | A | C | C | A | A | C | A | A | G | G | T | A | G | G | T | G | T | T | C | G | G | A |
| 76 | IAI39 | G | T | C | T | A | G | A | C | A | G | T | G | C | C | A | G | C | G | G | G | C | T | G | G | T | G | C | C | T | A | G | G | T | A | C | C | A | C | G | G | G | C | A | A | A | C | T | C | G | C | C | A | G | G | C | G | G | A | T | G | T | T | C | G | G | A |
| 77 | 124 | G | T | C | T | A | G | A | C | A | G | T | G | C | C | A | G | C | G | G | G | C | T | G | A | T | G | C | C | T | A | G | G | T | A | C | C | A | C | G | G | G | C | A | A | A | C | T | C | G | C | C | A | G | G | C | G | G | A | T | G | T | T | C | G | G | A |
| 78 | 76 | G | G | C | T | A | G | A | C | A | G | T | G | C | C | A | G | C | A | G | G | C | T | G | G | T | A | C | C | T | A | G | G | T | A | C | C | A | C | G | G | G | C | A | A | A | C | C | A | A | C | A | A | G | G | T | A | G | G | T | G | T | T | C | G | G | A |
| | UPEC-146 | G | G | C | T | A | G | A | C | A | G | T | G | C | C | A | G | C | A | G | G | C | T | G | G | T | A | C | C | T | A | G | G | T | A | C | C | A | C | G | G | G | C | A | A | A | C | C | A | A | C | A | A | G | G | T | A | G | G | T | G | T | T | C | G | G | A |
| 79 | 79 | G | G | C | T | A | G | A | C | A | G | T | G | C | C | A | G | C | A | G | G | C | T | G | G | T | A | C | C | T | A | G | G | T | A | C | C | A | C | G | G | G | C | A | A | A | C | C | A | A | C | A | A | G | G | T | A | G | G | T | G | T | T | C | G | G | A |
| 80 | UPEC-130 | G | G | C | T | A | G | A | C | A | G | T | G | C | C | A | G | C | A | G | G | C | T | G | G | T | A | C | C | T | A | G | G | T | A | C | C | A | C | G | G | G | C | A | A | A | C | C | A | A | C | A | A | G | G | T | A | G | G | T | G | T | T | C | G | G | A |
| 81 | 7 | G | G | C | T | A | G | A | C | A | G | T | G | C | C | A | G | C | G | G | G | C | G | G | G | T | G | C | C | T | A | G | G | C | A | C | C | A | C | G | G | G | C | A | A | A | C | C | A | A | C | A | A | A | G | T | A | G | G | T | G | T | T | C | G | G | G |
| 82 | UPEC-33 | G | G | C | T | A | G | A | C | A | G | T | G | C | C | A | G | C | G | G | G | C | T | G | G | T | G | C | C | T | A | G | G | C | A | C | C | A | C | G | G | G | C | A | A | A | C | C | A | A | C | A | A | G | G | T | A | G | G | T | G | T | T | C | G | G | G |
| 83 | UPEC-31 | G | G | C | T | A | G | A | C | A | G | T | G | C | C | A | G | C | G | G | G | C | T | G | G | T | G | C | C | T | A | G | G | C | A | C | C | A | C | G | G | G | C | A | A | A | C | C | A | A | C | A | A | G | G | T | A | G | G | T | G | T | T | C | G | G | G |
| 84 | UPEC-119 | G | G | C | T | A | G | A | C | A | G | T | G | C | C | A | G | C | G | G | G | C | T | G | G | T | G | C | C | T | A | G | G | C | A | C | C | A | C | G | G | G | C | A | A | A | C | C | A | A | C | A | A | G | G | T | A | G | G | T | G | T | T | C | G | G | G |
| 85 | 65 | G | G | C | T | A | G | A | C | A | G | T | G | C | C | A | G | C | A | G | G | C | T | G | G | T | A | C | C | T | A | G | G | T | A | C | C | A | C | G | G | G | C | A | A | A | C | C | A | A | C | A | A | G | G | T | A | G | G | T | G | T | T | C | G | G | A |
| 86 | 88 | G | G | C | T | A | G | A | C | A | G | T | G | C | C | A | G | C | A | G | G | C | T | G | G | T | A | C | C | T | A | G | G | T | A | C | C | A | C | G | G | G | C | A | A | A | C | C | A | A | C | A | A | G | G | T | A | G | G | T | G | T | T | C | G | G | A |
| 87 | 78 | G | G | C | T | A | G | A | C | A | G | T | G | C | C | A | G | C | A | G | G | C | T | G | G | T | A | C | C | T | A | G | G | T | A | C | C | A | C | G | G | G | C | A | A | A | C | C | A | A | C | A | A | G | G | T | A | G | G | T | G | T | T | C | G | G | A |
| 88 | 82 | G | G | C | T | A | G | A | C | A | G | T | G | C | C | A | G | C | A | G | G | C | T | G | G | T | A | C | C | T | A | G | G | T | A | C | C | A | C | G | G | G | C | A | A | A | C | C | A | A | C | A | A | G | G | T | A | G | G | T | G | T | T | C | G | G | A |
| 89 | UPEC-121 | G | G | C | T | A | G | A | C | A | G | T | G | C | C | A | G | C | G | G | G | C | T | G | G | T | G | C | C | T | A | G | G | C | A | C | C | A | C | G | G | G | C | A | A | A | C | C | A | A | C | A | A | G | G | T | A | G | G | T | G | T | T | C | G | G | G |
| 90 | 81 | G | G | C | T | A | G | A | C | A | G | T | G | C | C | A | G | C | A | G | G | C | T | G | G | T | A | C | C | T | A | G | G | T | A | C | C | A | C | G | G | G | C | A | A | A | C | C | A | A | C | A | A | G | G | T | A | G | G | T | G | T | T | C | G | G | A |
| 91 | 99 | G | G | C | T | A | G | A | C | A | G | T | G | C | C | A | G | C | A | G | G | C | T | G | G | T | A | C | C | T | A | G | G | T | A | C | C | A | C | G | G | G | C | A | A | A | C | C | A | A | C | A | A | G | G | T | A | G | G | T | G | T | T | C | G | G | A |
| 92 | UPEC-104 | G | G | C | T | A | G | A | C | A | G | T | G | C | C | A | G | C | A | G | G | C | T | G | G | T | A | C | C | T | A | G | G | T | A | C | C | A | C | G | G | G | C | A | A | A | C | C | A | A | C | A | A | G | G | T | A | G | G | T | G | T | T | C | G | G | A |
| 93 | UPEC-111 | G | G | C | T | A | G | A | C | A | G | T | G | C | C | A | G | C | A | G | G | C | T | G | G | T | A | C | C | T | A | G | G | T | A | C | C | A | C | G | G | G | C | A | A | A | C | C | A | A | C | A | A | G | G | T | A | G | G | T | A | T | T | C | G | G | A |
| 94 | 93 | G | G | C | T | A | G | A | C | A | G | T | G | C | C | A | G | C | A | G | G | C | T | G | G | T | A | C | C | T | A | G | G | T | A | C | C | A | C | G | G | G | C | A | A | A | C | C | A | A | C | A | A | G | G | T | A | G | G | T | G | T | T | C | G | G | A |
| 95 | UPEC-238 | G | G | C | T | A | G | A | C | A | G | T | G | C | C | A | G | C | A | G | G | C | T | G | G | T | A | C | C | T | A | G | G | T | A | C | C | A | C | G | G | G | C | A | A | A | C | C | A | A | C | A | A | G | G | T | A | G | G | T | G | T | T | C | G | G | A |
| 96 | 121 | G | G | C | T | A | G | A | C | A | G | T | G | C | C | A | G | C | A | G | G | C | T | G | G | T | A | C | C | T | A | G | G | T | A | C | C | A | C | G | G | G | C | A | A | A | C | C | A | A | C | A | A | G | G | T | A | G | G | T | G | T | T | C | G | G | A |
| 97 | UPEC-211 | G | G | C | T | T | A | A | C | A | G | T | G | C | C | A | G | C | G | G | G | C | T | G | G | T | G | C | C | T | A | G | G | T | A | C | C | A | C | G | G | A | C | A | A | A | C | C | C | A | C | A | A | G | G | T | A | G | G | T | G | T | T | C | G | G | A |
| 98 | 67 | G | G | C | T | T | A | A | C | A | G | T | G | C | C | A | G | C | G | G | G | C | T | G | G | T | G | C | C | T | A | G | G | T | A | C | C | A | C | G | G | A | C | A | A | A | C | C | C | A | C | A | A | G | G | T | A | G | G | T | G | T | T | C | G | G | A |
| | 102 | G | G | C | T | T | A | A | C | A | G | T | G | C | C | A | G | C | G | G | G | C | T | G | G | T | G | C | C | T | A | G | G | T | A | C | C | A | C | G | G | A | C | A | A | A | C | C | C | A | C | A | A | G | G | T | A | G | G | T | G | T | T | C | G | G | A |
| | 113 | G | G | C | T | T | A | A | C | A | G | T | G | C | C | A | G | C | G | G | G | C | T | G | G | T | G | C | C | T | A | G | G | T | A | C | C | A | C | G | G | A | C | A | A | A | C | C | C | A | C | A | A | G | G | T | A | G | G | T | G | T | T | C | G | G | A |
| 99 | UMN026 | G | G | C | T | A | G | A | C | A | G | C | G | C | C | A | G | C | G | G | G | C | T | G | G | T | G | C | C | C | A | G | G | C | A | C | C | A | T | G | G | G | C | T | A | A | C | C | A | A | C | A | A | G | G | T | A | G | G | T | G | T | T | C | G | G | A |
| | 84 | G | G | C | T | A | G | A | C | A | G | C | G | C | C | A | G | C | G | G | G | C | T | G | G | T | G | C | C | C | A | G | G | C | A | C | C | A | T | G | G | G | C | T | A | A | C | C | A | A | C | A | A | G | G | T | A | G | G | T | G | T | T | C | G | G | A |
| | 86 | G | G | C | T | A | G | A | C | A | G | C | G | C | C | A | G | C | G | G | G | C | T | G | G | T | G | C | C | C | A | G | G | C | A | C | C | A | T | G | G | G | C | T | A | A | C | C | A | A | C | A | A | G | G | T | A | G | G | T | G | T | T | C | G | G | A |
| | 92 | G | G | C | T | A | G | A | C | A | G | C | G | C | C | A | G | C | G | G | G | C | T | G | G | T | G | C | C | C | A | G | G | C | A | C | C | A | T | G | G | G | C | T | A | A | C | C | A | A | C | A | A | G | G | T | A | G | G | T | G | T | T | C | G | G | A |
| | 103 | G | G | C | T | A | G | A | C | A | G | C | G | C | C | A | G | C | G | G | G | C | T | G | G | T | G | C | C | C | A | G | G | C | A | C | C | A | T | G | G | G | C | T | A | A | C | C | A | A | C | A | A | G | G | T | A | G | G | T | G | T | T | C | G | G | A |
| | 104 | G | G | C | T | A | G | A | C | A | G | C | G | C | C | A | G | C | G | G | G | C | T | G | G | T | G | C | C | C | A | G | G | C | A | C | C | A | T | G | G | G | C | T | A | A | C | C | A | A | C | A | A | G | G | T | A | G | G | T | G | T | T | C | G | G | A |
| | 111 | G | G | C | T | A | G | A | C | A | G | C | G | C | C | A | G | C | G | G | G | C | T | G | G | T | G | C | C | C | A | G | G | C | A | C | C | A | T | G | G | G | C | T | A | A | C | C | A | A | C | A | A | G | G | T | A | G | G | T | G | T | T | C | G | G | A |
| | 118 | G | G | C | T | A | G | A | C | A | G | C | G | C | C | A | G | C | G | G | G | C | T | G | G | T | G | C | C | C | A | G | G | C | A | C | C | A | T | G | G | G | C | T | A | A | C | C | A | A | C | A | A | G | G | T | A | G | G | T | G | T | T | C | G | G | A |

| | | 558 | 573 | 575 | 585 | 609 | 631 | 633 | 650 | 675 | 684 | 708 | 713 | 716 | 759 | 765 | 808 | 824 | 838 | 845 | 846 | 849 | 856 | 861 | 897 | 900 | 915 | 926 | 927 | 930 | 954 | 978 | 986 | 988 | 1020 | 1027 | 1029 | 1036 | 1044 | 1057 | 1062 | 1096 | 1132 | 1134 | 1143 | 1152 | 1195 | 1205 | 1216 | 1245 | 1257 | 1260 | 1275 | 1276 | 1278 | 1279 | 1280 | 1281 | 1284 | 1293 | 1305 | 1321 | 1329 | 1330 | 1331 | 1359 | 1395 |
|---|---|---|---|---|---|---|---|---|---|---|---|---|---|---|---|---|---|---|---|---|---|---|---|---|---|---|---|---|---|---|---|---|---|---|---|---|---|---|---|---|---|---|---|---|---|---|---|---|---|---|---|---|---|---|---|---|---|---|---|---|---|---|---|---|---|---|---|
| 1 | 53 | G | G | C | T | C | C | G | C | T | T | A | A | C | A | T | C | C | A | G | C | G | C | C | C | C | G | C | T | C | C | G | C | G | G | G | G | C | C | A | G | T | C | T | G | C | C | C | G | C | C | T | T | A | C | G | C | A | C | C | C | C | T | G | C | A | C |
| 2 | Di2 | G | G | C | C | C | C | G | C | T | T | A | A | C | A | T | C | C | A | G | T | G | C | C | C | T | G | C | T | C | C | G | C | G | G | G | G | C | C | A | G | T | C | T | G | C | C | C | G | C | C | T | T | A | C | G | C | A | C | C | C | C | T | G | C | A | C |
| | Di14 | G | G | C | C | C | C | G | C | T | T | A | A | C | A | T | C | C | A | G | T | G | C | C | C | T | G | C | T | C | C | G | C | G | G | G | G | C | C | A | G | T | C | T | G | C | C | C | G | C | C | T | T | A | C | G | C | A | C | C | C | C | T | G | C | A | C |
| | CFT073 | G | G | C | C | C | C | G | C | T | T | A | A | C | A | T | C | C | A | G | T | G | C | C | C | T | G | C | T | C | C | G | C | G | G | G | G | C | C | A | G | T | C | T | G | C | C | C | G | C | C | T | T | A | C | G | C | A | C | C | C | C | T | G | C | A | C |
| 3 | 49 | G | G | C | C | C | C | G | C | T | T | A | A | C | A | T | C | C | A | G | T | G | C | C | C | T | G | C | T | C | C | G | C | G | G | G | G | C | C | A | G | T | C | T | G | C | C | C | G | C | C | T | T | A | C | G | C | A | C | C | C | C | T | G | C | A | C |
| | 87 | G | G | C | C | C | C | G | C | T | T | A | A | C | A | T | C | C | A | G | T | G | C | C | C | T | G | C | T | C | C | G | C | G | G | G | G | C | C | A | G | T | C | T | G | C | C | C | G | C | C | T | T | A | C | G | C | A | C | C | C | C | T | G | C | A | C |
| | 91 | G | G | C | C | C | C | G | C | T | T | A | A | C | A | T | C | C | A | G | T | G | C | C | C | T | G | C | T | C | C | G | C | G | G | G | G | C | C | A | G | T | C | T | G | C | C | C | G | C | C | T | T | A | C | G | C | A | C | C | C | C | T | G | C | A | C |
| | 95 | G | G | C | C | C | C | G | C | T | T | A | A | C | A | T | C | C | A | G | T | G | C | C | C | T | G | C | T | C | C | G | C | G | G | G | G | C | C | A | G | T | C | T | G | C | C | C | G | C | C | T | T | A | C | G | C | A | C | C | C | C | T | G | C | A | C |
| | 72 | G | G | C | C | C | C | G | C | T | T | A | A | C | A | T | C | C | A | G | T | G | C | C | C | T | G | C | T | C | C | G | C | G | G | G | G | C | C | A | G | T | C | T | G | C | C | C | G | C | C | T | T | A | C | G | C | A | C | C | C | C | T | G | C | A | C |
| 4 | UPEC-79 | G | G | C | C | C | C | G | C | T | T | A | A | C | A | T | C | C | A | G | T | G | C | C | C | T | G | C | T | C | C | G | C | G | G | G | G | C | C | A | G | T | C | T | G | C | C | C | G | C | C | T | T | A | C | G | C | A | C | C | C | C | T | G | C | A | C |
| 5 | 114 | G | G | C | C | C | C | G | C | T | T | A | A | C | A | T | C | C | A | G | T | G | C | C | C | T | G | C | T | C | C | G | C | G | G | G | G | C | C | A | G | T | C | T | G | C | C | C | G | C | C | T | T | A | C | G | C | A | C | C | C | C | T | G | T | A | C |
| 6 | ABU83972 | G | G | C | C | C | C | G | C | T | T | A | A | C | A | T | C | C | A | G | T | G | C | C | C | T | G | C | T | C | C | G | C | G | G | G | G | C | C | A | G | T | C | T | G | C | C | C | G | C | C | T | T | A | C | G | C | A | C | C | C | C | T | G | C | A | C |
| 7 | 94 | G | G | C | C | C | C | G | C | T | T | A | A | C | A | T | C | C | A | G | T | G | C | C | C | T | G | C | T | C | C | G | C | G | G | G | G | C | C | A | G | T | C | T | G | C | C | C | G | C | C | T | T | A | C | G | C | A | C | C | C | C | T | G | C | A | C |
| 8 | UPEC-116 | G | G | C | T | C | C | G | C | T | T | A | A | C | A | T | C | C | A | G | T | G | C | C | C | T | G | C | T | C | C | G | C | G | G | G | G | C | C | A | G | T | C | T | G | C | C | C | G | C | C | T | T | A | C | G | C | A | C | C | C | C | T | G | C | A | C |
| 9 | UPEC-180 | G | G | T | T | C | C | G | C | T | T | A | A | C | A | T | C | C | A | G | T | G | T | C | C | T | G | C | T | C | C | G | C | G | G | G | G | C | C | A | G | T | C | T | G | C | C | C | G | C | C | T | T | A | C | G | C | A | C | C | C | C | T | G | C | A | C |
| 10 | UPEC-100 | G | G | C | C | C | C | G | C | T | T | A | A | C | A | T | C | C | A | G | T | G | C | C | C | T | G | C | T | C | C | G | C | G | G | G | G | C | C | A | G | T | C | T | G | C | C | C | G | C | C | T | T | A | C | G | C | A | C | C | C | C | T | G | C | A | C |
| 11 | UPEC-112 | G | G | C | C | C | C | G | C | T | T | A | A | C | A | T | C | C | A | G | T | G | C | C | C | T | G | C | T | C | C | G | C | G | G | G | G | C | C | A | G | T | C | T | G | C | C | C | G | C | C | T | T | A | C | G | C | A | C | C | C | C | T | G | C | A | C |
| 12 | 85 | G | G | C | T | C | C | G | C | T | T | A | A | C | A | T | C | C | A | G | C | G | C | T | C | C | G | C | C | C | C | G | C | G | G | G | G | C | C | A | G | T | C | T | G | T | C | C | G | C | C | T | T | A | C | G | C | A | C | C | C | C | T | G | C | A | C |
| | UPEC-115 | G | G | C | T | C | C | G | C | T | T | A | A | C | A | T | C | C | A | G | C | G | C | T | C | C | G | C | C | C | C | G | C | G | G | G | G | C | C | A | G | T | C | T | G | T | C | C | G | C | C | T | T | A | C | G | C | A | C | C | C | C | T | G | C | A | C |
| 13 | UTI89 | G | G | C | T | C | C | G | C | T | T | A | A | C | A | T | C | C | A | G | C | G | C | T | C | C | G | C | C | C | C | G | C | G | G | G | G | C | C | A | G | T | C | T | G | T | C | C | G | C | C | T | T | A | C | G | C | A | C | C | C | C | T | G | C | A | C |
| 14 | UPEC-153 | G | G | C | T | C | C | G | C | T | T | A | A | C | A | T | C | C | A | G | C | G | C | C | A | T | G | C | T | C | C | G | C | G | G | G | G | C | C | A | G | T | C | T | G | C | C | C | G | C | C | T | T | A | T | G | C | T | C | C | C | C | T | G | C | G | C |
| 15 | 101 | G | G | C | T | C | C | G | C | T | T | A | A | C | A | T | C | C | A | G | C | G | C | T | C | C | G | C | C | C | C | G | C | G | G | G | G | C | C | A | G | T | C | T | G | T | C | C | G | C | C | T | T | A | C | G | C | T | C | C | C | C | T | G | C | G | C |
| 16 | 122 | G | G | C | T | C | C | G | C | T | T | A | A | C | A | T | C | C | A | G | C | G | C | T | C | C | G | C | C | C | C | G | C | G | G | G | G | C | C | A | G | T | C | T | G | T | C | C | G | C | C | T | T | A | C | G | C | T | C | C | C | C | T | G | C | G | C |
| 17 | 11 | G | G | C | T | C | C | G | C | T | T | A | A | C | A | T | C | C | A | G | C | G | C | T | C | C | G | C | C | C | C | G | C | G | G | G | G | C | C | A | G | T | C | T | G | T | C | C | G | C | C | T | T | A | C | G | C | T | C | C | C | C | T | G | T | G | C |
| 18 | 97 | G | G | C | T | C | C | G | C | T | T | A | A | C | A | T | C | C | A | G | C | G | C | T | C | C | G | C | C | C | C | G | C | G | G | G | G | C | C | A | G | T | C | T | G | T | C | C | G | C | C | T | T | A | C | G | C | T | C | C | C | C | T | G | C | G | C |
| 19 | UPEC-195 | G | G | C | T | C | C | G | C | T | T | A | A | C | A | T | C | C | A | G | C | G | C | T | C | C | G | C | C | C | C | G | C | G | G | G | G | C | C | A | G | T | C | T | G | T | C | C | G | C | C | T | T | A | C | G | C | T | C | C | C | C | T | G | C | G | C |
| 20 | UPEC-150 | G | G | C | C | C | C | G | C | T | T | A | A | C | A | T | C | C | A | G | C | G | C | T | C | C | G | C | C | C | C | G | C | G | G | G | G | C | C | A | G | T | C | T | G | T | C | C | G | C | C | T | T | A | C | G | C | T | C | C | C | C | T | G | C | G | C |
| 21 | UPEC-101 | G | G | C | T | C | C | G | C | C | T | A | A | C | A | T | C | C | A | G | C | G | C | T | C | C | G | C | C | C | C | G | C | G | G | G | G | C | C | A | G | T | C | T | G | T | C | C | G | C | C | T | T | A | C | G | C | T | C | C | C | C | T | G | C | G | C |
| 22 | UPEC-59 | G | G | C | T | C | C | G | T | C | T | A | A | C | A | T | C | C | A | G | C | G | C | T | C | C | G | C | C | C | C | G | C | G | G | G | G | C | C | A | G | T | C | T | G | T | C | C | G | C | C | T | T | A | C | G | C | T | C | C | C | C | T | G | C | G | C |
| 23 | 119 | G | G | C | T | C | C | G | C | T | T | A | A | C | A | T | C | C | A | G | C | G | C | T | C | C | G | C | C | C | C | G | C | G | G | G | G | C | C | A | G | T | C | T | G | T | C | C | G | C | C | T | T | A | C | G | C | T | C | C | C | C | T | G | C | G | C |
| 24 | 8 | G | G | C | T | C | C | G | C | T | T | A | A | C | A | T | C | C | A | G | C | G | C | T | C | C | G | C | C | C | C | G | C | G | G | G | G | C | C | A | G | T | C | T | G | T | C | C | G | C | C | T | T | A | C | G | C | T | C | C | C | C | T | G | C | G | C |
| 25 | 1 | G | G | C | T | C | C | G | C | T | T | A | A | C | A | T | C | C | A | G | C | G | C | T | C | C | G | C | C | C | C | G | C | G | G | G | G | C | C | A | G | T | C | T | G | T | C | C | G | C | C | T | T | A | C | G | C | T | C | C | C | C | T | G | T | G | C |
| 26 | 58 | G | G | C | T | C | C | G | C | T | T | A | A | C | A | T | C | C | A | G | C | G | C | T | C | C | G | C | C | C | C | G | C | G | G | G | G | C | C | A | G | T | C | T | G | T | C | C | G | C | C | T | T | A | C | G | C | T | C | C | C | C | T | G | T | G | C |
| | 71 | G | G | C | T | C | C | G | C | T | T | A | A | C | A | T | C | C | A | G | C | G | C | T | C | C | G | C | C | C | C | G | C | G | G | G | G | C | C | A | G | T | C | T | G | T | C | C | G | C | C | T | T | A | C | G | C | T | C | C | C | C | T | G | T | G | C |
| 27 | 68 | G | G | C | T | C | C | G | C | T | T | A | A | C | A | T | C | C | A | G | C | G | C | T | C | C | G | C | C | C | C | G | C | G | G | G | G | C | C | A | G | T | C | T | G | T | C | C | G | C | C | T | T | A | C | A | C | T | C | C | C | C | T | G | T | G | C |
| 28 | 107 | G | G | C | T | C | C | G | C | T | T | A | A | C | A | T | C | C | A | G | C | G | C | T | C | C | G | C | C | C | C | G | C | G | G | G | G | C | C | A | G | T | C | T | G | T | C | C | G | C | C | T | T | A | C | G | C | A | C | C | C | C | T | A | C | G | C |
| 29 | UPEC-179 | G | G | C | T | C | C | G | C | T | T | A | A | C | A | T | C | C | A | G | T | G | C | C | C | T | G | C | T | C | C | G | C | G | G | G | G | C | C | A | G | T | C | T | G | C | C | C | G | C | C | T | T | A | C | G | C | A | C | C | C | C | T | G | C | G | C |
| 30 | UPEC-78 | G | G | C | C | C | C | G | C | T | T | A | A | C | A | T | C | C | A | G | T | G | C | C | C | T | G | C | T | C | C | G | C | G | G | G | G | C | C | A | G | T | C | T | G | C | C | C | G | C | C | T | T | A | C | G | C | A | C | C | C | C | T | G | C | G | C |
| 31 | UPEC-274 | G | G | C | T | C | C | G | C | T | T | A | A | C | A | T | C | T | A | G | C | G | C | C | C | T | G | C | T | C | C | G | C | G | G | G | G | C | C | A | G | T | C | T | G | C | C | C | G | C | C | T | T | A | C | G | C | T | C | C | C | C | T | G | C | G | C |
| 32 | UPEC-243 | G | G | C | C | C | C | G | C | T | T | A | A | C | A | T | C | C | A | G | C | G | C | C | C | T | G | C | T | C | C | G | C | G | G | G | G | C | C | A | G | T | C | T | G | C | C | C | G | C | C | T | T | A | T | G | C | T | C | C | C | C | T | G | C | G | C |

| | | | | | | | | | | | | | | | | | | | | | | | | | | | | | | | | | | | | | | | | | | | | | | | | | | | | | | | | | | | | | | | | | | | |
|---|---|---|---|---|---|---|---|---|---|---|---|---|---|---|---|---|---|---|---|---|---|---|---|---|---|---|---|---|---|---|---|---|---|---|---|---|---|---|---|---|---|---|---|---|---|---|---|---|---|---|---|---|---|---|---|---|---|---|---|---|---|---|---|---|---|---|---|
| 33 | 5 | G | G | C | C | C | C | G | C | T | T | A | A | C | A | T | C | T | A | G | C | G | C | C | C | T | G | C | T | C | C | G | C | G | G | G | G | C | C | A | G | T | C | T | G | C | C | C | G | C | C | T | T | A | C | G | C | T | C | C | C | C | T | G | C | G | C |
| 34 | 70 | G | G | C | C | C | C | G | C | T | T | A | A | C | A | T | C | T | A | G | C | G | C | C | C | T | G | C | T | C | C | G | C | G | G | G | G | C | C | A | G | T | C | T | G | C | C | C | G | C | C | T | T | A | C | G | C | T | C | C | C | C | T | G | C | G | C |
| | 115 | G | G | C | C | C | C | G | C | T | T | A | A | C | A | T | C | T | A | G | C | G | C | C | C | T | G | C | T | C | C | G | C | G | G | G | G | C | C | A | G | T | C | T | G | C | C | C | G | C | C | T | T | A | C | G | C | T | C | C | C | C | T | G | C | G | C |
| 35 | 536 | G | G | C | C | C | C | G | C | T | T | A | A | C | A | T | C | T | A | G | C | G | C | C | C | T | G | C | T | C | C | G | C | G | G | G | G | C | C | A | G | T | C | T | G | C | C | C | G | C | C | T | T | A | C | G | C | T | C | T | C | C | T | G | C | G | C |
| 36 | F11 | G | G | C | C | C | C | G | C | T | T | A | A | C | A | T | C | T | A | G | C | G | C | C | C | T | G | C | T | C | C | G | C | G | G | G | G | C | C | A | G | T | C | T | G | C | C | C | G | C | C | T | T | A | C | G | C | T | C | C | C | C | T | G | C | G | C |
| 37 | UPEC-38 | G | G | C | T | C | C | G | C | T | G | G | G | C | A | T | C | C | A | G | C | G | C | C | C | C | G | C | T | C | C | G | C | G | G | C | A | C | T | A | G | C | C | T | G | C | C | C | G | C | C | T | T | A | C | G | C | A | C | C | C | C | T | G | C | G | C |
| 38 | UPEC-58 | G | G | C | C | C | C | G | C | T | G | G | G | C | A | T | C | C | A | G | C | G | C | C | C | C | G | C | T | C | C | G | C | G | G | C | A | C | T | A | G | C | C | T | G | C | C | C | G | T | C | T | T | A | C | G | C | A | C | C | C | C | T | G | C | G | C |
| 39 | UPEC-219 | G | G | C | T | C | C | G | T | T | G | G | A | C | A | T | T | C | A | G | C | G | C | C | C | T | G | C | T | C | C | G | C | G | G | G | G | C | C | A | G | T | C | T | G | C | C | C | G | C | C | T | T | A | C | G | C | A | C | C | C | C | T | G | C | G | C |
| 40 | 69 | G | G | C | T | C | C | G | C | T | G | G | A | C | A | T | T | C | A | G | C | G | C | C | C | T | G | C | T | C | C | G | C | G | G | G | G | C | C | A | G | T | C | T | G | C | C | C | G | C | C | T | T | A | C | G | C | A | C | C | C | C | T | G | C | G | C |
| | 110 | G | G | C | T | C | C | G | C | T | G | G | A | C | A | T | T | C | A | G | C | G | C | C | C | T | G | C | T | C | C | G | C | G | G | G | G | C | C | A | G | T | C | T | G | C | C | C | G | C | C | T | T | A | C | G | C | A | C | C | C | C | T | G | C | G | C |
| 41 | UPEC-266 | G | G | C | T | C | C | G | C | T | T | A | A | C | A | T | C | T | A | G | C | G | C | C | C | T | G | C | T | C | C | G | C | G | G | G | G | C | C | A | G | T | C | T | G | C | C | C | G | C | C | T | T | A | C | G | C | A | C | C | C | C | T | G | C | G | C |
| 42 | UPEC-202 | G | G | C | T | C | C | G | C | T | G | G | A | C | A | C | C | C | A | G | C | G | C | C | C | T | G | C | T | C | C | G | C | G | G | G | G | C | C | A | G | T | C | T | G | C | C | C | G | C | C | T | T | A | C | G | C | A | C | C | C | C | T | G | C | G | C |
| 43 | UPEC-233 | G | G | C | T | C | C | G | C | T | G | A | A | C | A | C | C | C | A | G | C | G | C | C | T | C | A | C | C | T | C | G | C | G | A | C | G | A | C | A | G | C | C | T | G | C | C | A | G | C | C | T | C | A | C | G | C | T | C | C | C | A | C | G | C | G | C |
| 44 | 109 | G | G | C | T | C | C | G | C | T | G | A | A | C | A | C | C | C | A | G | C | G | C | C | T | C | A | C | C | T | C | G | C | G | A | C | G | C | C | A | G | C | C | T | G | C | C | A | A | C | C | T | C | A | C | G | C | T | C | C | C | C | C | G | C | G | C |
| 45 | UPEC-236 | G | G | C | T | C | C | G | C | T | T | A | A | C | A | T | C | C | A | G | C | G | C | C | C | C | G | C | T | C | C | G | C | G | A | C | G | C | C | A | G | C | C | T | G | C | C | C | G | C | C | T | T | A | A | G | C | T | C | C | T | C | C | G | C | A | T |
| 46 | NA114 | G | G | C | T | C | C | G | C | T | G | A | G | T | A | C | T | C | G | G | C | G | C | C | T | C | A | C | C | T | C | G | C | T | G | C | A | C | T | C | G | C | C | T | G | C | C | C | G | C | C | T | T | A | C | G | C | T | T | C | T | C | T | G | C | A | T |
| | 108 | G | G | C | T | C | C | G | C | T | G | A | G | T | A | C | T | C | G | G | C | G | C | C | T | C | A | C | C | T | C | G | C | T | G | C | A | C | T | C | G | C | C | T | G | C | C | C | G | C | C | T | T | A | C | G | C | T | T | C | T | C | T | G | C | A | T |
| | Ec958 | G | G | C | T | C | C | G | C | T | G | A | G | T | A | C | T | C | G | G | C | G | C | C | T | C | A | C | C | T | C | G | C | T | G | C | A | C | T | C | G | C | C | T | G | C | C | C | G | C | C | T | T | A | C | G | C | T | T | C | T | C | T | G | C | A | T |
| 47 | 66 | G | G | C | T | C | C | G | C | T | G | A | G | T | A | C | T | C | G | G | C | G | C | C | T | C | A | C | C | T | C | G | C | T | G | C | A | C | T | C | G | C | C | T | G | C | C | C | G | C | C | T | T | A | C | G | C | T | T | C | T | C | T | G | C | A | T |
| | 105 | G | G | C | T | C | C | G | C | T | G | A | G | T | A | C | T | C | G | G | C | G | C | C | T | C | A | C | C | T | C | G | C | T | G | C | A | C | T | C | G | C | C | T | G | C | C | C | G | C | C | T | T | A | C | G | C | T | T | C | T | C | T | G | C | A | T |
| 48 | 112 | G | G | C | T | C | C | G | C | T | G | A | G | T | A | C | T | C | G | G | C | G | C | C | T | C | A | C | C | T | C | G | C | T | G | C | A | C | T | C | G | C | C | T | G | C | C | C | G | C | C | T | T | A | C | G | C | T | T | C | T | C | T | G | C | A | T |
| 49 | 98 | G | G | C | T | C | C | G | C | T | G | G | A | C | G | C | C | C | A | G | C | G | C | T | T | C | G | C | T | C | C | G | C | T | G | C | A | C | T | C | G | C | C | T | G | C | C | C | G | C | C | T | T | A | C | G | C | T | C | C | C | C | C | G | T | G | T |
| 50 | 74 | G | G | C | T | C | C | G | C | T | G | G | A | C | G | C | C | C | A | G | C | G | C | T | T | C | G | C | T | C | C | G | C | T | G | C | A | C | T | C | G | C | C | T | G | C | C | C | G | C | C | T | T | A | C | G | C | T | C | C | C | C | C | G | T | G | T |
| 51 | 60 | G | G | C | T | C | C | G | C | T | G | G | A | C | G | C | C | C | A | G | C | G | C | T | T | C | G | C | T | C | C | G | C | T | G | C | A | C | T | C | G | C | C | T | G | C | C | C | G | C | C | T | T | A | C | G | C | T | C | C | C | C | C | G | T | G | T |
| 52 | 47 | G | G | C | T | C | C | G | C | T | G | G | A | C | G | C | C | C | A | G | C | G | C | T | T | C | G | C | T | C | C | G | C | T | G | C | A | C | T | C | G | C | C | T | G | C | C | C | G | C | C | T | T | A | C | G | C | T | C | C | C | C | C | G | T | G | T |
| 53 | UPEC-149 | G | G | C | T | C | C | G | C | T | G | G | A | C | G | C | C | C | A | G | C | G | C | T | T | C | G | C | T | C | C | G | C | T | G | C | A | C | T | C | G | C | C | T | G | C | C | C | G | C | C | T | T | A | C | G | C | T | C | C | C | C | C | G | T | G | T |
| 54 | 57 | G | G | C | T | C | C | G | C | T | G | G | A | C | G | C | C | C | A | G | C | G | C | T | T | C | G | C | T | C | C | G | C | T | G | C | A | C | T | C | G | C | C | T | G | C | C | C | G | C | C | T | T | A | C | G | C | T | C | C | C | C | C | G | T | G | T |
| | 59 | G | G | C | T | C | C | G | C | T | G | G | A | C | G | C | C | C | A | G | C | G | C | T | T | C | G | C | T | C | C | G | C | T | G | C | A | C | T | C | G | C | C | T | G | C | C | C | G | C | C | T | T | A | C | G | C | T | C | C | C | C | C | G | T | G | T |
| | 77 | G | G | C | T | C | C | G | C | T | G | G | A | C | G | C | C | C | A | G | C | G | C | T | T | C | G | C | T | C | C | G | C | T | G | C | A | C | T | C | G | C | C | T | G | C | C | C | G | C | C | T | T | A | C | G | C | T | C | C | C | C | C | G | T | G | T |
| | 90 | G | G | C | T | C | C | G | C | T | G | G | A | C | G | C | C | C | A | G | C | G | C | T | T | C | G | C | T | C | C | G | C | T | G | C | A | C | T | C | G | C | C | T | G | C | C | C | G | C | C | T | T | A | C | G | C | T | C | C | C | C | C | G | T | G | T |
| | UPEC-258 | G | G | C | T | C | C | G | C | T | G | G | A | C | G | C | C | C | A | G | C | G | C | T | T | C | G | C | T | C | C | G | C | T | G | C | A | C | T | C | G | C | C | T | G | C | C | C | G | C | C | T | T | A | C | G | C | T | C | C | C | C | C | G | T | G | T |
| 55 | 75 | G | G | C | T | C | G | G | C | T | G | G | A | C | A | C | C | C | A | G | C | G | C | C | T | C | A | C | C | T | C | G | C | G | A | C | G | C | C | A | G | C | C | T | G | C | C | C | G | C | T | T | C | A | C | G | C | T | C | C | C | C | C | G | C | G | T |
| 56 | 62 | G | G | C | C | C | C | G | C | T | T | A | A | C | A | T | C | C | A | G | C | G | C | C | C | T | G | C | C | C | C | G | C | G | G | G | G | C | C | A | G | T | C | T | G | T | A | C | G | C | C | T | T | A | C | G | C | A | C | C | C | C | T | G | C | A | C |
| | UPEC-137 | G | G | C | C | C | C | G | C | T | T | A | A | C | A | T | C | C | A | G | C | G | C | C | C | T | G | C | C | C | C | G | C | G | G | G | G | C | C | A | G | T | C | T | G | T | A | C | G | C | C | T | T | A | C | G | C | A | C | C | C | C | T | G | C | A | C |
| 57 | UPEC-213 | G | G | C | T | C | C | G | C | T | G | G | G | C | A | C | C | C | A | G | C | G | C | C | C | C | A | C | C | C | C | G | C | G | A | C | G | C | C | A | T | C | C | T | G | C | C | C | G | C | C | T | T | A | C | G | C | T | C | C | C | C | C | G | C | A | C |
| 58 | 4 | G | G | C | C | C | C | G | C | T | G | A | A | C | A | C | C | C | A | G | C | G | C | C | T | C | G | C | C | T | C | G | C | G | G | C | G | C | C | A | G | C | C | T | G | C | A | C | G | C | C | T | T | A | C | G | C | T | C | C | C | C | C | G | T | A | C |
| 59 | 3 | G | G | C | C | C | C | G | C | T | T | A | A | C | A | T | C | C | A | G | C | G | C | C | C | C | G | C | T | C | C | G | C | G | A | C | G | C | C | A | G | C | C | T | G | C | C | C | G | C | C | T | T | A | A | G | C | T | C | C | T | C | C | G | C | A | T |
| 60 | UPEC-103 | G | G | C | T | C | C | G | C | T | G | G | A | C | A | C | T | C | G | A | C | G | C | C | T | C | A | A | C | T | C | G | C | T | G | C | A | C | T | C | G | T | C | T | G | C | C | C | G | C | C | T | T | A | C | G | C | T | T | C | T | C | T | G | C | A | T |
| 61 | UPEC-200 | G | G | C | T | C | C | G | C | T | G | A | G | T | A | C | T | C | A | G | C | G | C | C | T | C | A | C | C | T | C | G | C | T | G | C | A | C | T | C | G | T | C | T | G | C | C | C | G | C | C | T | T | A | C | G | C | T | C | C | T | C | T | G | C | A | T |
| 62 | 54 | G | G | C | C | C | C | G | C | T | G | A | A | C | A | C | C | C | A | G | C | G | C | C | T | C | A | C | C | T | C | G | C | G | A | C | G | A | C | A | G | C | C | T | G | C | C | A | G | C | C | T | C | A | C | G | C | T | C | C | C | C | C | G | C | G | C |
| | 80 | G | G | C | C | C | C | G | C | T | G | A | A | C | A | C | C | C | A | G | C | G | C | C | T | C | A | C | C | T | C | G | C | G | A | C | G | A | C | A | G | C | C | T | G | C | C | A | G | C | C | T | C | A | C | G | C | T | C | C | C | C | C | G | C | G | C |
| 63 | 100 | G | G | C | C | C | C | G | C | T | G | A | A | C | A | C | C | C | A | G | C | G | C | C | T | C | A | C | C | T | C | G | T | G | A | C | G | A | C | A | G | C | C | T | G | C | C | A | G | C | C | T | C | A | C | G | C | T | C | C | C | C | C | G | C | G | C |
| 64 | 14 | A | G | C | T | C | C | G | C | T | G | A | A | C | A | C | C | C | A | G | C | G | C | C | T | C | A | C | C | T | C | G | T | G | A | C | G | A | C | A | G | C | C | T | G | C | C | A | G | C | C | T | C | A | C | G | C | T | C | C | C | C | C | G | C | G | C |
| 65 | 123 | A | G | C | T | C | C | G | C | T | G | A | A | C | A | C | C | C | A | G | C | G | C | C | T | C | A | C | C | T | C | G | C | G | A | C | G | C | C | A | G | C | C | T | G | C | C | A | A | C | C | T | C | A | C | G | C | T | C | C | C | C | C | G | C | G | C |
| 66 | 83 | G | G | C | T | C | C | G | C | T | G | A | A | C | A | C | C | C | A | G | C | G | C | C | T | C | A | C | C | T | C | G | T | G | A | C | G | A | C | A | G | C | C | T | G | C | C | A | G | C | C | T | C | A | C | G | C | T | C | C | C | C | C | G | C | G | C |

| | | | | | | | | | | | | | | | | | | | | | | | | | | | | | | | | | | | | | | | | | | | | | | | | | | | | | | | | | | | | | | | | | | | |
|---|---|---|---|---|---|---|---|---|---|---|---|---|---|---|---|---|---|---|---|---|---|---|---|---|---|---|---|---|---|---|---|---|---|---|---|---|---|---|---|---|---|---|---|---|---|---|---|---|---|---|---|---|---|---|---|---|---|---|---|---|---|---|---|---|---|---|---|
| 67 | 64 | G | G | C | T | C | C | G | C | T | G | A | A | C | A | C | C | C | A | G | C | G | C | C | T | C | A | C | C | T | C | G | T | G | A | C | G | A | C | A | G | C | C | T | G | C | C | A | G | C | C | T | C | A | C | G | C | T | C | C | C | C | C | G | C | G | C |
| | UPEC-205 | G | G | C | T | C | C | G | C | T | G | A | A | C | A | C | C | C | A | G | C | G | C | C | T | C | A | C | C | T | C | G | T | G | A | C | G | A | C | A | G | C | C | T | G | C | C | A | G | C | C | T | C | A | C | G | C | T | C | C | C | C | C | G | C | G | C |
| 68 | UPEC-132 | G | G | C | T | C | C | G | C | T | G | G | A | C | G | C | C | C | A | G | C | G | C | T | T | C | A | C | C | T | C | G | C | G | A | C | G | A | C | A | G | C | C | T | G | C | C | A | G | C | C | T | C | A | C | G | C | T | C | C | C | C | C | G | C | G | C |
| 69 | 120 | G | G | C | T | C | C | G | C | T | G | A | A | C | A | C | C | C | A | G | C | G | C | C | T | C | A | C | C | T | C | G | C | G | A | C | G | A | C | A | G | C | C | T | G | C | C | A | G | C | C | T | C | A | C | G | C | T | C | C | C | C | C | G | C | G | C |
| 70 | UPEC-105 | G | G | C | T | C | C | G | C | T | G | A | G | C | A | C | C | C | A | G | C | G | C | C | T | C | A | C | C | T | C | G | C | T | A | C | G | C | C | A | G | C | C | T | G | C | C | C | G | C | C | T | T | A | C | G | C | T | C | C | C | C | C | G | C | A | C |
| 71 | UPEC-175 | G | G | C | T | C | C | T | C | T | G | A | A | C | A | C | C | C | A | G | C | G | C | C | T | C | A | C | C | C | C | G | C | G | A | C | G | C | C | A | G | C | C | T | G | C | C | C | G | C | C | T | T | A | C | G | C | T | C | C | C | C | C | G | T | G | T |
| 72 | UPEC-133 | G | G | C | T | T | C | G | C | T | T | A | A | C | A | T | C | C | A | G | C | G | C | C | T | C | A | C | C | T | C | G | C | G | A | C | G | C | C | C | G | C | C | T | G | C | C | C | G | C | C | T | T | A | C | G | C | T | C | C | C | C | C | G | C | A | C |
| 73 | 96 | G | G | C | T | C | C | G | C | T | G | A | A | C | A | C | C | C | A | G | C | G | C | C | T | C | A | C | C | C | C | G | C | G | A | C | G | C | C | A | G | C | C | T | G | C | C | C | G | C | C | T | C | A | C | G | C | T | C | C | C | C | C | G | C | A | C |
| 74 | 89 | G | G | C | C | C | C | G | C | T | G | G | A | C | G | C | C | C | A | G | C | G | C | T | T | C | G | C | T | C | C | G | C | T | G | C | A | C | T | C | G | C | C | T | G | C | C | C | G | C | C | T | T | A | C | G | C | T | C | C | C | C | C | G | T | G | T |
| | UPEC-127 | G | G | C | C | C | C | G | C | T | G | G | A | C | G | C | C | C | A | G | C | G | C | T | T | C | G | C | T | C | C | G | C | T | G | C | A | C | T | C | G | C | C | T | G | C | C | C | G | C | C | T | T | A | C | G | C | T | C | C | C | C | C | G | T | G | T |
| 75 | 55 | G | G | C | C | C | C | G | C | T | G | G | A | C | G | C | C | C | A | G | C | G | C | T | T | C | G | C | T | C | C | G | C | T | G | C | A | C | T | C | G | C | C | T | G | C | C | C | G | C | C | T | T | A | C | G | C | T | C | C | C | C | C | G | T | G | T |
| 76 | IAI39 | G | G | C | C | C | C | T | C | T | G | G | A | C | G | C | C | C | A | G | C | G | C | T | T | C | A | C | C | C | T | G | C | T | G | C | A | C | T | C | G | C | C | T | A | C | C | C | G | C | C | T | T | A | A | G | C | T | C | C | T | C | T | G | C | A | T |
| 77 | 124 | G | G | C | C | C | C | T | C | T | G | G | A | C | G | C | C | C | A | G | C | G | C | T | T | C | A | C | C | C | T | G | C | T | G | C | A | C | T | C | G | C | C | T | A | C | C | C | G | C | C | T | T | A | A | G | C | T | C | C | T | C | T | G | C | A | T |
| 78 | 76 | G | G | C | T | C | C | G | C | T | G | G | A | C | G | C | C | C | A | G | C | G | C | T | T | C | A | C | C | C | T | G | C | T | G | C | A | C | T | C | G | C | C | T | A | C | C | C | G | C | C | T | T | A | A | G | C | T | C | C | T | C | T | G | C | A | T |
| | UPEC-146 | G | G | C | T | C | C | G | C | T | G | G | A | C | G | C | C | C | A | G | C | G | C | T | T | C | A | C | C | C | T | G | C | T | G | C | A | C | T | C | G | C | C | T | A | C | C | C | G | C | C | T | T | A | A | G | C | T | C | C | T | C | T | G | C | A | T |
| 79 | 79 | G | G | C | T | C | C | G | C | T | G | G | A | C | G | C | C | C | A | G | C | G | C | T | T | C | A | C | C | C | T | G | C | T | G | C | A | C | T | C | G | C | C | T | A | C | C | C | G | C | C | T | T | A | A | G | C | T | C | C | T | C | T | G | C | A | T |
| 80 | UPEC-130 | G | G | C | T | C | C | G | C | T | G | G | A | C | G | C | C | C | A | G | C | G | C | T | T | C | A | C | C | C | T | G | C | T | G | C | A | C | T | C | G | C | C | T | A | C | C | C | G | C | C | T | T | A | A | G | C | T | C | C | T | C | T | G | C | A | T |
| 81 | 7 | G | G | C | T | C | C | G | C | T | G | A | G | T | A | C | C | C | A | G | C | A | C | C | C | C | G | C | C | C | C | A | C | T | G | C | G | C | T | A | G | T | C | T | G | C | C | C | G | C | C | T | T | A | A | G | C | T | C | C | T | C | T | G | C | A | T |
| 82 | UPEC-33 | G | G | C | T | C | C | G | C | T | G | A | G | T | A | C | C | C | A | G | C | A | C | C | C | C | G | C | C | C | C | A | C | T | G | C | G | C | T | A | G | T | C | T | G | C | C | C | G | C | C | T | T | A | A | G | C | T | C | C | T | C | T | G | C | A | T |
| 83 | UPEC-31 | G | G | C | T | C | C | G | C | T | G | A | G | T | A | C | C | C | A | G | C | A | C | C | C | C | G | C | C | C | C | A | C | T | G | C | G | C | T | A | G | T | C | T | G | C | C | C | G | C | C | T | T | A | C | G | C | T | C | C | T | C | T | G | C | A | T |
| 84 | UPEC-119 | G | A | C | T | C | C | G | C | T | G | A | G | T | A | C | C | C | A | G | C | A | C | C | C | C | G | C | C | C | C | A | C | T | G | C | G | C | T | A | G | T | C | T | G | C | C | C | G | C | C | T | T | A | C | G | C | T | C | C | T | C | T | G | C | A | T |
| 85 | 65 | G | G | C | T | C | C | G | C | T | G | A | G | T | A | C | C | C | A | G | C | A | C | C | C | C | G | C | C | C | C | A | C | T | G | C | G | C | T | A | G | T | C | T | G | C | C | C | G | C | C | T | T | A | C | G | C | T | C | C | T | C | T | G | C | A | T |
| 86 | 88 | G | G | C | T | C | C | G | C | T | G | A | G | T | A | C | C | C | A | G | C | A | C | C | C | C | G | C | C | C | C | A | C | T | G | C | G | C | T | A | G | T | T | T | G | C | C | C | G | C | C | T | T | A | A | G | C | T | C | C | T | C | T | G | C | A | T |
| 87 | 78 | G | G | C | T | C | C | G | C | T | G | A | G | T | A | C | C | C | A | G | C | A | C | C | C | C | G | C | C | C | C | G | C | T | G | C | G | C | T | A | G | T | C | T | G | C | C | C | G | C | C | T | T | A | C | t | C | T | C | C | T | C | T | G | C | A | T |
| 88 | 82 | G | G | C | T | C | C | G | C | T | G | G | A | C | A | C | C | C | A | G | C | G | C | C | C | C | G | C | T | C | C | G | C | G | G | C | A | C | T | A | G | C | C | T | G | C | C | C | G | C | C | T | T | A | C | G | C | T | C | C | C | C | C | G | C | A | T |
| 89 | UPEC-121 | G | G | C | T | C | C | G | C | T | G | G | A | C | A | C | C | C | A | G | C | G | C | C | C | C | G | C | T | C | C | G | C | G | G | C | A | C | T | A | G | C | C | A | G | C | C | C | G | C | C | G | T | A | C | G | C | T | C | C | T | C | T | G | C | A | T |
| 90 | 81 | G | G | C | T | C | C | G | C | T | G | G | A | C | A | C | C | C | A | G | C | G | C | C | C | C | G | C | T | C | C | G | C | G | G | C | A | C | T | A | G | C | C | A | G | C | C | C | G | C | C | T | T | A | C | G | C | T | C | C | T | C | T | G | C | A | T |
| 91 | 99 | G | G | C | T | C | C | G | C | T | G | G | A | C | A | C | C | C | A | G | C | G | C | C | C | C | G | C | T | C | C | G | C | G | G | C | A | C | T | A | G | C | C | A | G | C | C | C | G | C | C | T | T | A | C | G | C | T | C | C | T | C | T | G | C | A | T |
| 92 | UPEC-104 | G | G | C | T | C | C | G | C | T | G | G | A | C | A | C | C | C | A | G | C | G | C | C | C | C | G | C | T | C | C | G | C | G | G | C | A | C | T | A | G | C | C | A | G | C | C | C | G | C | C | T | T | A | C | G | C | T | C | C | T | C | T | G | C | A | T |
| 93 | UPEC-111 | G | G | C | T | C | C | G | C | T | G | G | A | C | A | C | C | C | A | G | C | G | C | T | T | C | A | C | C | C | T | G | C | T | G | C | A | C | T | A | G | T | C | T | G | C | C | C | G | C | C | T | T | A | C | G | C | T | C | C | C | C | C | G | C | G | T |
| 94 | 93 | G | G | C | T | C | C | G | C | T | G | G | A | C | A | C | C | C | A | G | C | G | C | T | T | C | A | C | C | | T | G | C | T | G | C | A | C | T | A | G | T | C | T | G | C | C | C | G | C | C | T | T | A | C | G | C | T | C | C | C | C | C | G | C | G | T |
| 95 | UPEC-238 | G | G | C | T | C | C | G | C | T | G | A | G | T | A | C | C | C | A | G | C | A | C | C | C | C | G | C | C | C | C | A | C | T | G | C | A | C | T | C | G | C | C | T | A | C | C | C | G | C | C | T | T | A | C | G | C | T | C | C | C | C | C | G | C | G | T |
| 96 | 121 | G | G | C | T | C | C | G | C | T | G | G | A | C | G | C | C | C | A | G | C | G | C | T | T | C | G | C | T | C | C | G | C | T | G | C | A | C | T | C | G | C | C | T | G | C | C | C | G | C | C | T | T | A | C | G | C | T | C | C | C | C | C | G | T | G | T |
| 97 | UPEC-211 | G | G | C | T | C | C | T | C | T | G | G | A | C | G | C | C | C | A | G | C | G | C | T | T | C | G | C | T | C | C | G | C | T | G | C | A | C | T | C | G | C | C | T | G | C | C | C | G | C | C | T | T | A | C | G | C | T | C | C | C | C | C | G | T | G | T |
| 98 | 67 | G | G | C | T | C | C | T | C | T | G | G | A | C | G | C | C | C | A | G | C | G | C | T | T | C | G | C | T | C | C | G | C | T | G | C | A | C | T | C | G | C | C | T | G | C | C | C | G | C | C | T | T | A | C | G | C | T | C | C | C | C | C | G | T | G | T |
| | 102 | G | G | C | T | C | C | T | C | T | G | G | A | C | G | C | C | C | A | G | C | G | C | T | T | C | G | C | T | C | C | G | C | T | G | C | A | C | T | C | G | C | C | T | G | C | C | C | G | C | C | T | T | A | C | G | C | T | C | C | C | C | C | G | T | G | T |
| | 113 | G | G | C | T | C | C | T | C | T | G | G | A | C | G | C | C | C | A | G | C | G | C | T | T | C | G | C | T | C | C | G | C | T | G | C | A | C | T | C | G | C | C | T | G | C | C | C | G | C | C | T | T | A | C | G | C | T | C | C | C | C | C | G | T | G | T |
| 99 | UMN026 | G | G | C | T | C | C | G | C | T | G | G | A | C | G | C | C | C | A | G | C | G | C | T | T | C | G | C | T | C | C | G | C | T | G | C | A | C | T | C | G | C | C | T | G | C | C | C | G | C | C | T | T | A | C | G | C | T | C | C | C | C | C | G | T | G | T |
| | 84 | G | G | C | T | C | C | G | C | T | G | G | A | C | G | C | C | C | A | G | C | G | C | T | T | C | G | C | T | C | C | G | C | T | G | C | A | C | T | C | G | C | C | T | G | C | C | C | G | C | C | T | T | A | C | G | C | T | C | C | C | C | C | G | T | G | T |
| | 86 | G | G | C | T | C | C | G | C | T | G | G | A | C | G | C | C | C | A | G | C | G | C | T | T | C | G | C | T | C | C | G | C | T | G | C | A | C | T | C | G | C | C | T | G | C | C | C | G | C | C | T | T | A | C | G | C | T | C | C | C | C | C | G | T | G | T |
| | 92 | G | G | C | T | C | C | G | C | T | G | G | A | C | G | C | C | C | A | G | C | G | C | T | T | C | G | C | T | C | C | G | C | T | G | C | A | C | T | C | G | C | C | T | G | C | C | C | G | C | C | T | T | A | C | G | C | T | C | C | C | C | C | G | T | G | T |
| | 103 | G | G | C | T | C | C | G | C | T | G | G | A | C | G | C | C | C | A | G | C | G | C | T | T | C | G | C | T | C | C | G | C | T | G | C | A | C | T | C | G | C | C | T | G | C | C | C | G | C | C | T | T | A | C | G | C | T | C | C | C | C | C | G | T | G | T |
| | 104 | G | G | C | T | C | C | G | C | T | G | G | A | C | G | C | C | C | A | G | C | G | C | T | T | C | G | C | T | C | C | G | C | T | G | C | A | C | T | C | G | C | C | T | G | C | C | C | G | C | C | T | T | A | C | G | C | T | C | C | C | C | C | G | T | G | T |
| | 111 | G | G | C | T | C | C | G | C | T | G | G | A | C | G | C | C | C | A | G | C | G | C | T | T | C | G | C | T | C | C | G | C | T | G | C | A | C | T | C | G | C | C | T | G | C | C | C | G | C | C | T | T | A | C | G | C | T | C | C | C | C | C | G | T | G | T |
| | 118 | G | G | C | T | C | C | G | C | T | G | G | A | C | G | C | C | C | A | G | C | G | C | T | T | C | G | C | T | C | C | G | C | T | G | C | A | C | T | C | G | C | C | T | G | C | C | C | G | C | C | T | T | A | C | G | C | T | C | C | C | C | C | G | T | G | T |

|  |  | 1398 | 1413 | 1434 | 1435 | 1447 | 1460 | 1472 | 1484 | 1500 | 1503 | 1506 | 1509 | 1515 | 1521 | 1527 | 1555 | 1562 | 1568 | 1570 | 1584 | 1596 | 1599 | 1602 | 1607 | 1608 | 1617 | 1620 | 1638 | 1659 | 1677 | 1722 | 1723 | 1734 | 1739 | 1748 | 1765 | 1776 | 1779 | 1785 | 1791 | 1827 | 1828 | 1833 | 1835 | 1842 | 1856 | 1872 | 1890 | 1902 | 1905 | 1976 | 1980 | 1983 | 1995 | 1999 | 2019 | 2022 | 2031 | 2056 | 2067 | 2069 | 2073 | 2104 | 2105 | 2111 | 2144 |
|---|---|---|---|---|---|---|---|---|---|---|---|---|---|---|---|---|---|---|---|---|---|---|---|---|---|---|---|---|---|---|---|---|---|---|---|---|---|---|---|---|---|---|---|---|---|---|---|---|---|---|---|---|---|---|---|---|---|---|---|---|---|---|---|---|---|---|---|
| 1 | 53 | A | C | T | T | G | G | C | A | T | C | T | T | C | G | G | C | C | C | C | G | G | G | A | C | G | T | C | T | C | C | C | A | T | C | G | C | C | T | A | G | C | C | G | A | A | C | G | G | C | G | C | A | G | A | C | C | A | A | C | T | G | G | G | G | G | C |
| 2 | Di2 | A | C | T | T | G | G | C | A | T | C | T | T | C | G | G | C | C | C | C | G | G | G | A | C | G | T | C | T | C | C | C | A | T | C | G | C | C | T | A | G | C | C | G | A | A | C | G | G | C | G | C | A | G | A | C | C | A | A | C | T | G | G | G | G | G | C |
|  | Di14 | A | C | T | T | G | G | C | A | T | C | T | T | C | G | G | C | C | C | C | G | G | G | A | C | G | T | C | T | C | C | C | A | T | C | G | C | C | T | A | G | C | C | G | A | A | C | G | G | C | G | C | A | G | A | C | C | A | A | C | T | G | G | G | G | G | C |
|  | CFT073 | A | C | T | T | G | G | C | A | T | C | T | T | C | G | G | C | C | C | C | G | G | G | A | C | G | T | C | T | C | C | C | A | T | C | G | C | C | T | A | G | C | C | G | A | A | C | G | G | C | G | C | A | G | A | C | C | A | A | C | T | G | G | G | G | G | C |
| 3 | 49 | A | C | T | T | G | G | C | A | T | C | T | T | C | G | G | C | C | C | C | G | G | G | A | C | G | T | C | T | C | C | C | A | T | C | G | C | C | T | A | G | C | C | G | A | A | C | G | G | C | G | C | A | G | A | C | C | A | A | C | T | G | G | G | G | G | C |
|  | 87 | A | C | T | T | G | G | C | A | T | C | T | T | C | G | G | C | C | C | C | G | G | G | A | C | G | T | C | T | C | C | C | A | T | C | G | C | C | T | A | G | C | C | G | A | A | C | G | G | C | G | C | A | G | A | C | C | A | A | C | T | G | G | G | G | G | C |
|  | 91 | A | C | T | T | G | G | C | A | T | C | T | T | C | G | G | C | C | C | C | G | G | G | A | C | G | T | C | T | C | C | C | A | T | C | G | C | C | T | A | G | C | C | G | A | A | C | G | G | C | G | C | A | G | A | C | C | A | A | C | T | G | G | G | G | G | C |
|  | 95 | A | C | T | T | G | G | C | A | T | C | T | T | C | G | G | C | C | C | C | G | G | G | A | C | G | T | C | T | C | C | C | A | T | C | G | C | C | T | A | G | C | C | G | A | A | C | G | G | C | G | C | A | G | A | C | C | A | A | C | T | G | G | G | G | G | C |
|  | 72 | A | C | T | T | G | G | C | A | T | C | T | T | C | G | G | C | C | C | C | G | G | G | A | C | G | T | C | T | C | C | C | A | T | C | G | C | C | T | A | G | C | C | G | A | A | C | G | G | C | G | C | A | G | A | C | C | A | A | C | T | G | G | G | G | G | C |
| 4 | UPEC-79 | A | C | T | T | G | G | C | A | T | C | T | T | C | G | G | C | C | T | C | G | G | G | A | C | G | T | C | T | C | C | C | A | T | T | G | C | C | T | A | G | C | C | G | A | A | C | G | G | C | G | C | A | G | A | C | C | A | A | C | T | G | G | G | G | G | C |
| 5 | 114 | A | C | T | T | G | G | C | A | T | C | T | T | C | G | G | C | C | C | C | G | G | G | A | C | G | T | C | T | C | C | C | A | T | T | G | C | C | T | A | G | C | C | G | A | A | C | G | G | C | G | C | A | G | A | C | C | A | A | C | T | G | G | G | G | G | C |
| 6 | ABU83972 | A | C | T | T | A | G | C | A | T | C | T | T | C | G | G | C | C | C | C | G | G | G | A | C | G | T | C | T | C | C | C | A | T | T | G | C | C | T | A | G | C | C | G | A | A | C | G | G | C | G | C | A | G | A | C | C | A | A | C | T | G | G | G | G | G | C |
| 7 | 94 | A | C | T | T | G | G | C | A | T | C | T | T | C | G | G | C | C | C | C | G | G | G | A | C | G | T | C | T | C | C | C | A | T | T | G | C | C | T | A | G | C | C | G | A | A | C | G | G | C | G | C | A | G | A | C | C | A | A | C | T | G | G | G | G | G | C |
| 8 | UPEC-116 | A | C | T | T | G | G | C | A | T | C | T | T | C | G | G | C | C | C | C | G | G | G | A | C | G | T | C | T | C | C | C | A | T | T | G | C | C | T | A | G | C | C | G | A | A | C | G | G | C | G | C | A | G | A | C | C | A | A | C | T | G | G | G | G | G | C |
| 9 | UPEC-180 | A | C | T | T | G | G | C | A | T | C | T | T | C | G | G | C | C | C | C | G | G | G | A | C | G | T | C | T | C | C | C | A | T | T | G | C | C | T | A | G | C | C | G | A | A | C | G | G | C | G | C | A | G | A | C | C | A | A | C | T | G | G | G | G | G | C |
| 10 | UPEC-100 | A | C | T | T | G | G | C | A | T | C | T | T | C | G | G | C | C | C | C | G | G | G | A | C | G | T | C | T | C | C | C | A | T | T | G | C | C | T | A | G | C | C | G | A | A | C | G | G | C | G | C | A | G | A | C | C | A | A | C | T | G | G | A | G | G | C |
| 11 | UPEC-112 | A | C | T | T | G | G | C | A | T | C | T | T | C | G | G | C | C | C | C | G | G | G | A | C | G | T | C | T | C | C | C | A | T | T | G | C | C | T | A | G | C | C | G | A | C | C | G | G | C | G | C | A | G | A | C | C | A | A | C | T | G | G | G | G | G | C |
| 12 | 85 | A | C | T | T | G | G | C | A | T | C | T | T | C | G | G | C | C | C | C | G | G | G | A | C | G | T | C | T | C | C | C | A | C | T | G | C | C | T | A | G | C | C | G | A | C | C | G | G | C | G | C | A | G | A | C | C | A | A | C | T | G | G | G | G | G | C |
|  | UPEC-115 | A | C | T | T | G | G | C | A | T | C | T | T | C | G | G | C | C | C | C | G | G | G | A | C | G | T | C | T | C | C | C | A | C | T | G | C | C | T | A | G | C | C | G | A | C | C | G | G | C | G | C | A | G | A | C | C | A | A | C | T | G | G | G | G | G | C |
| 13 | UTI89 | A | C | T | G | G | G | C | A | T | C | T | T | C | G | G | C | C | C | C | G | G | G | A | C | G | T | C | T | C | C | C | A | C | T | G | C | C | T | A | G | C | C | G | A | C | C | G | G | C | G | C | A | G | A | C | C | A | A | C | T | G | G | G | G | G | C |
| 14 | UPEC-153 | G | C | T | T | G | G | C | A | T | C | T | T | C | G | G | C | C | C | C | T | T | C | G | C | G | T | T | T | C | T | C | A | T | T | G | C | C | T | A | G | C | C | G | A | C | C | G | G | C | G | C | A | G | A | C | C | A | A | C | T | G | G | G | G | G | C |
| 15 | 101 | G | C | T | T | G | G | C | A | T | C | T | T | C | G | G | C | C | C | C | T | G | G | A | C | G | A | C | T | C | C | C | A | C | T | G | C | C | T | A | G | C | C | G | A | C | C | G | G | C | G | C | A | G | A | C | C | A | A | C | T | G | G | G | G | G | C |
| 16 | 122 | G | C | T | T | G | G | T | A | T | C | T | T | C | G | G | C | C | C | C | T | G | G | A | C | G | A | C | T | C | C | C | A | C | T | G | C | C | T | A | G | C | C | G | A | C | C | G | G | C | G | C | A | G | A | C | C | A | A | C | T | G | G | G | G | G | C |
| 17 | 11 | G | C | T | T | G | G | C | A | T | C | T | T | C | G | G | C | C | C | C | T | G | G | A | C | G | A | C | T | C | C | C | A | C | T | G | C | C | T | A | G | C | C | G | A | C | C | G | G | C | G | C | A | G | A | C | C | A | A | C | T | G | G | G | G | G | C |
| 18 | 97 | G | C | T | T | A | G | C | A | T | C | T | T | C | G | G | C | C | C | C | T | G | G | A | C | G | A | C | T | C | C | C | A | C | T | G | C | C | T | A | G | C | C | G | A | C | C | G | G | C | G | C | A | G | A | C | C | A | A | C | T | G | G | G | G | G | C |
| 19 | UPEC-195 | G | C | T | T | G | G | C | A | T | C | T | T | C | G | G | C | C | C | C | T | G | G | A | C | G | A | C | T | C | C | C | A | C | T | G | C | C | T | A | G | C | C | G | A | C | C | G | G | C | G | C | A | G | A | C | C | A | A | C | T | G | G | G | G | G | C |
| 20 | UPEC-150 | G | C | T | T | G | G | C | A | T | C | T | T | C | G | G | C | C | C | C | T | G | G | A | C | G | A | C | T | C | C | C | A | C | T | G | C | C | T | A | G | C | C | G | A | C | C | G | G | C | G | C | A | G | A | C | C | A | A | C | T | G | G | G | G | G | C |
| 21 | UPEC-101 | G | C | T | G | G | G | C | A | T | C | T | T | C | G | G | C | C | C | C | T | G | G | A | C | G | A | C | T | C | C | C | A | C | T | G | C | C | T | A | G | C | T | G | A | C | C | G | G | C | G | C | A | G | A | C | C | A | A | C | T | G | G | G | G | G | C |
| 22 | UPEC-59 | G | C | T | T | G | G | C | A | T | C | T | T | C | G | G | C | C | C | C | T | G | G | A | C | G | A | C | T | C | C | C | A | C | T | G | C | C | T | A | G | C | T | G | A | C | C | G | G | C | G | C | A | G | A | C | C | A | A | C | T | G | G | G | G | G | C |
| 23 | 119 | G | C | T | T | G | G | T | A | T | C | T | T | C | G | G | C | C | C | C | T | G | G | A | C | G | A | C | T | C | C | C | A | C | C | G | C | C | T | A | G | C | C | G | A | C | C | G | G | C | G | C | A | G | A | C | C | A | A | C | T | G | G | G | G | G | C |
| 24 | 8 | G | C | T | T | G | G | C | A | T | C | T | T | C | G | G | C | C | C | C | T | G | G | A | C | G | A | C | T | C | C | C | A | C | T | G | C | C | T | A | G | C | C | G | A | C | C | G | G | C | G | C | A | G | A | C | C | A | A | C | T | G | G | G | G | G | C |
| 25 | 1 | G | C | T | T | A | G | C | A | T | C | T | T | C | G | G | C | C | C | C | T | G | G | A | C | G | A | C | T | C | C | C | A | C | T | G | C | C | T | A | G | C | C | G | A | C | C | G | G | C | G | C | A | G | A | C | C | A | A | C | T | G | G | G | G | G | C |
| 26 | 58 | G | C | T | T | G | G | C | A | T | C | T | T | C | G | G | C | C | C | C | T | G | G | A | C | G | A | C | T | C | C | C | A | C | T | G | C | C | T | A | G | C | C | G | A | C | C | G | G | C | G | C | A | G | A | C | C | A | A | C | T | G | G | G | G | G | C |
|  | 71 | G | C | T | T | G | G | C | A | T | C | T | T | C | G | G | C | C | C | C | T | G | G | A | C | G | A | C | T | C | C | C | A | C | T | G | C | C | T | A | G | C | C | G | A | C | C | G | G | C | G | C | A | G | A | C | C | A | A | C | T | G | G | G | G | G | C |
| 27 | 68 | G | C | T | T | G | G | C | A | T | C | T | T | C | G | G | C | C | C | C | T | G | G | A | C | G | A | C | T | C | C | C | A | C | T | G | C | C | T | A | G | C | C | G | A | C | C | G | G | C | G | C | A | G | A | C | C | A | A | C | T | G | G | G | G | G | C |
| 28 | 107 | G | C | T | T | G | G | C | A | T | C | T | T | C | G | G | C | C | C | C | G | G | G | A | C | G | T | C | T | C | C | C | T | T | T | G | C | C | T | A | G | C | C | G | A | A | C | G | G | C | G | C | A | G | A | C | C | A | A | C | T | G | G | G | G | G | C |
| 29 | UPEC-179 | G | C | T | T | A | G | C | A | T | C | T | T | C | G | G | C | C | C | C | T | G | G | A | C | G | A | C | T | C | C | C | A | C | T | G | C | C | T | A | G | C | C | G | A | A | C | G | G | C | G | C | A | G | A | C | C | A | A | C | T | G | G | G | G | G | C |
| 30 | UPEC-78 | G | C | T | T | G | G | C | A | T | C | T | T | C | G | G | C | C | C | C | T | G | G | A | C | G | A | C | T | C | C | C | A | C | T | G | C | C | T | A | G | C | C | G | A | A | C | G | G | C | G | C | A | G | A | C | C | A | A | C | T | G | G | G | G | G | C |
| 31 | UPEC-274 | G | C | T | T | G | G | C | A | T | C | T | T | C | G | G | C | C | C | C | T | G | G | A | C | G | A | C | T | C | C | C | A | C | T | G | C | C | T | A | G | C | C | G | A | C | C | G | G | C | G | C | A | G | A | C | C | A | A | C | T | G | G | G | G | G | C |
| 32 | UPEC-243 | G | C | T | T | G | G | C | A | T | C | T | T | C | G | G | C | C | C | C | G | G | G | A | C | G | T | C | T | C | C | C | A | C | T | G | T | C | T | A | G | C | C | G | A | C | C | G | G | C | G | C | A | G | A | C | C | A | A | C | T | G | G | G | G | G | C |

| | | | | | | | | | | | | | | | | | | | | | | | | | | | | | | | | | | | | | | | | | | | | | | | | | | | | | | | | | | | | | | | | | | | |
|---|---|---|---|---|---|---|---|---|---|---|---|---|---|---|---|---|---|---|---|---|---|---|---|---|---|---|---|---|---|---|---|---|---|---|---|---|---|---|---|---|---|---|---|---|---|---|---|---|---|---|---|---|---|---|---|---|---|---|---|---|---|---|---|---|---|---|---|
| 33 | 5 | G | C | T | T | G | G | C | A | T | C | T | T | C | G | G | C | C | C | C | T | G | G | A | C | G | T | C | T | C | C | C | A | T | T | G | T | C | T | A | G | C | C | G | A | C | C | G | G | C | G | C | A | G | A | C | C | A | A | C | T | G | G | G | G | G | C |
| 34 | 70 | G | C | T | T | G | G | C | A | T | C | T | T | C | G | G | C | C | C | C | T | G | G | A | C | G | T | C | T | C | C | C | A | T | T | G | T | C | T | A | G | C | C | G | A | C | C | G | G | C | G | C | A | G | A | C | C | A | A | C | T | G | G | G | G | G | C |
| | 115 | G | C | T | T | G | G | C | A | T | C | T | T | C | G | G | C | C | C | C | T | G | G | A | C | G | T | C | T | C | C | C | A | T | T | G | T | C | T | A | G | C | C | G | A | C | C | G | G | C | G | C | A | G | A | C | C | A | A | C | T | G | G | G | G | G | C |
| 35 | 536 | G | C | T | T | G | G | C | A | T | C | T | T | C | G | G | C | C | C | C | T | G | G | A | C | G | T | C | T | C | C | C | A | T | T | G | T | C | T | A | G | C | C | G | A | C | C | G | G | C | G | C | A | G | A | C | C | A | A | C | T | G | G | G | G | G | C |
| 36 | F11 | G | C | T | T | G | G | T | A | T | C | T | T | C | G | G | C | C | C | C | T | G | G | A | C | G | T | C | T | C | C | C | A | T | T | G | T | C | T | A | G | C | C | G | A | C | C | G | G | C | G | C | A | G | A | C | C | A | A | C | T | G | G | G | G | G | C |
| 37 | UPEC-38 | G | T | C | T | G | A | C | A | T | C | T | T | C | G | G | C | C | C | C | T | G | G | A | C | G | T | C | T | C | C | C | A | C | T | G | C | C | T | A | G | C | C | G | A | C | C | G | G | T | G | C | A | G | A | C | C | A | A | C | T | G | G | G | G | G | C |
| 38 | UPEC-58 | G | T | C | T | G | A | C | A | T | C | T | T | C | G | G | C | C | C | C | T | G | G | A | C | G | T | C | T | C | C | C | A | C | T | G | C | C | T | A | G | C | C | G | A | C | C | A | G | A | G | T | A | G | A | C | C | A | A | C | T | G | A | G | G | G | C |
| 39 | UPEC-219 | G | T | C | T | G | A | C | A | T | C | T | T | C | G | G | C | C | C | C | T | G | G | A | C | G | T | C | T | C | C | C | A | C | T | G | C | C | T | A | G | C | C | G | A | C | C | A | G | C | G | C | A | G | A | C | C | A | A | C | T | G | G | G | G | G | C |
| 40 | 69 | G | T | C | T | G | A | C | A | T | C | T | T | C | G | G | C | C | C | C | T | G | G | A | C | G | T | C | A | C | C | T | A | T | T | G | C | C | T | A | G | C | C | G | A | C | C | G | G | A | G | T | A | G | A | C | C | A | A | C | C | G | A | G | G | G | C |
| | 110 | G | T | C | T | G | A | C | A | T | C | T | T | C | G | G | C | C | C | C | T | G | G | A | C | G | T | C | A | C | C | T | A | T | T | G | C | C | T | A | G | C | C | G | A | C | C | G | G | A | G | T | A | G | A | C | C | A | A | C | C | G | A | G | G | G | C |
| 41 | UPEC-266 | G | T | C | T | G | G | C | G | A | T | T | C | T | G | G | C | C | C | C | T | G | G | A | C | G | A | C | T | C | C | C | A | C | T | G | T | C | T | A | G | C | T | G | A | C | C | G | G | T | A | C | A | G | A | C | C | A | A | C | T | G | G | G | G | G | C |
| 42 | UPEC-202 | G | T | C | T | G | G | C | G | A | T | T | C | T | G | G | C | C | C | C | T | G | C | G | C | G | T | T | T | C | T | C | A | C | T | G | C | C | T | A | G | C | C | G | A | C | C | G | G | C | G | C | A | G | A | C | C | A | A | C | T | G | G | G | T | G | T |
| 43 | UPEC-233 | G | T | C | T | G | A | C | G | A | T | G | C | T | G | G | C | C | C | C | T | G | C | G | T | G | T | C | T | C | C | C | A | C | T | G | C | C | T | A | G | C | C | G | A | C | C | G | G | T | A | C | A | G | A | C | C | A | A | C | T | C | G | G | G | G | C |
| 44 | 109 | G | T | C | T | G | A | C | G | A | T | G | C | T | G | G | C | C | C | C | T | G | C | G | T | G | T | C | T | C | C | C | A | C | T | G | C | C | T | A | G | C | C | G | A | C | C | G | G | T | A | C | A | G | A | C | C | A | A | C | T | G | G | G | G | G | C |
| 45 | UPEC-236 | G | C | C | T | G | A | C | G | A | T | T | C | C | G | C | C | C | C | C | T | G | C | G | C | G | T | C | T | C | C | C | A | T | T | G | C | C | T | A | A | C | C | G | A | C | C | G | G | A | G | C | A | A | A | C | C | A | A | A | T | G | G | G | G | G | C |
| 46 | NA114 | G | C | C | T | G | A | C | G | A | T | G | T | C | G | G | C | C | C | C | T | G | T | G | C | G | T | C | T | C | C | T | A | C | T | A | C | C | T | A | A | C | C | G | A | C | C | G | G | A | G | C | A | G | A | C | C | A | A | C | T | G | G | G | G | G | C |
| | 108 | G | C | C | T | G | A | C | G | A | T | G | T | C | G | G | C | C | C | C | T | G | T | G | C | G | T | C | T | C | C | T | A | C | T | A | C | C | T | A | A | C | C | G | A | C | C | G | G | A | G | C | A | G | A | C | C | A | A | C | T | G | G | G | G | G | C |
| | Ec958 | G | C | C | T | G | A | C | G | A | T | G | T | C | G | G | C | C | C | C | T | G | T | G | C | G | T | C | T | C | C | T | A | C | T | A | C | C | T | A | A | C | C | G | A | C | C | G | G | A | G | C | A | G | A | C | C | A | A | C | T | G | G | G | G | G | C |
| 47 | 66 | G | C | C | T | G | A | C | G | A | T | G | T | C | G | G | C | C | C | C | T | G | T | G | C | G | T | C | T | C | C | T | A | C | T | A | C | C | T | A | A | C | C | G | A | C | C | G | G | A | G | C | A | G | A | C | C | A | A | C | C | G | G | G | G | G | C |
| | 105 | G | C | C | T | G | A | C | G | A | T | G | T | C | G | G | C | C | C | C | T | G | T | G | C | G | T | C | T | C | C | T | A | C | T | A | C | C | T | A | A | C | C | G | A | C | C | G | G | A | G | C | A | G | A | C | C | A | A | C | C | G | G | G | G | G | C |
| 48 | 112 | G | C | C | T | G | A | C | G | A | T | G | T | C | G | G | C | C | C | C | T | G | T | G | C | G | T | C | T | C | C | T | A | C | T | A | C | C | T | A | A | C | C | G | A | C | C | G | G | A | G | C | A | G | A | C | C | A | A | C | T | G | G | G | G | G | C |
| 49 | 98 | G | C | C | T | G | A | C | G | A | T | T | C | C | G | C | C | C | C | C | T | G | C | G | C | G | T | C | T | C | C | T | A | C | T | G | C | C | T | A | A | C | C | G | A | C | C | G | G | A | G | T | A | G | A | C | C | A | A | C | T | G | A | G | G | G | C |
| 50 | 74 | G | C | C | T | G | A | C | G | A | T | T | C | C | G | C | C | C | C | C | T | G | C | G | C | G | T | C | T | C | C | T | A | C | T | G | C | C | T | A | A | C | C | G | A | C | C | G | G | T | A | C | A | G | G | C | T | A | A | C | T | G | G | G | G | G | C |
| 51 | 60 | G | C | C | T | G | A | C | G | A | T | T | C | C | G | C | C | C | C | C | T | G | C | G | C | G | T | C | T | C | C | T | A | C | T | G | C | C | T | A | A | C | C | G | A | C | C | G | G | T | A | C | A | G | G | C | T | A | A | C | T | G | G | G | G | G | C |
| 52 | 47 | G | C | C | T | G | A | C | G | A | T | T | C | C | G | C | C | C | C | C | T | G | C | G | C | G | T | C | T | C | C | T | A | C | T | G | C | C | T | A | A | C | C | G | A | C | C | G | G | T | A | C | A | G | G | C | T | A | A | C | T | G | G | G | G | G | C |
| 53 | UPEC-149 | G | C | C | T | G | A | C | G | A | T | T | C | C | G | C | C | T | C | C | T | G | C | G | C | G | T | C | T | C | C | T | A | C | T | G | C | C | T | A | A | C | C | G | A | C | C | G | G | T | A | C | A | G | G | C | T | A | A | C | T | G | G | G | G | G | C |
| 54 | 57 | G | C | C | T | G | A | C | G | A | T | T | C | C | G | C | C | C | C | C | T | G | C | G | C | G | T | C | T | C | C | T | A | C | T | G | C | C | T | A | A | C | C | G | A | C | C | G | G | T | A | C | A | G | G | C | T | A | A | C | T | G | G | G | G | G | C |
| | 59 | G | C | C | T | G | A | C | G | A | T | T | C | C | G | C | C | C | C | C | T | G | C | G | C | G | T | C | T | C | C | T | A | C | T | G | C | C | T | A | A | C | C | G | A | C | C | G | G | T | A | C | A | G | G | C | T | A | A | C | T | G | G | G | G | G | C |
| | 77 | G | C | C | T | G | A | C | G | A | T | T | C | C | G | C | C | C | C | C | T | G | C | G | C | G | T | C | T | C | C | T | A | C | T | G | C | C | T | A | A | C | C | G | A | C | C | G | G | T | A | C | A | G | G | C | T | A | A | C | T | G | G | G | G | G | C |
| | 90 | G | C | C | T | G | A | C | G | A | T | T | C | C | G | C | C | C | C | C | T | G | C | G | C | G | T | C | T | C | C | T | A | C | T | G | C | C | T | A | A | C | C | G | A | C | C | G | G | T | A | C | A | G | G | C | T | A | A | C | T | G | G | G | G | G | C |
| | UPEC-258 | G | C | C | T | G | A | C | G | A | T | T | C | C | G | C | C | C | C | C | T | G | C | G | C | G | T | C | T | C | C | T | A | C | T | G | C | C | T | A | A | C | C | G | A | C | C | G | G | T | A | C | A | G | G | C | T | A | A | C | T | G | G | G | G | G | C |
| 55 | 75 | G | C | C | T | G | A | C | G | A | T | G | C | C | G | C | C | C | C | C | T | G | C | G | C | G | T | C | A | C | C | T | A | T | T | G | C | T | C | G | G | C | C | G | A | C | C | G | C | A | A | C | A | G | A | C | C | A | T | C | T | G | G | G | G | G | C |
| 56 | 62 | G | C | T | T | G | A | C | G | A | T | G | C | T | G | C | C | C | C | C | T | G | C | G | C | G | T | T | T | T | T | C | A | C | T | G | C | C | T | A | G | C | C | G | A | C | C | G | C | C | G | C | A | G | A | C | C | A | A | C | T | G | G | G | T | G | C |
| | UPEC-137 | G | C | T | T | G | A | C | G | A | T | G | C | T | G | C | C | C | C | C | T | G | C | G | C | G | T | T | T | T | T | C | A | C | T | G | C | C | T | A | G | C | C | G | A | C | C | G | C | C | G | C | A | G | A | C | C | A | A | C | T | G | G | G | T | G | C |
| 57 | UPEC-213 | G | C | T | T | G | A | C | G | A | T | G | C | T | A | C | C | C | C | C | T | G | C | G | C | G | T | C | T | C | C | T | A | C | T | G | C | T | C | G | G | C | C | G | T | C | C | G | C | A | G | C | A | G | A | C | C | A | A | C | T | G | G | G | T | G | T |
| 58 | 4 | G | C | T | T | G | A | C | G | A | T | G | C | T | A | C | C | C | C | C | T | G | C | G | C | G | T | C | T | C | C | T | A | C | T | G | C | T | C | G | G | C | C | G | T | C | C | G | G | A | G | C | A | G | A | C | C | T | A | C | T | G | G | G | G | G | C |
| 59 | 3 | G | C | C | T | G | A | C | G | A | T | T | C | C | G | C | C | C | C | C | T | G | C | G | C | G | T | C | T | C | C | C | A | T | T | G | C | C | T | A | A | C | C | G | A | C | C | G | G | A | G | C | A | A | A | C | C | A | A | A | T | G | G | G | G | G | C |
| 60 | UPEC-103 | G | C | C | T | G | A | C | G | A | T | G | T | C | G | G | C | C | C | C | T | G | T | G | C | G | T | C | T | C | C | T | A | C | T | G | C | C | T | A | A | C | C | G | A | C | C | G | G | A | G | C | A | G | A | C | C | A | C | C | T | G | G | G | G | G | C |
| 61 | UPEC-200 | G | C | C | T | G | A | C | G | A | T | G | T | C | G | G | C | C | C | C | T | G | T | G | C | G | T | C | T | C | C | T | A | C | T | G | C | C | T | A | A | C | C | G | A | C | C | G | G | A | G | C | A | A | A | C | C | A | A | C | T | G | G | G | G | G | C |
| 62 | 54 | G | T | C | T | G | A | C | G | A | T | G | C | T | G | G | C | C | C | C | T | G | C | G | T | G | T | C | T | C | C | C | A | C | T | G | C | C | T | A | G | C | C | G | A | C | C | G | G | T | A | C | A | G | A | C | C | A | A | C | T | C | G | G | T | G | C |
| | 80 | G | T | C | T | G | A | C | G | A | T | G | C | T | G | G | C | C | C | C | T | G | C | G | T | G | T | C | T | C | C | C | A | C | T | G | C | C | T | A | G | C | C | G | A | C | C | G | G | T | A | C | A | G | A | C | C | A | A | C | T | C | G | G | T | G | C |
| 63 | 100 | G | T | C | T | G | A | C | G | A | T | G | C | T | G | G | C | C | C | C | T | G | C | G | T | G | T | C | T | C | C | C | A | C | T | G | C | C | T | A | G | C | C | G | A | C | C | G | G | T | A | C | A | G | A | C | C | A | A | C | T | C | G | G | T | G | C |
| 64 | 14 | G | T | C | T | G | A | C | G | A | T | G | C | T | G | G | C | C | C | C | T | G | C | G | T | G | T | C | T | C | C | C | A | C | T | G | C | C | T | A | G | C | C | G | A | C | C | G | G | T | A | C | A | G | A | C | C | A | A | C | T | C | G | G | T | G | C |
| 65 | 123 | G | T | C | T | G | A | C | G | A | T | G | C | T | G | G | C | C | C | C | T | G | C | G | T | G | T | C | T | C | C | C | A | C | T | G | C | C | T | A | G | C | C | G | A | C | C | G | G | T | A | C | A | G | A | C | C | A | A | C | T | G | G | G | G | G | C |
| 66 | 83 | G | T | C | T | G | A | C | G | A | T | G | C | T | G | G | C | C | C | C | T | G | C | G | T | G | T | C | T | C | C | C | A | C | T | G | C | C | T | A | G | C | C | G | A | C | C | G | G | T | A | C | A | G | A | C | C | A | A | C | T | C | G | G | G | G | C |

| | | | | | | | | | | | | | | | | | | | | | | | | | | | | | | | | | | | | | | | | | | | | | | | | | | | | | | | | | | | | | | | | | | | |
|---|---|---|---|---|---|---|---|---|---|---|---|---|---|---|---|---|---|---|---|---|---|---|---|---|---|---|---|---|---|---|---|---|---|---|---|---|---|---|---|---|---|---|---|---|---|---|---|---|---|---|---|---|---|---|---|---|---|---|---|---|---|---|---|---|---|---|---|
| 67 | 64 | G | T | C | T | G | A | C | G | A | T | G | C | T | G | G | C | C | C | C | T | G | C | G | T | G | T | C | T | C | C | C | A | C | T | G | C | C | T | A | G | C | C | G | A | C | C | G | G | T | A | C | A | G | A | C | C | A | A | C | T | C | G | G | G | G | C |
| | UPEC-205 | G | T | C | T | G | A | C | G | A | T | G | C | T | G | G | C | C | C | C | T | G | C | G | T | G | T | C | T | C | C | C | A | C | T | G | C | C | T | A | G | C | C | G | A | C | C | G | G | T | A | C | A | G | A | C | C | A | A | C | T | C | G | G | G | G | C |
| 68 | UPEC-132 | G | T | C | T | G | A | C | G | A | T | G | C | T | G | G | C | C | C | C | T | G | C | G | T | G | T | C | T | C | C | C | A | C | T | G | C | C | T | A | G | C | C | G | A | C | C | G | G | T | A | C | A | G | A | C | C | A | A | C | T | C | G | G | G | G | C |
| 69 | 120 | G | T | C | T | G | A | C | G | A | T | G | C | T | G | G | C | C | C | C | T | G | C | G | T | G | T | C | T | C | C | C | A | C | T | G | C | C | T | A | G | C | C | G | A | C | C | G | G | T | A | C | A | G | A | C | C | A | A | C | T | C | G | G | G | G | C |
| 70 | UPEC-105 | G | C | C | T | G | A | C | G | A | T | T | C | C | G | G | C | C | C | C | T | T | C | G | C | G | T | C | A | C | C | T | A | T | T | G | C | T | C | G | G | C | C | G | A | C | C | G | C | A | A | C | G | G | A | C | C | A | A | C | T | G | G | G | G | G | C |
| 71 | UPEC-175 | G | C | C | T | G | A | C | G | A | T | T | C | C | G | C | C | C | C | C | T | G | C | G | C | G | T | C | T | C | C | T | A | C | T | G | C | C | T | A | A | C | C | G | A | C | C | G | G | T | A | C | A | G | G | C | T | A | A | C | T | G | G | G | G | G | C |
| 72 | UPEC-133 | G | C | C | T | G | A | C | G | A | T | T | C | C | G | G | C | C | C | C | T | T | C | G | C | G | T | C | A | C | C | T | A | T | T | G | C | T | C | G | G | C | C | G | T | C | C | G | G | A | G | C | A | G | A | C | C | A | A | C | T | G | G | G | G | G | C |
| 73 | 96 | G | C | T | T | G | A | C | G | A | T | G | C | T | G | C | C | C | C | C | T | G | C | G | C | G | T | C | T | C | C | T | A | C | T | G | C | C | T | A | G | C | C | G | A | C | C | G | C | A | A | C | G | G | A | C | C | A | A | C | T | G | G | G | G | G | C |
| 74 | 89 | G | C | C | T | G | A | C | G | A | T | T | C | C | G | C | C | C | C | C | T | G | C | G | C | G | T | C | T | C | C | T | A | C | T | G | C | C | T | A | A | C | C | G | A | C | C | G | G | T | A | C | A | G | G | C | T | A | A | C | T | G | G | G | T | G | C |
| | UPEC-127 | G | C | C | T | G | A | C | G | A | T | T | C | C | G | C | C | C | C | C | T | G | C | G | C | G | T | C | T | C | C | T | A | C | T | G | C | C | T | A | A | C | C | G | A | C | C | G | G | T | A | C | A | G | G | C | T | A | A | C | T | G | G | G | T | G | C |
| 75 | 55 | G | C | C | T | G | A | C | G | A | T | T | C | C | G | C | C | C | C | C | T | G | C | G | C | G | T | C | T | C | C | T | A | C | T | G | C | C | T | A | A | C | C | G | A | C | C | G | G | T | A | C | A | G | G | C | T | A | A | C | T | G | G | G | T | G | C |
| 76 | IAI39 | G | C | C | T | T | A | C | G | A | T | G | C | C | G | C | C | C | C | C | T | G | C | G | C | A | T | C | T | C | C | T | A | T | T | G | C | C | T | A | G | C | C | G | A | C | C | G | G | T | A | C | A | G | A | C | T | A | A | C | T | G | G | G | G | G | C |
| 77 | 124 | G | C | C | T | T | A | C | G | A | T | G | C | C | G | C | C | C | C | C | T | G | C | G | C | A | T | C | T | C | C | T | A | T | T | G | C | C | T | A | G | C | C | G | A | C | C | G | G | T | A | C | A | G | A | C | T | A | A | C | T | G | G | G | G | G | C |
| 78 | 76 | G | C | C | T | G | A | C | G | A | T | G | C | C | G | C | C | C | C | C | T | G | C | G | C | G | T | C | T | C | C | T | A | T | T | G | C | C | T | A | A | C | C | A | A | C | C | G | C | A | G | C | A | G | A | C | C | T | A | C | T | G | G | G | G | G | C |
| | UPEC-146 | G | C | C | T | G | A | C | G | A | T | G | C | C | G | C | C | C | C | C | T | G | C | G | C | G | T | C | T | C | C | T | A | T | T | G | C | C | T | A | A | C | C | A | A | C | C | G | C | A | G | C | A | G | A | C | C | T | A | C | T | G | G | G | G | G | C |
| 79 | 79 | G | C | C | T | G | A | C | G | A | T | G | C | C | G | G | C | C | C | C | T | G | C | G | C | G | T | C | T | C | C | T | A | C | T | G | C | C | T | A | A | C | C | G | A | C | C | G | G | A | G | C | A | G | A | C | C | A | A | C | T | G | G | G | G | G | C |
| 80 | UPEC-130 | G | C | C | T | G | A | C | G | A | T | G | C | C | G | C | C | C | C | C | T | G | C | G | C | A | T | C | T | C | C | T | A | T | T | G | C | C | T | A | G | C | C | G | A | C | C | G | G | T | A | C | A | G | A | C | T | A | A | C | T | G | G | G | G | A | C |
| 81 | 7 | G | C | C | T | G | A | T | G | A | T | G | C | C | G | G | C | C | C | C | T | G | C | G | C | G | T | C | T | C | C | T | A | C | T | G | C | C | T | A | A | C | C | G | A | C | C | G | G | A | G | C | A | G | A | C | C | A | A | C | T | G | G | G | G | G | C |
| 82 | UPEC-33 | G | C | C | T | G | A | C | G | A | T | G | C | C | G | G | C | C | C | C | T | G | C | G | C | G | T | C | T | C | C | T | A | C | T | G | C | C | T | A | A | C | C | G | A | C | C | G | G | A | G | C | A | G | A | C | C | A | A | C | T | G | G | G | G | G | C |
| 83 | UPEC-31 | G | C | C | T | G | A | C | G | A | T | G | C | C | G | C | C | C | C | C | T | G | C | G | C | G | T | C | T | C | C | T | A | T | T | G | C | C | T | A | A | C | C | G | A | C | C | G | G | A | G | C | A | G | A | C | C | T | A | C | T | G | G | G | G | G | C |
| 84 | UPEC-119 | G | C | C | T | G | A | C | G | A | T | G | C | C | G | G | C | C | C | G | T | G | C | G | C | G | T | C | T | C | C | T | A | C | T | G | C | C | T | A | A | C | C | G | A | C | C | G | G | A | G | C | A | G | A | C | C | A | A | C | T | G | G | G | G | G | C |
| 85 | 65 | G | C | C | T | G | A | C | G | A | T | G | C | C | G | G | C | C | C | C | T | G | C | G | C | G | T | C | T | C | C | T | A | C | T | G | C | C | T | A | A | C | C | G | A | C | C | G | G | A | G | C | A | G | A | C | C | A | A | C | T | G | G | G | G | G | C |
| 86 | 88 | G | C | C | T | G | A | C | G | A | T | G | C | C | G | C | C | C | C | C | T | G | C | G | C | G | T | C | T | C | C | T | A | C | T | G | C | C | T | A | A | C | C | G | A | C | C | G | G | T | A | C | A | G | A | C | C | A | A | C | T | G | G | G | G | A | C |
| 87 | 78 | G | C | C | T | G | A | C | G | A | T | G | C | C | G | G | C | C | C | C | T | G | C | G | C | G | T | C | T | C | C | T | A | C | T | G | C | C | T | A | A | C | C | G | A | C | C | G | G | A | G | C | A | G | A | C | C | A | A | C | T | G | G | G | G | G | C |
| 88 | 82 | G | C | C | T | G | A | C | G | A | T | G | C | C | G | C | C | C | C | C | T | G | C | G | C | G | T | C | T | C | C | T | A | T | T | G | C | C | T | A | A | C | C | A | A | C | C | G | C | A | G | C | A | G | A | C | C | T | A | C | T | G | G | G | G | G | C |
| 89 | UPEC-121 | G | C | C | T | G | A | C | G | A | T | G | C | C | G | C | C | C | C | C | T | G | C | G | C | G | T | C | T | C | C | T | A | T | T | G | C | C | T | A | A | C | C | A | A | C | C | G | C | A | G | C | A | G | A | C | C | T | A | C | T | G | G | G | G | G | C |
| 90 | 81 | G | C | C | T | G | A | C | G | A | T | G | C | C | G | C | C | C | C | C | T | G | C | G | C | G | T | C | T | C | C | T | A | T | T | G | C | C | T | A | A | C | C | A | A | C | C | G | C | A | G | C | A | G | A | C | C | T | A | C | T | G | G | G | G | G | C |
| 91 | 99 | G | C | C | T | G | A | C | G | A | T | G | C | C | G | C | C | C | C | C | T | G | C | G | C | G | T | C | T | C | C | T | A | T | T | G | C | C | T | A | A | C | C | A | A | C | C | G | C | A | G | C | A | G | A | C | C | T | A | C | T | G | G | G | G | G | C |
| 92 | UPEC-104 | G | C | C | T | G | A | C | G | A | T | G | C | C | G | C | C | C | C | C | T | G | C | G | C | G | T | C | T | C | C | T | A | T | T | G | C | C | T | A | A | C | C | A | A | C | C | G | C | A | G | C | A | G | A | C | C | T | A | C | T | G | G | G | G | G | C |
| 93 | UPEC-111 | G | C | C | T | A | A | C | G | A | T | T | C | C | G | C | C | C | C | C | T | G | C | G | C | G | T | C | T | C | T | T | A | C | T | G | C | C | T | A | A | T | C | G | A | C | T | G | G | A | G | C | A | G | A | C | C | A | A | C | T | G | G | G | G | G | C |
| 94 | 93 | G | C | C | T | A | A | C | G | A | T | T | C | C | G | C | C | C | C | C | T | G | C | G | C | G | T | C | T | C | T | T | A | C | T | G | C | C | T | A | A | T | C | G | A | C | T | G | G | A | G | C | A | G | A | C | C | A | A | C | T | G | G | G | G | G | C |
| 95 | UPEC-238 | G | C | C | T | G | A | C | G | A | T | T | C | C | G | C | C | C | C | C | T | G | C | G | C | G | T | C | T | C | C | T | A | C | T | G | C | C | T | A | A | C | C | G | A | C | C | G | C | T | A | C | A | G | G | T | T | A | A | C | T | G | G | G | G | G | C |
| 96 | 121 | G | C | C | T | G | A | C | G | A | T | T | C | C | G | C | C | C | C | C | T | G | C | G | C | G | T | C | T | C | C | T | A | C | T | G | C | C | T | A | A | C | C | G | A | C | C | G | G | T | A | C | A | G | G | C | T | A | A | C | T | G | G | G | G | G | C |
| 97 | UPEC-211 | G | C | C | T | G | A | C | G | A | T | T | C | C | G | C | T | C | C | C | T | G | C | G | C | G | T | C | T | C | C | T | A | C | T | G | C | C | T | A | A | C | C | G | A | C | C | G | G | T | A | C | A | G | G | C | T | A | A | C | T | G | G | G | T | G | C |
| 98 | 67 | G | C | C | T | G | A | C | G | A | T | T | C | C | G | C | C | C | C | C | T | G | C | G | C | G | T | C | T | C | C | T | A | C | T | G | C | C | T | A | A | C | C | G | A | C | C | G | G | T | A | C | A | G | G | C | T | A | A | C | T | G | G | G | T | G | C |
| | 102 | G | C | C | T | G | A | C | G | A | T | T | C | C | G | C | C | C | C | C | T | G | C | G | C | G | T | C | T | C | C | T | A | C | T | G | C | C | T | A | A | C | C | G | A | C | C | G | G | T | A | C | A | G | G | C | T | A | A | C | T | G | G | G | T | G | C |
| | 113 | G | C | C | T | G | A | C | G | A | T | T | C | C | G | C | C | C | C | C | T | G | C | G | C | G | T | C | T | C | C | T | A | C | T | G | C | C | T | A | A | C | C | G | A | C | C | G | G | T | A | C | A | G | G | C | T | A | A | C | T | G | G | G | T | G | C |
| 99 | UMN026 | G | C | C | T | G | A | C | G | A | T | T | C | C | G | C | C | C | C | C | T | G | C | G | C | G | T | C | T | C | C | T | A | C | T | G | C | C | T | A | A | C | C | G | A | C | C | G | G | T | A | C | A | G | G | C | T | A | A | C | T | G | G | G | G | G | C |
| | 84 | G | C | C | T | G | A | C | G | A | T | T | C | C | G | C | C | C | C | C | T | G | C | G | C | G | T | C | T | C | C | T | A | C | T | G | C | C | T | A | A | C | C | G | A | C | C | G | G | T | A | C | A | G | G | C | T | A | A | C | T | G | G | G | G | G | C |
| | 86 | G | C | C | T | G | A | C | G | A | T | T | C | C | G | C | C | C | C | C | T | G | C | G | C | G | T | C | T | C | C | T | A | C | T | G | C | C | T | A | A | C | C | G | A | C | C | G | G | T | A | C | A | G | G | C | T | A | A | C | T | G | G | G | G | G | C |
| | 92 | G | C | C | T | G | A | C | G | A | T | T | C | C | G | C | C | C | C | C | T | G | C | G | C | G | T | C | T | C | C | T | A | C | T | G | C | C | T | A | A | C | C | G | A | C | C | G | G | T | A | C | A | G | G | C | T | A | A | C | T | G | G | G | G | G | C |
| | 103 | G | C | C | T | G | A | C | G | A | T | T | C | C | G | C | C | C | C | C | T | G | C | G | C | G | T | C | T | C | C | T | A | C | T | G | C | C | T | A | A | C | C | G | A | C | C | G | G | T | A | C | A | G | G | C | T | A | A | C | T | G | G | G | G | G | C |
| | 104 | G | C | C | T | G | A | C | G | A | T | T | C | C | G | C | C | C | C | C | T | G | C | G | C | G | T | C | T | C | C | T | A | C | T | G | C | C | T | A | A | C | C | G | A | C | C | G | G | T | A | C | A | G | G | C | T | A | A | C | T | G | G | G | G | G | C |
| | 111 | G | C | C | T | G | A | C | G | A | T | T | C | C | G | C | C | C | C | C | T | G | C | G | C | G | T | C | T | C | C | T | A | C | T | G | C | C | T | A | A | C | C | G | A | C | C | G | G | T | A | C | A | G | G | C | T | A | A | C | T | G | G | G | G | G | C |
| | 118 | G | C | C | T | G | A | C | G | A | T | T | C | C | G | C | C | C | C | C | T | G | C | G | C | G | T | C | T | C | C | T | A | C | T | G | C | C | T | A | A | C | C | G | A | C | C | G | G | T | A | C | A | G | G | C | T | A | A | C | T | G | G | G | G | G | C |

| | | 2153 | 2154 | 2156 | 2159 | 2171 | 2174 | 2192 | 2198 | 2204 | 2211 | 2219 | 2237 | 2240 | 2243 | 2249 | 2258 | 2270 | 2285 | 2300 | 2303 | 2306 | 2318 | 2321 | 2324 | 2339 | 2342 | 2345 | 2369 | 2372 | 2380 | 2387 | 2393 | 2398 | 2402 | 2408 | 2411 | 2413 | 2414 | 2420 | 2429 | 2435 | 2441 | 2444 | 2456 | 2459 | 2466 | 2468 | 2471 | 2474 | 2477 |
|---|---|---|---|---|---|---|---|---|---|---|---|---|---|---|---|---|---|---|---|---|---|---|---|---|---|---|---|---|---|---|---|---|---|---|---|---|---|---|---|---|---|---|---|---|---|---|---|---|---|---|---|
| 1 | 53 | G | T | G | T | C | C | C | C | C | C | C | C | C | C | C | A | C | G | T | C | C | G | G | G | G | G | G | A | A | C | C | G | G | A | C | C | C | A | C | C | C | T | C | C | C | T | A | G | G | G |
| 2 | Di2 | G | T | G | T | C | C | C | C | C | C | C | C | C | C | C | A | C | G | T | C | C | G | G | G | G | G | G | A | A | C | C | G | G | A | C | C | C | A | C | C | C | T | C | C | C | T | A | G | G | G |
| | Di14 | G | T | G | T | C | C | C | C | C | C | C | C | C | C | C | A | C | G | T | C | C | G | G | G | G | G | G | A | A | C | C | G | G | A | C | C | C | A | C | C | C | T | C | C | C | T | A | G | G | G |
| | CFT073 | G | T | G | T | C | C | C | C | C | C | C | C | C | C | C | A | C | G | T | C | C | G | G | G | G | G | G | A | A | C | C | G | G | A | C | C | C | A | C | C | C | T | C | C | C | T | A | G | G | G |
| 3 | 49 | G | T | G | T | C | C | C | C | C | C | C | C | C | C | C | A | C | G | T | C | C | G | G | G | G | G | G | A | A | C | C | G | G | A | C | C | C | A | C | C | C | T | C | C | C | T | A | G | G | G |
| | 87 | G | T | G | T | C | C | C | C | C | C | C | C | C | C | C | A | C | G | T | C | C | G | G | G | G | G | G | A | A | C | C | G | G | A | C | C | C | A | C | C | C | T | C | C | C | T | A | G | G | G |
| | 91 | G | T | G | T | C | C | C | C | C | C | C | C | C | C | C | A | C | G | T | C | C | G | G | G | G | G | G | A | A | C | C | G | G | A | C | C | C | A | C | C | C | T | C | C | C | T | A | G | G | G |
| | 95 | G | T | G | T | C | C | C | C | C | C | C | C | C | C | C | A | C | G | T | C | C | G | G | G | G | G | G | A | A | C | C | G | G | A | C | C | C | A | C | C | C | T | C | C | C | T | A | G | G | G |
| | 72 | G | T | G | T | C | C | C | C | C | C | C | C | C | C | C | A | C | G | T | C | C | G | G | G | G | G | G | A | A | C | C | G | G | A | C | C | C | A | C | C | C | T | C | C | C | T | A | G | G | G |
| 4 | UPEC-79 | G | T | G | T | C | C | C | C | C | C | C | C | C | C | C | A | C | G | T | C | C | G | G | G | G | G | G | A | A | C | C | G | G | A | C | C | C | A | C | C | C | T | C | C | C | T | A | G | G | G |
| 5 | 114 | G | T | G | T | C | C | C | C | C | C | C | C | C | C | C | A | C | G | T | C | C | G | G | G | G | G | G | A | A | C | C | G | G | A | C | C | C | A | C | C | C | T | C | C | C | T | A | G | G | G |
| 6 | ABU83972 | G | T | G | T | C | C | C | C | C | C | C | C | C | C | C | A | C | G | T | C | C | G | G | G | G | G | G | A | A | C | C | G | G | A | C | C | C | A | C | C | C | T | C | C | C | T | A | G | G | G |
| 7 | 94 | G | T | G | T | C | C | C | C | C | C | C | C | C | C | C | A | C | G | T | C | C | G | G | G | G | G | G | A | A | C | C | G | G | A | C | C | C | A | C | C | C | T | C | C | C | T | A | G | G | G |
| 8 | UPEC-116 | G | C | G | T | C | C | C | C | C | C | C | C | T | C | C | A | C | G | T | C | C | G | G | G | A | A | G | A | A | C | C | G | G | A | C | C | C | A | T | C | C | T | C | C | C | T | A | G | G | G |
| 9 | UPEC-180 | G | C | G | T | C | C | C | C | C | T | C | C | C | C | C | A | C | G | T | C | C | G | G | G | A | A | G | A | A | C | C | G | G | A | C | C | C | A | C | C | C | T | C | C | G | T | A | G | G | G |
| 10 | UPEC-100 | G | C | G | T | C | C | C | C | C | T | C | C | C | C | C | A | C | G | T | C | C | G | G | G | A | A | G | A | A | C | C | G | G | A | C | C | C | A | C | C | C | T | C | C | G | T | A | G | G | G |
| 11 | UPEC-112 | G | C | G | T | C | T | C | C | C | C | C | C | T | C | C | A | C | G | T | C | C | G | T | G | G | G | G | A | A | C | C | G | G | A | C | C | C | A | C | C | C | T | C | C | G | T | A | G | G | G |
| 12 | 85 | G | C | G | T | C | C | C | C | C | C | C | C | C | C | C | A | C | G | T | C | C | G | G | G | G | G | G | A | A | C | C | G | G | A | C | C | C | A | C | C | C | T | C | C | C | T | A | G | G | G |
| | UPEC-115 | G | C | G | T | C | C | C | C | C | C | C | C | C | C | C | A | C | G | T | C | C | G | G | G | G | G | G | A | A | C | C | G | G | A | C | C | C | A | C | C | C | T | C | C | C | T | A | G | G | G |
| 13 | UTI89 | G | C | G | T | C | C | C | C | C | C | C | C | C | C | C | A | C | G | T | C | C | G | G | G | G | G | G | A | A | C | C | G | G | A | C | C | C | A | C | C | C | T | C | C | C | T | A | G | G | G |
| 14 | UPEC-153 | G | C | G | T | C | C | C | C | C | C | C | C | C | C | C | A | C | G | T | C | C | G | G | G | G | G | G | A | A | C | C | G | G | A | C | C | C | A | C | C | C | T | C | C | C | T | A | G | G | G |
| 15 | 101 | G | C | G | T | C | C | C | C | T | C | C | C | C | C | C | A | C | G | T | C | C | G | G | G | A | A | G | A | A | C | C | G | G | A | C | C | C | A | C | C | C | T | C | C | G | T | A | G | G | G |
| 16 | 122 | G | C | G | T | C | C | C | C | T | C | C | C | C | C | C | A | C | G | T | C | C | G | G | G | A | A | G | A | A | C | C | G | G | A | C | C | C | A | C | C | C | T | C | C | G | T | A | G | G | G |
| 17 | 11 | G | C | G | T | C | C | C | C | T | C | C | C | C | C | C | A | C | G | T | C | C | G | G | G | A | A | G | A | A | C | C | G | G | A | C | C | C | A | C | C | C | T | C | C | G | T | A | G | G | G |
| 18 | 97 | G | C | G | T | C | C | C | C | T | C | C | C | C | C | C | A | C | G | T | C | C | G | G | G | A | A | G | A | A | C | C | G | G | A | C | C | C | A | C | C | C | T | C | C | G | T | A | G | G | G |
| 19 | UPEC-195 | G | C | G | T | C | C | C | C | T | C | C | C | C | C | C | A | C | G | T | C | C | G | G | G | A | A | G | A | A | C | C | G | G | A | C | C | C | A | C | C | C | T | C | C | G | T | A | G | G | G |
| 20 | UPEC-150 | G | C | G | T | C | C | C | C | C | C | C | C | T | C | C | A | C | G | T | C | C | G | G | G | A | A | G | A | A | C | C | G | G | A | C | C | C | A | T | C | C | T | C | C | C | T | A | G | G | G |
| 21 | UPEC-101 | G | C | G | T | C | C | C | C | C | C | C | C | C | C | C | A | C | G | T | C | C | G | G | G | G | G | G | A | A | C | C | G | G | A | C | C | C | A | C | C | C | T | C | C | C | T | A | G | G | G |
| 22 | UPEC-59 | G | C | G | T | C | C | C | C | C | C | C | C | C | C | C | A | C | G | T | C | C | G | G | G | G | G | G | A | A | C | C | G | G | A | C | C | C | A | C | C | C | T | C | C | C | T | A | G | G | G |
| 23 | 119 | G | C | G | T | C | C | C | C | C | T | C | C | C | C | C | A | C | G | T | C | C | G | G | G | A | A | G | A | A | C | C | G | G | A | C | C | C | A | C | C | C | T | C | C | G | T | A | A | G | G |
| 24 | 8 | G | C | G | T | C | C | C | C | C | T | C | C | C | C | C | A | C | G | T | C | C | G | G | G | A | A | G | A | A | C | C | G | G | A | C | C | C | A | C | C | C | T | C | C | G | T | A | A | G | G |
| 25 | 1 | G | C | G | T | C | C | C | C | C | T | C | C | C | C | C | A | C | G | T | C | C | G | G | G | A | A | G | A | A | C | C | G | G | A | C | C | C | A | C | C | C | T | C | C | G | T | A | A | G | G |
| 26 | 58 | G | C | G | T | C | C | C | C | C | T | C | C | C | C | C | A | C | G | T | C | C | G | G | G | A | A | G | A | A | C | C | G | G | A | C | C | C | A | C | C | C | T | C | C | G | T | A | A | G | G |
| | 71 | G | C | G | T | C | C | C | C | C | T | C | C | C | C | C | A | C | G | T | C | C | G | G | G | A | A | G | A | A | C | C | G | G | A | C | C | C | A | C | C | C | T | C | C | G | T | A | A | G | G |
| 27 | 68 | G | C | G | T | C | C | C | C | C | T | C | C | C | C | C | A | C | G | T | C | C | G | G | G | A | A | G | A | A | C | C | G | G | A | C | C | C | A | C | C | C | T | C | C | G | T | A | A | G | G |
| 28 | 107 | G | C | G | T | C | C | C | C | C | C | C | C | T | C | C | A | C | G | T | C | C | G | G | G | A | A | G | A | A | C | C | G | G | A | C | C | C | A | T | C | C | T | C | C | G | T | A | G | G | G |
| 29 | UPEC-179 | G | C | G | T | C | C | C | C | C | T | C | C | C | C | C | A | C | G | T | C | C | G | G | G | A | A | G | A | A | C | C | G | G | A | C | C | C | A | C | C | C | T | C | C | G | T | A | G | G | G |
| 30 | UPEC-78 | G | C | G | T | C | C | C | C | C | T | C | C | C | C | C | A | C | G | T | C | C | G | G | G | A | A | G | A | A | C | C | G | G | A | C | C | C | A | C | C | C | T | C | C | G | T | A | G | G | G |
| 31 | UPEC-274 | G | C | G | T | C | C | C | C | C | T | C | C | C | C | C | A | C | G | T | C | C | G | G | G | A | A | G | A | A | C | C | G | G | A | C | C | C | A | C | C | C | T | C | C | G | T | A | A | G | G |
| 32 | UPEC-243 | G | C | G | T | C | C | C | C | C | T | C | C | C | C | C | A | C | G | T | C | C | G | G | G | A | A | G | A | A | C | C | G | G | A | C | C | C | A | C | C | C | T | C | C | G | T | A | G | G | G |

| | | | | | | | | | | | | | | | | | | | | | | | | | | | | | | | | | | | | | | | | | | | | | | | | | | | |
|---|---|---|---|---|---|---|---|---|---|---|---|---|---|---|---|---|---|---|---|---|---|---|---|---|---|---|---|---|---|---|---|---|---|---|---|---|---|---|---|---|---|---|---|---|---|---|---|---|---|---|---|
| 33 | 5 | G | C | G | T | C | C | C | C | C | C | C | C | T | C | C | A | C | G | T | C | C | G | G | G | A | A | G | A | A | C | C | G | G | A | C | C | C | A | T | C | C | T | C | C | C | T | A | G | G | G |
| 34 | 70 | G | C | G | T | C | C | C | C | C | C | C | C | C | C | C | A | C | G | T | C | C | G | G | G | A | A | G | A | A | C | C | G | G | A | C | C | C | A | C | C | C | T | C | C | G | T | A | G | G | G |
| | 115 | G | C | G | T | C | C | C | C | C | C | C | C | C | C | C | A | C | G | T | C | C | G | G | G | A | A | G | A | A | C | C | G | G | A | C | C | C | A | C | C | C | T | C | C | G | T | A | G | G | G |
| 35 | 536 | G | C | G | T | C | C | C | C | C | C | C | C | C | C | C | A | C | G | T | C | C | G | G | G | A | A | G | A | A | C | C | G | G | A | C | C | C | A | C | C | C | T | C | C | G | T | A | G | G | G |
| 36 | F11 | G | C | G | T | C | C | C | C | C | C | C | C | C | C | C | A | C | G | T | C | C | G | G | G | A | A | G | A | A | C | C | G | G | A | C | C | C | A | C | C | C | T | C | C | G | T | A | G | G | G |
| 37 | UPEC-38 | G | C | G | T | C | C | C | T | C | C | C | C | T | C | C | A | C | G | T | C | C | G | G | G | A | A | G | A | A | C | C | G | G | A | C | C | C | A | T | C | C | T | C | C | C | T | A | G | G | G |
| 38 | UPEC-58 | G | C | G | T | C | C | C | C | C | C | C | C | C | C | C | A | C | G | T | C | C | G | G | G | G | G | G | A | A | C | C | G | G | A | C | C | C | A | C | C | C | T | C | C | C | T | A | G | G | G |
| 39 | UPEC-219 | G | C | G | T | C | C | C | T | C | C | C | C | T | C | C | A | C | G | T | C | C | G | G | G | A | A | G | A | A | C | C | G | G | A | C | C | C | A | T | C | C | T | C | C | C | T | A | G | G | G |
| 40 | 69 | G | C | G | T | C | C | C | T | C | C | C | C | T | C | C | A | C | G | T | C | C | G | G | G | A | A | G | A | A | C | C | G | G | A | C | C | C | A | T | C | C | T | C | C | C | T | A | G | G | G |
| | 110 | G | C | G | T | C | C | C | T | C | C | C | C | T | C | C | A | C | G | T | C | C | G | G | G | A | A | G | A | A | C | C | G | G | A | C | C | C | A | T | C | C | T | C | C | C | T | A | G | G | G |
| 41 | UPEC-266 | G | C | G | T | C | C | C | C | C | C | C | C | T | C | C | A | C | G | T | C | C | G | G | G | A | A | G | A | A | C | C | G | G | A | C | C | C | A | T | C | C | T | C | C | G | T | A | G | G | G |
| 42 | UPEC-202 | G | C | G | T | C | C | C | C | C | C | C | C | C | C | T | A | C | G | T | T | C | A | G | G | G | G | G | G | G | C | A | A | A | G | C | T | C | A | C | C | T | T | T | T | C | T | A | G | G | G |
| 43 | UPEC-233 | G | C | G | T | C | C | C | C | C | C | C | C | C | C | C | A | C | G | T | C | C | G | G | G | G | G | G | A | A | C | C | G | G | A | C | C | C | A | C | C | C | T | C | C | C | T | A | G | G | G |
| 44 | 109 | G | C | G | T | C | C | C | C | C | C | C | C | C | C | C | A | C | G | T | C | C | G | G | G | G | G | G | A | A | C | C | G | G | A | C | C | C | A | C | C | C | T | C | C | C | T | A | G | G | G |
| 45 | UPEC-236 | G | C | G | T | C | C | C | C | C | C | C | C | C | C | C | A | C | G | T | C | C | G | G | G | G | G | G | A | A | C | C | G | G | A | C | C | C | A | C | C | C | T | C | C | C | T | A | G | G | G |
| 46 | NA114 | G | C | G | T | C | C | C | T | C | C | C | C | T | C | C | A | C | G | T | C | C | G | G | G | A | A | G | A | A | C | C | G | G | A | C | C | C | A | T | C | C | T | C | C | C | T | A | G | G | G |
| | 108 | G | C | G | T | C | C | C | T | C | C | C | C | T | C | C | A | C | G | T | C | C | G | G | G | A | A | G | A | A | C | C | G | G | A | C | C | C | A | T | C | C | T | C | C | C | T | A | G | G | G |
| | Ec958 | G | C | G | T | C | C | C | T | C | C | C | C | T | C | C | A | C | G | T | C | C | G | G | G | A | A | G | A | A | C | C | G | G | A | C | C | C | A | T | C | C | T | C | C | C | T | A | G | G | G |
| 47 | 66 | G | C | G | T | C | C | C | T | C | C | C | C | T | C | C | A | C | G | T | C | C | G | G | G | A | A | G | A | A | C | C | G | G | A | C | C | C | A | T | C | C | T | C | C | C | T | A | G | G | G |
| | 105 | G | C | G | T | C | C | C | T | C | C | C | C | T | C | C | A | C | G | T | C | C | G | G | G | A | A | G | A | A | C | C | G | G | A | C | C | C | A | T | C | C | T | C | C | C | T | A | G | G | G |
| 48 | 112 | G | C | G | T | C | C | C | C | C | C | C | C | C | C | C | A | C | G | T | C | C | G | G | G | G | G | G | A | A | C | C | G | G | A | C | C | C | A | C | C | C | T | C | C | C | T | A | G | G | G |
| 49 | 98 | G | C | G | T | C | C | C | T | C | C | C | C | T | C | C | A | C | G | T | C | C | G | G | G | A | A | G | A | A | C | C | G | G | A | C | C | C | A | T | C | C | T | C | C | C | T | A | G | G | G |
| 50 | 74 | G | C | G | T | C | C | C | C | C | C | C | C | C | C | C | A | C | G | T | C | C | G | G | G | G | G | G | A | A | C | C | G | G | A | C | C | C | A | C | C | C | T | C | C | C | T | A | G | G | G |
| 51 | 60 | G | C | G | T | C | C | C | C | C | C | C | C | C | C | C | A | C | G | T | C | C | G | G | G | G | G | G | A | A | C | C | G | G | A | C | C | C | A | C | C | C | T | C | C | C | T | A | G | G | G |
| 52 | 47 | G | C | G | T | C | C | C | C | C | C | C | C | C | C | C | A | C | G | T | C | C | G | G | G | G | G | G | A | A | C | C | G | G | A | C | C | C | A | C | C | C | T | C | C | C | T | A | G | G | G |
| 53 | UPEC-149 | G | C | G | T | C | C | C | C | C | C | C | C | C | C | C | A | C | G | T | C | C | G | G | G | G | A | G | A | A | C | C | G | G | A | C | C | C | A | C | C | C | T | C | C | G | T | A | G | G | G |
| 54 | 57 | G | C | G | T | C | C | C | C | C | C | C | C | C | C | C | A | C | G | T | C | C | G | G | G | A | A | G | A | A | C | C | G | G | A | C | C | C | A | C | C | C | T | C | C | G | T | A | G | G | G |
| | 59 | G | C | G | T | C | C | C | C | C | C | C | C | C | C | C | A | C | G | T | C | C | G | G | G | A | A | G | A | A | C | C | G | G | A | C | C | C | A | C | C | C | T | C | C | G | T | A | G | G | G |
| | 77 | G | C | G | T | C | C | C | C | C | C | C | C | C | C | C | A | C | G | T | C | C | G | G | G | A | A | G | A | A | C | C | G | G | A | C | C | C | A | C | C | C | T | C | C | G | T | A | G | G | G |
| | 90 | G | C | G | T | C | C | C | C | C | C | C | C | C | C | C | A | C | G | T | C | C | G | G | G | A | A | G | A | A | C | C | G | G | A | C | C | C | A | C | C | C | T | C | C | G | T | A | G | G | G |
| | UPEC-258 | G | C | G | T | C | C | C | C | C | C | C | C | C | C | C | A | C | G | T | C | C | G | G | G | A | A | G | A | A | C | C | G | G | A | C | C | C | A | C | C | C | T | C | C | G | T | A | G | G | G |
| 55 | 75 | G | C | G | T | C | C | C | C | C | C | C | C | C | C | C | A | C | G | T | C | C | G | G | G | A | A | G | A | A | C | C | G | G | A | C | C | T | A | T | C | C | T | C | C | C | T | A | G | G | G |
| 56 | 62 | G | G | G | T | C | C | T | C | C | C | T | T | C | C | C | G | C | G | T | C | T | G | G | G | G | A | G | G | G | C | A | A | A | G | C | T | C | A | C | C | C | A | C | T | C | C | A | A | G | G |
| | UPEC-137 | G | G | G | T | C | C | T | C | C | C | T | T | C | C | C | G | C | G | T | C | T | G | G | G | G | A | G | G | G | C | A | A | A | G | C | T | C | A | C | C | C | A | C | T | C | C | A | A | G | G |
| 57 | UPEC-213 | G | G | G | T | C | C | T | C | C | C | C | T | C | C | C | G | T | G | T | C | T | G | G | G | G | G | G | G | G | C | A | G | G | A | G | C | C | A | C | C | C | A | C | T | C | C | A | A | G | G |
| 58 | 4 | G | G | G | T | C | C | C | C | C | C | C | C | C | C | C | A | C | G | T | C | C | G | G | G | G | G | G | G | G | C | A | A | G | A | G | T | C | A | C | C | C | A | C | C | C | C | A | A | A | G |
| 59 | 3 | A | T | G | T | C | C | C | C | C | C | C | C | C | C | T | G | C | G | T | T | C | A | G | G | G | G | G | G | G | C | A | G | G | A | C | T | C | A | C | A | C | A | C | C | C | C | T | A | G | G |
| 60 | UPEC-103 | G | G | G | T | C | C | C | C | C | C | C | C | C | C | C | A | C | G | T | C | C | G | G | G | G | G | G | G | G | C | A | G | A | G | C | T | C | A | C | A | C | A | C | C | C | C | A | A | G | G |
| 61 | UPEC-200 | G | G | G | T | C | C | C | C | C | C | C | C | C | C | C | A | C | G | T | C | C | G | G | G | G | G | G | T | G | C | A | G | G | G | C | T | C | A | C | C | T | T | T | T | C | T | A | A | G | G |
| 62 | 54 | G | C | G | T | C | C | C | C | C | C | C | C | C | T | C | A | C | G | T | C | C | G | G | G | G | G | G | T | G | C | A | A | A | G | C | T | C | T | C | C | T | T | T | T | C | T | A | G | G | G |
| | 80 | G | C | G | T | C | C | C | C | C | C | C | C | C | T | C | A | C | G | T | C | C | G | G | G | G | G | G | T | G | C | A | A | A | G | C | T | C | T | C | C | T | T | T | T | C | T | A | G | G | G |
| 63 | 100 | G | C | G | T | C | C | C | C | C | C | C | C | C | T | C | A | C | G | T | C | C | G | G | G | G | G | G | T | G | T | A | A | A | G | C | T | C | T | C | C | T | T | T | T | C | T | A | G | G | G |
| 64 | 14 | G | C | G | T | C | C | C | C | C | C | C | C | C | T | C | A | C | G | T | C | C | G | G | T | G | G | G | T | G | C | A | A | A | G | C | T | C | T | C | C | T | T | T | T | C | T | A | G | G | G |
| 65 | 123 | G | C | G | T | C | C | C | C | C | C | C | C | C | C | C | A | C | G | T | C | C | G | G | G | G | G | G | G | G | C | A | G | G | A | G | T | C | A | C | C | C | A | C | C | C | C | A | A | G | G |
| 66 | 83 | G | C | G | T | C | C | C | C | C | C | C | C | C | C | C | A | C | G | T | C | C | G | G | G | G | G | G | G | G | C | A | G | G | A | G | T | C | A | C | C | C | A | C | C | C | C | A | A | A | G |

| | | | | | | | | | | | | | | | | | | | | | | | | | | | | | | | | | | | | | | | | | | | | | | | | | | | |
|---|---|---|---|---|---|---|---|---|---|---|---|---|---|---|---|---|---|---|---|---|---|---|---|---|---|---|---|---|---|---|---|---|---|---|---|---|---|---|---|---|---|---|---|---|---|---|---|---|---|---|---|
| 67 | 64 | G | C | G | T | C | C | C | C | C | C | C | C | C | C | C | A | C | G | T | C | C | G | G | G | G | G | G | G | G | C | A | A | G | A | G | T | C | A | C | C | C | A | C | C | C | C | A | A | A | G |
| | UPEC-205 | G | C | G | T | C | C | C | C | C | C | C | C | C | C | C | A | C | G | T | C | C | G | G | G | G | G | G | G | G | C | A | A | G | A | G | T | C | A | C | C | C | A | C | C | C | C | A | A | A | G |
| 68 | UPEC-132 | G | C | T | T | C | C | C | C | C | C | C | C | C | C | T | G | C | G | T | T | C | A | G | G | G | G | G | G | G | C | A | G | G | A | C | T | C | A | C | A | C | A | C | C | C | C | A | A | G | A |
| 69 | 120 | G | C | T | T | C | C | C | C | C | C | C | C | C | C | T | G | C | G | T | T | C | A | G | G | G | G | G | G | G | C | A | G | G | A | C | T | C | A | C | A | C | A | C | C | C | C | A | A | G | A |
| 70 | UPEC-105 | G | C | G | A | C | C | C | C | C | C | C | C | C | C | C | A | C | G | T | C | C | G | G | G | G | G | A | G | G | C | A | G | A | G | G | C | C | A | C | C | T | T | C | T | C | C | A | A | G | G |
| 71 | UPEC-175 | G | C | T | T | C | C | C | C | C | C | C | C | C | C | T | G | C | G | T | T | C | A | G | G | G | G | G | G | G | C | A | G | G | A | C | T | C | A | C | A | C | A | C | C | C | C | A | A | G | G |
| 72 | UPEC-133 | A | T | G | T | C | C | C | C | C | C | C | C | C | C | T | G | C | G | T | T | C | A | G | G | G | G | G | G | G | C | A | G | G | A | C | T | C | A | C | A | C | A | C | C | C | C | A | A | G | G |
| 73 | 96 | A | T | G | T | T | C | C | C | C | C | C | C | C | C | T | G | C | G | T | T | C | A | G | G | G | G | G | G | G | C | A | G | G | A | G | T | C | A | C | A | C | A | C | C | C | C | A | A | G | G |
| 74 | 89 | G | G | G | T | C | C | C | C | C | C | C | C | C | T | C | A | C | G | T | C | C | G | G | G | G | G | G | T | G | C | A | A | A | G | C | T | C | T | C | C | T | T | T | T | C | T | A | G | G | G |
| | UPEC-127 | G | G | G | T | C | C | C | C | C | C | C | C | C | T | C | A | C | G | T | C | C | G | G | G | G | G | G | T | G | C | A | A | A | G | C | T | C | T | C | C | T | T | T | T | C | T | A | G | G | G |
| 75 | 55 | G | G | G | T | C | C | C | C | C | C | C | C | C | T | C | A | C | G | T | C | C | G | G | G | G | G | G | T | G | C | A | A | A | G | C | T | C | T | C | C | T | T | T | T | C | T | A | G | G | G |
| 76 | IAI39 | G | G | G | T | C | C | C | C | C | C | C | C | C | C | T | G | C | G | T | C | T | G | G | G | G | A | G | G | G | C | A | A | A | G | C | T | C | A | C | C | C | A | C | T | C | C | A | A | G | G |
| 77 | 124 | G | G | G | T | C | C | C | C | C | C | C | C | C | C | T | G | C | G | T | C | T | G | G | G | G | A | G | G | G | C | A | A | A | G | C | T | C | A | C | C | C | A | C | T | C | C | A | A | G | G |
| 78 | 76 | G | C | G | T | C | C | C | C | C | C | C | C | C | C | C | A | C | G | T | C | C | G | G | G | G | G | G | G | G | C | A | A | G | A | G | T | C | A | C | C | C | A | C | C | C | C | A | A | A | G |
| | UPEC-146 | G | C | G | T | C | C | C | C | C | C | C | C | C | C | C | A | C | G | T | C | C | G | G | G | G | G | G | G | G | C | A | A | G | A | G | T | C | A | C | C | C | A | C | C | C | C | A | A | A | G |
| 79 | 79 | G | C | G | T | C | C | C | C | C | C | C | C | C | C | C | A | C | C | T | C | C | G | G | G | G | G | G | G | G | C | A | G | G | A | G | T | C | A | C | C | C | A | C | C | C | C | A | A | A | G |
| 80 | UPEC-130 | G | C | G | T | C | C | C | C | C | C | C | C | C | C | C | A | C | G | T | C | C | G | G | G | G | G | G | G | G | C | A | G | G | A | C | T | C | A | C | C | C | A | C | C | C | C | A | A | A | G |
| 81 | 7 | G | C | G | T | C | C | C | C | C | C | C | C | C | C | C | A | C | C | T | C | C | G | G | G | G | G | G | G | G | C | A | G | G | A | G | T | C | A | C | A | C | A | C | C | C | C | A | A | G | G |
| 82 | UPEC-33 | G | C | G | T | C | C | C | C | C | C | C | C | C | C | C | A | C | G | T | C | C | G | G | G | G | G | G | G | G | C | A | A | G | A | G | T | C | A | C | C | C | A | C | C | C | C | A | A | A | G |
| 83 | UPEC-31 | G | C | G | T | C | C | C | C | C | C | C | C | C | C | C | A | C | G | T | C | C | G | G | G | G | G | G | G | G | C | A | A | G | A | G | T | C | A | C | C | C | A | C | T | C | C | A | A | G | G |
| 84 | UPEC-119 | G | C | G | T | C | C | C | C | C | C | C | C | C | T | C | A | C | G | C | C | C | G | G | G | G | G | G | G | G | C | A | A | A | G | C | T | C | A | C | C | T | T | T | T | C | C | A | A | G | G |
| 85 | 65 | G | C | G | T | C | C | C | C | C | C | C | C | C | C | C | A | C | G | T | C | C | G | G | G | G | G | G | G | G | C | A | G | G | A | G | T | C | A | C | C | C | A | C | C | C | C | A | A | A | G |
| 86 | 88 | G | C | G | T | C | C | C | C | C | C | C | C | C | C | C | A | C | G | T | C | C | G | G | G | G | G | G | G | G | C | A | G | G | A | C | T | C | A | C | C | C | A | C | C | C | C | A | A | A | G |
| 87 | 78 | G | C | G | T | C | C | C | C | C | C | C | C | C | C | C | A | C | G | T | C | C | G | G | G | G | G | G | G | G | C | A | A | G | A | G | T | C | A | C | C | C | A | C | C | C | C | A | A | A | G |
| 88 | 82 | G | C | G | T | C | C | C | C | C | C | C | C | C | C | C | A | C | G | T | C | C | G | G | G | G | G | G | G | G | C | A | A | G | A | G | T | C | A | C | C | C | A | C | C | C | C | A | A | A | G |
| 89 | UPEC-121 | G | C | G | T | C | C | C | C | C | C | C | C | C | C | C | A | C | C | T | C | C | G | G | G | G | G | G | G | G | C | A | G | G | A | G | T | C | A | C | C | C | A | C | C | C | C | A | A | A | G |
| 90 | 81 | G | C | G | T | C | C | C | C | C | C | C | C | C | C | C | A | C | G | T | C | C | G | G | G | G | G | G | G | G | C | A | A | G | A | G | T | C | A | C | C | C | A | C | C | C | C | A | A | A | G |
| 91 | 99 | G | C | G | T | C | C | C | C | C | C | C | C | C | C | C | A | C | G | T | C | C | G | G | G | G | G | G | G | G | C | A | A | G | A | G | T | C | A | C | C | C | A | C | T | C | C | A | A | G | G |
| 92 | UPEC-104 | G | C | G | T | C | C | C | C | C | C | C | C | C | C | C | A | C | G | T | C | C | G | G | G | G | G | G | G | G | C | A | G | G | A | G | T | C | A | C | A | C | A | C | C | C | C | A | A | G | G |
| 93 | UPEC-111 | G | C | G | T | C | C | C | C | C | C | C | C | C | C | C | A | C | G | T | C | C | G | G | G | G | G | G | G | G | C | A | A | G | A | G | T | C | A | C | C | C | A | C | T | C | C | A | A | G | G |
| 94 | 93 | G | C | G | T | C | C | C | C | C | C | C | C | C | C | C | A | C | G | T | C | C | G | G | G | G | G | G | G | G | C | A | A | G | A | G | T | C | A | C | C | C | A | C | C | C | C | A | A | A | G |
| 95 | UPEC-238 | G | C | G | T | C | C | C | C | C | C | C | C | C | C | C | A | C | G | T | C | C | G | G | G | G | G | G | G | G | C | A | G | G | A | G | T | C | A | C | A | C | A | C | C | C | C | A | A | G | G |
| 96 | 121 | G | G | G | T | C | C | C | C | C | C | C | C | C | C | C | A | C | G | T | C | C | G | G | G | G | G | G | G | G | C | A | A | G | A | G | T | C | A | C | C | C | A | C | C | C | C | A | A | A | G |
| 97 | UPEC-211 | G | G | G | T | C | C | T | C | C | C | C | C | C | C | T | G | C | G | T | T | C | A | G | G | G | G | G | G | G | C | A | A | A | G | G | C | C | A | C | C | C | A | C | T | C | C | A | A | G | G |
| 98 | 67 | G | G | G | T | C | C | T | C | C | C | C | C | C | C | T | G | C | G | T | T | C | A | G | G | G | G | G | G | G | C | A | A | A | G | G | C | C | A | C | C | C | A | C | T | C | C | A | A | G | G |
| | 102 | G | G | G | T | C | C | T | C | C | C | C | C | C | C | T | G | C | G | T | T | C | A | G | G | G | G | G | G | G | C | A | A | A | G | G | C | C | A | C | C | C | A | C | T | C | C | A | A | G | G |
| | 113 | G | G | G | T | C | C | T | C | C | C | C | C | C | C | T | G | C | G | T | T | C | A | G | G | G | G | G | G | G | C | A | A | A | G | G | C | C | A | C | C | C | A | C | T | C | C | A | A | G | G |
| 99 | UMN026 | A | T | G | T | T | C | C | C | C | C | C | C | C | C | T | G | C | G | T | T | C | A | G | G | G | G | G | G | G | C | A | G | G | A | G | T | C | A | C | A | C | A | C | C | C | C | A | A | G | G |
| | 84 | A | T | G | T | T | C | C | C | C | C | C | C | C | C | T | G | C | G | T | T | C | A | G | G | G | G | G | G | G | C | A | G | G | A | G | T | C | A | C | A | C | A | C | C | C | C | A | A | G | G |
| | 86 | A | T | G | T | T | C | C | C | C | C | C | C | C | C | T | G | C | G | T | T | C | A | G | G | G | G | G | G | G | C | A | G | G | A | G | T | C | A | C | A | C | A | C | C | C | C | A | A | G | G |
| | 92 | A | T | G | T | T | C | C | C | C | C | C | C | C | C | T | G | C | G | T | T | C | A | G | G | G | G | G | G | G | C | A | G | G | A | G | T | C | A | C | A | C | A | C | C | C | C | A | A | G | G |
| | 103 | A | T | G | T | T | C | C | C | C | C | C | C | C | C | T | G | C | G | T | T | C | A | G | G | G | G | G | G | G | C | A | G | G | A | G | T | C | A | C | A | C | A | C | C | C | C | A | A | G | G |
| | 104 | A | T | G | T | T | C | C | C | C | C | C | C | C | C | T | G | C | G | T | T | C | A | G | G | G | G | G | G | G | C | A | G | G | A | G | T | C | A | C | A | C | A | C | C | C | C | A | A | G | G |
| | 111 | A | T | G | T | T | C | C | C | C | C | C | C | C | C | T | G | C | G | T | T | C | A | G | G | G | G | G | G | G | C | A | G | G | A | G | T | C | A | C | A | C | A | C | C | C | C | A | A | G | G |
| | 118 | A | T | G | T | T | C | C | C | C | C | C | C | C | C | T | G | C | G | T | T | C | A | G | G | G | G | G | G | G | C | A | G | G | A | G | T | C | A | C | A | C | A | C | C | C | C | A | A | G | G |
