# Supplementary material for: A High-resolution Typing Assay for Uropathogenic Escherichia coli Based on Fimbrial Diversity
Source: Front Microbiol. 2016 Apr 29;7:623. doi: 10.3389/fmicb.2016.00623 (PMC4850163; doi:10.3389/fmicb.2016.00623)
Supplement: Supplementary file 6 [file Image_1.PDF]

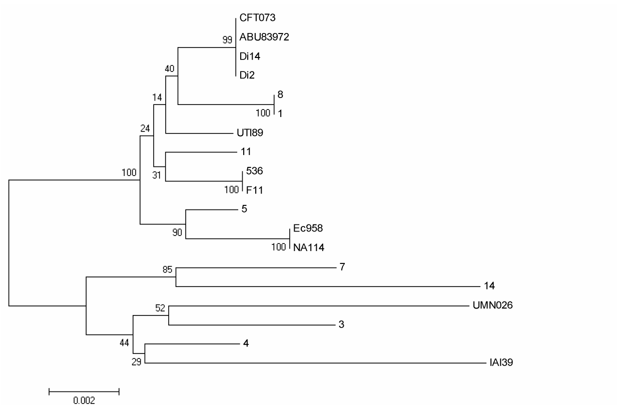

(a)

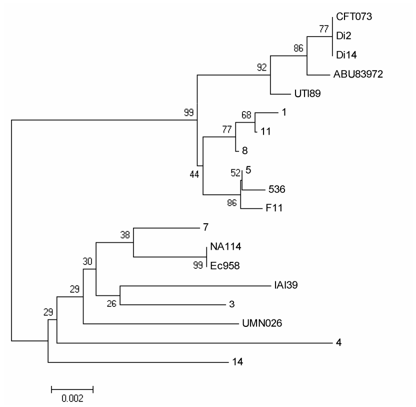

(b)

Fig. S1. Phylogenetic trees of 19 UPEC strains based on (a) MLST genes and (b) full-length *fimH* gene. The scales at the bottoms of the trees indicate phylogenetic distance. Bootstrap values are displayed as percentages on nodes.
