# Supplementary material for: A High-resolution Typing Assay for Uropathogenic Escherichia coli Based on Fimbrial Diversity
Source: Front Microbiol. 2016 Apr 29;7:623. doi: 10.3389/fmicb.2016.00623 (PMC4850163; doi:10.3389/fmicb.2016.00623)
Supplement: Supplementary file 7 [file Image_2.PDF]

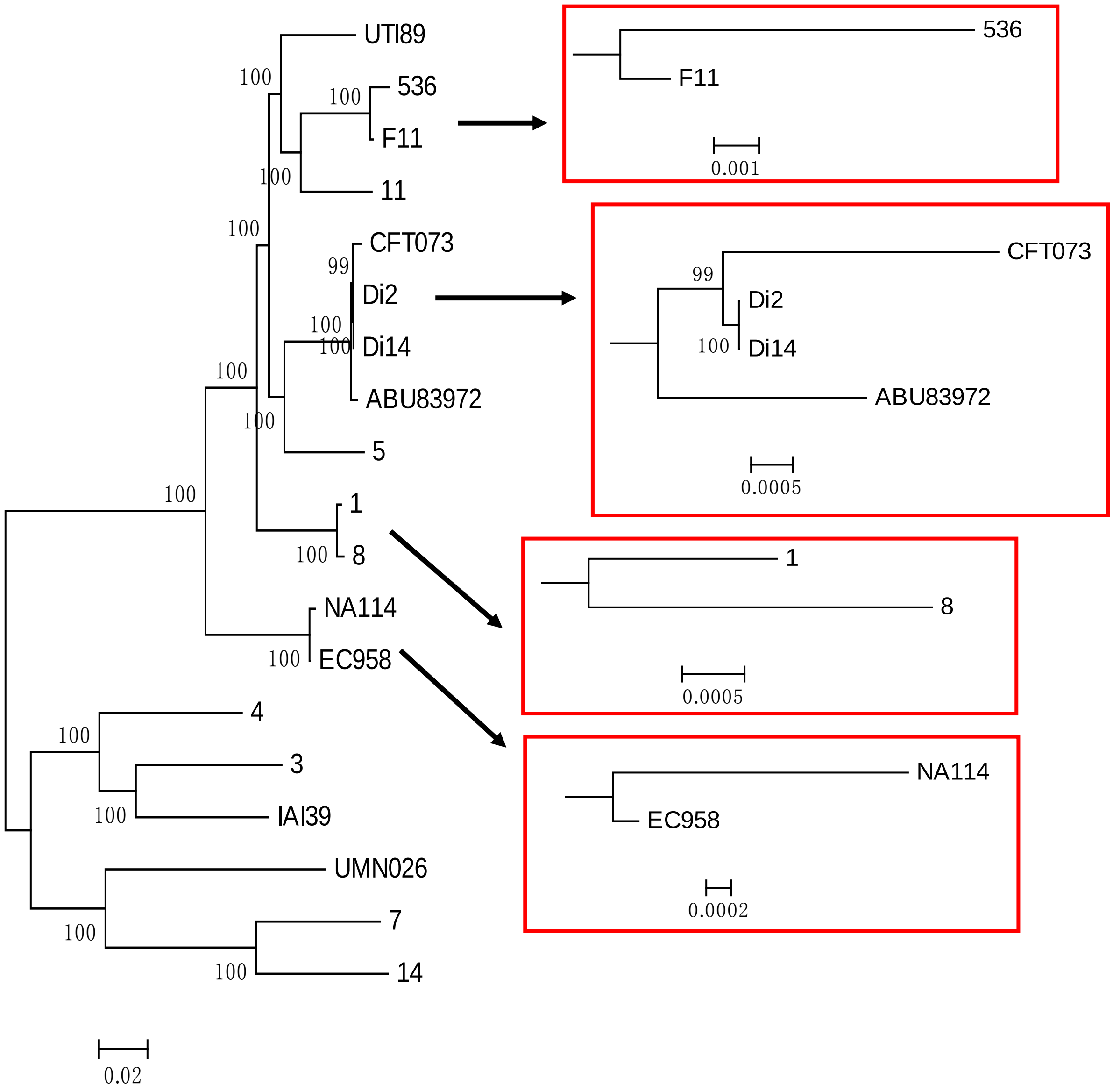

Fig. S2. Phylogenetic tree of UPEC strains based on the homologous genes from 19 strains. The scale at the bottom of the tree indicates phylogenetic distance. Bootstrap values are displayed as percentages on nodes. Closely related strains are shown separately in red boxes.
