# Supplementary material for: A High-resolution Typing Assay for Uropathogenic Escherichia coli Based on Fimbrial Diversity
Source: Front Microbiol. 2016 Apr 29;7:623. doi: 10.3389/fmicb.2016.00623 (PMC4850163; doi:10.3389/fmicb.2016.00623)
Supplement: Supplementary file 8 [file Image_3.PDF]

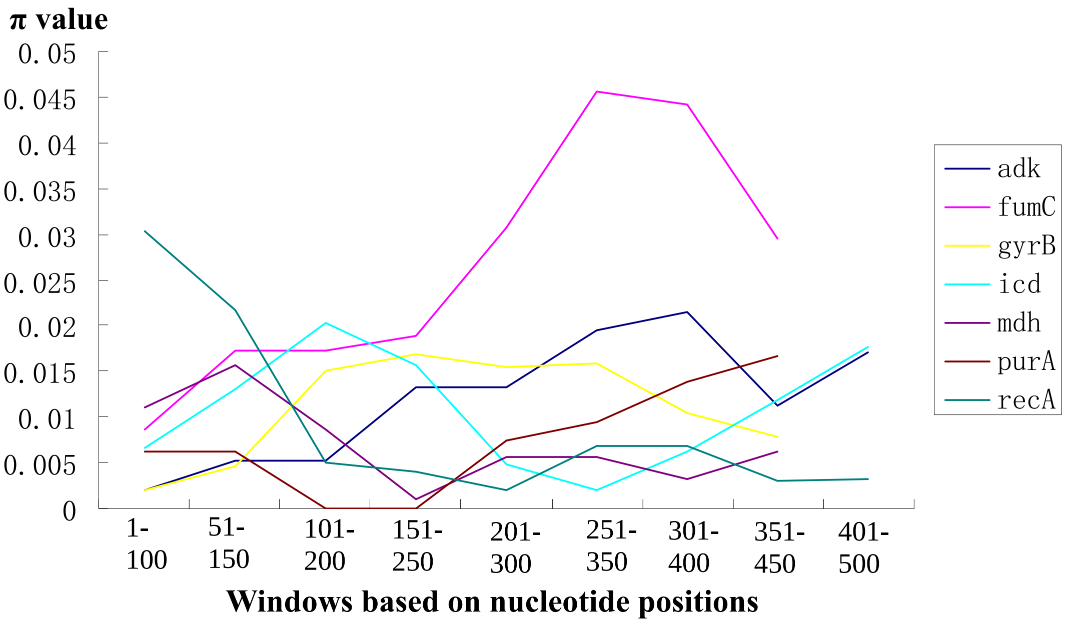
π value
0. 05
0. 045
0. 04
0. 035
0. 03
0. 025
0. 02
0. 015
0. 01
0. 005
0
1-100
51-150
101-200
151-250
201-300
251-350
301-400
351-450
401-500
Windows based on nucleotide positions
adk
fumC
gyrB
icd
mdh
purA
recA
(a)

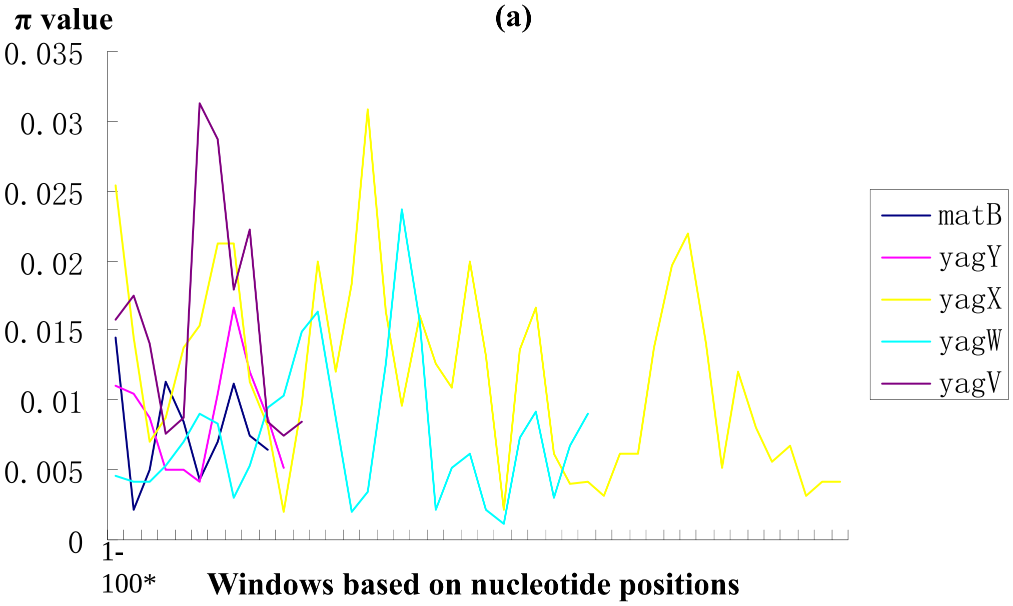
π value
0. 035
0. 03
0. 025
0. 02
0. 015
0. 01
0. 005
0
1-100*
Windows based on nucleotide positions
matB
yagY
yagX
yagW
yagV
(b)

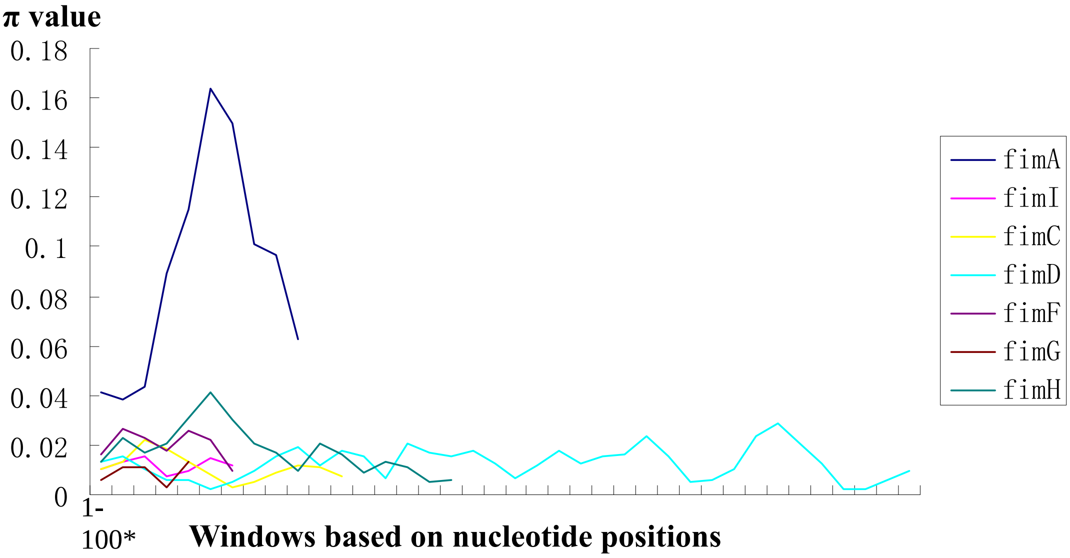
π value
0. 18
0. 16
0. 14
0. 12
0. 1
0. 08
0. 06
0. 04
0. 02
0
1-100*
Windows based on nucleotide positions
fimA
fimI
fimC
fimD
fimF
fimG
fimH
(c)

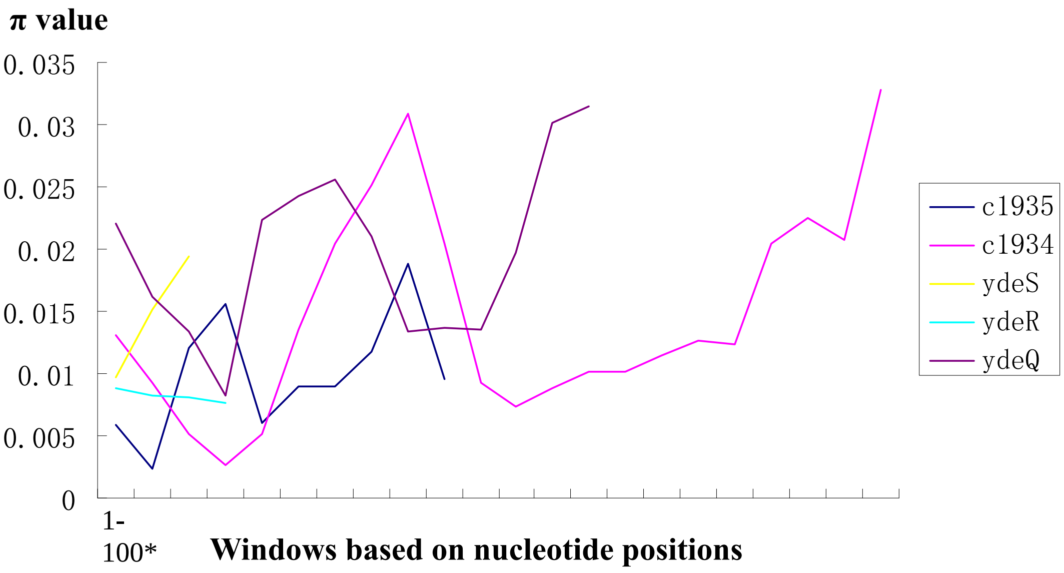
π value
0. 035
0. 03
0. 025
0. 02
0. 015
0. 01
0. 005
0
1-
100*
Windows based on nucleotide positions
c1935
c1934
ydeS
ydeR
ydeQ

(d)

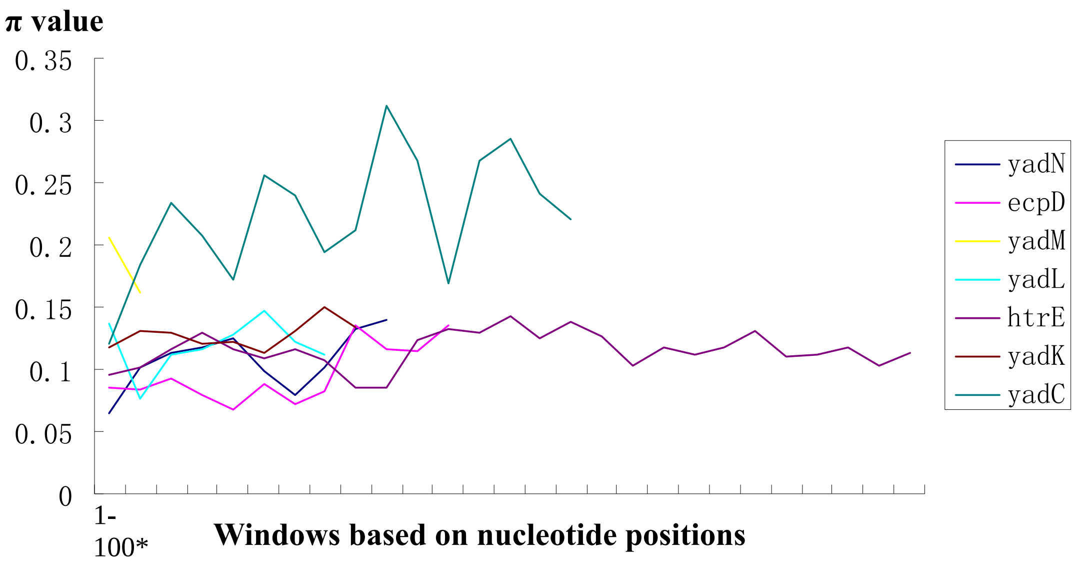
π value
0. 35
0. 3
0. 25
0. 2
0. 15
0. 1
0. 05
0
1-
100*
Windows based on nucleotide positions
yadN
ecpD
yadM
yadL
htrE
yadK
yadC

(e)

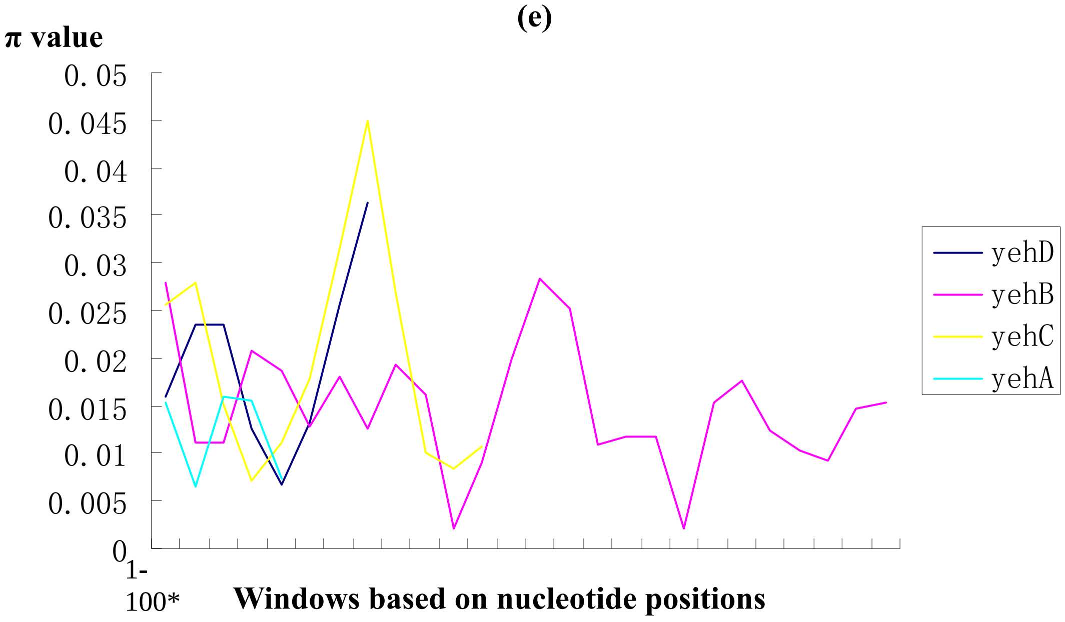
π value
0. 05
0. 045
0. 04
0. 035
0. 03
0. 025
0. 02
0. 015
0. 01
0. 005
0
1-
100*
Windows based on nucleotide positions
yehD
yehB
yehC
yehA

(f)

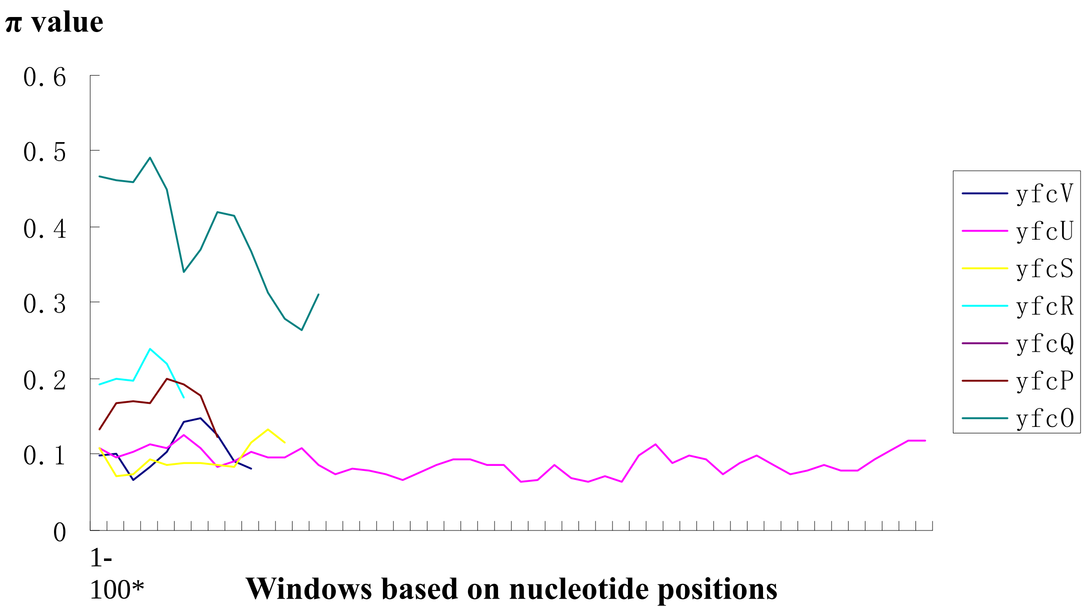


**(g)**

**Fig. S3.** Sliding-window Nucleotide polymorphism plot of MLST genes and genes in six common chaperone-usher fimbriae of UPEC strains. (a) 7 MLST genes, (b) genes in Mat fimbriae, (c) genes in Type 1 fimbriae, (d) genes in F9 fimbriae, (e) genes in Yad fimbriae, (f) genes in Yeh fimbriae, (g) genes in Yfc fimbriae. Overlapping windows of 100 nt with a step size of 50 nt are used. The scale on the X axis indicates overlapping nucleotide positions such as 1-100, 51-150, 101-200, 151-250, 201-300, 251-305 and so on, some positions are not displayed in the figure for the limited space *. Gene names are indicated on the right.
