# Supplementary material for: A High-resolution Typing Assay for Uropathogenic Escherichia coli Based on Fimbrial Diversity
Source: Front Microbiol. 2016 Apr 29;7:623. doi: 10.3389/fmicb.2016.00623 (PMC4850163; doi:10.3389/fmicb.2016.00623)
Supplement: Supplementary file 9 [file Image_4.PDF]

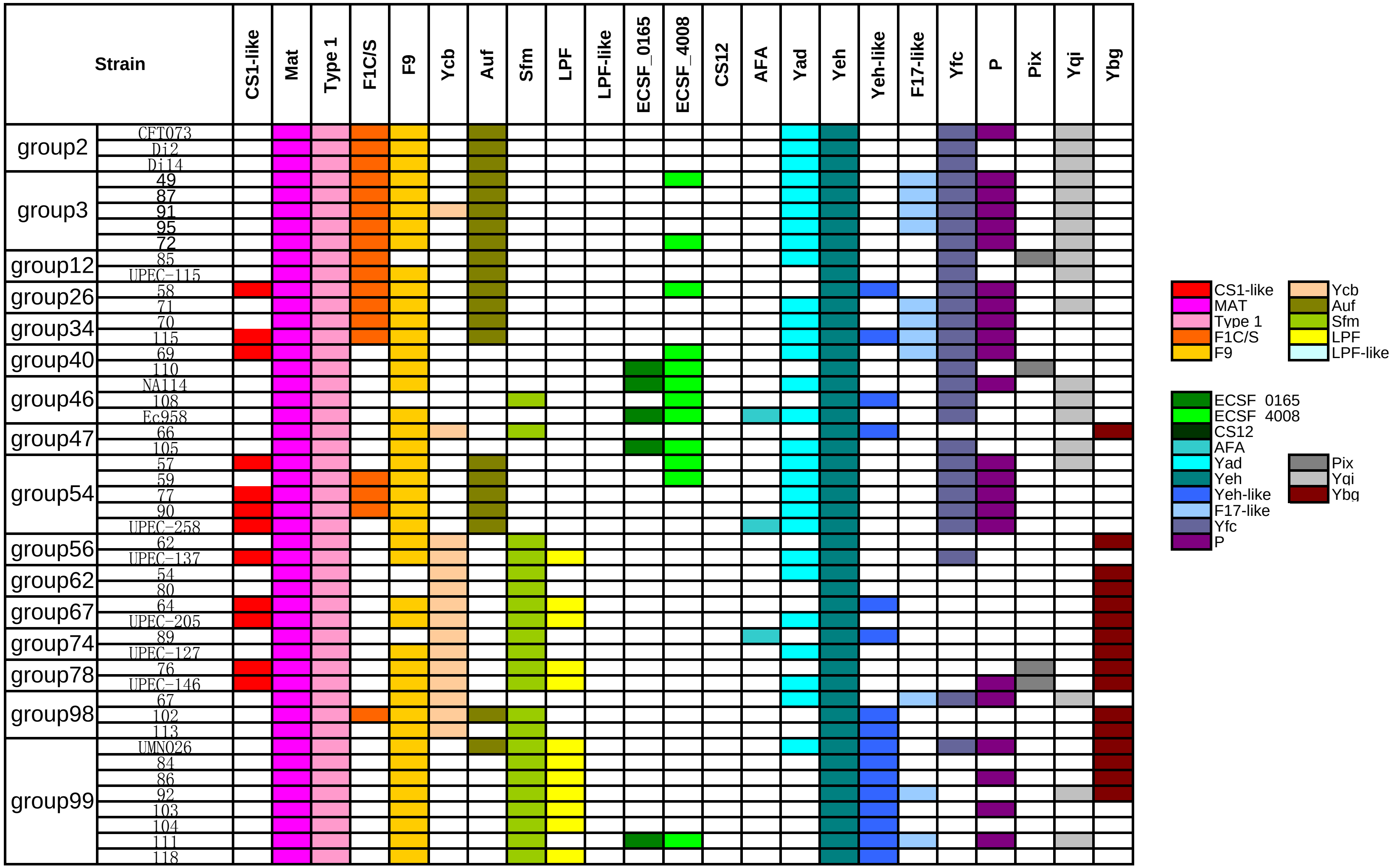

Fig. S4. Distribution of chaperone-usher fimbrial operons in strains not separated by the four genes (*yagV*, *fimF/H* and *fumC*). The name of the fimbriae is shown at the top. Each color box is representative of a certain type of chaperone-usher fimbrial operon, as indicated below the figure.
