# Supplementary material for: A High-resolution Typing Assay for Uropathogenic Escherichia coli Based on Fimbrial Diversity
Source: Front Microbiol. 2016 Apr 29;7:623. doi: 10.3389/fmicb.2016.00623 (PMC4850163; doi:10.3389/fmicb.2016.00623)
Supplement: Supplementary file 10 [file Image_5.PDF]

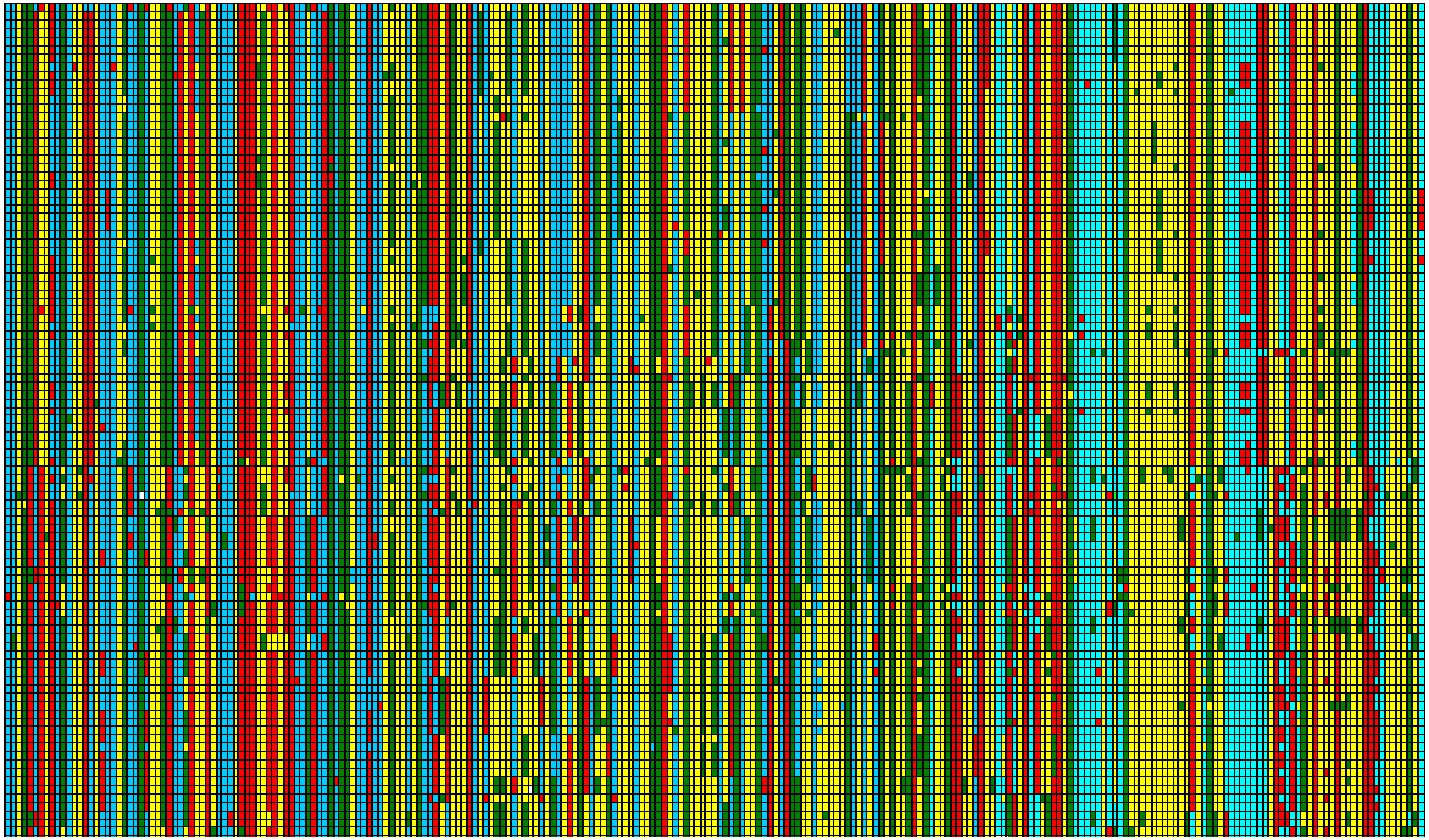

Fig. S5. The polymophism of the combined four genes (*yagV*, *fimF/H* and *fumC*) of the 130 UPEC strains. The horizontal direction means 299 positions, and the longitudinal direction means 99 groups separated. Red represents A, green represents T, yellow represents C, and blue represents G.
